# Supplementary material for: Nickel-catalyzed reductive coupling of homoenolates and their higher homologues with unactivated alkyl bromides
Source: Nat Commun. 2020 Nov 6;11:5638. doi: 10.1038/s41467-020-19194-x (PMC7648641; doi:10.1038/s41467-020-19194-x)
Supplement: Supplementary file 1 — Supplementary Information [file 41467_2020_19194_MOESM1_ESM.pdf]

Nickel-Catalyzed Reductive Coupling of Homoenolates and Their Higher Homologues with Unactivated Alkyl Bromides

Lin *et al.*

Supplementary Figure 1. ^{1}H **NMR Spectrum of 3aa (400 MHz,** $CDCl_3$**)**

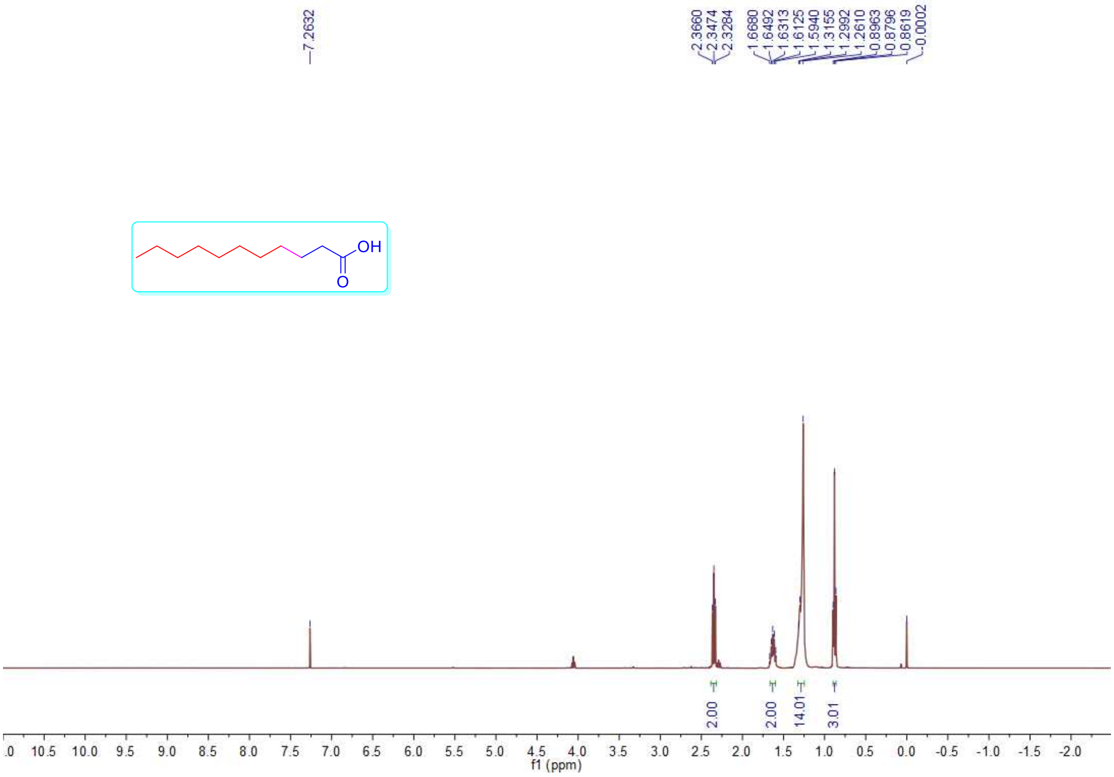

Supplementary Figure 2. ^{13}C **NMR Spectrum of 3aa (101 MHz,** $CDCl_3$**)**

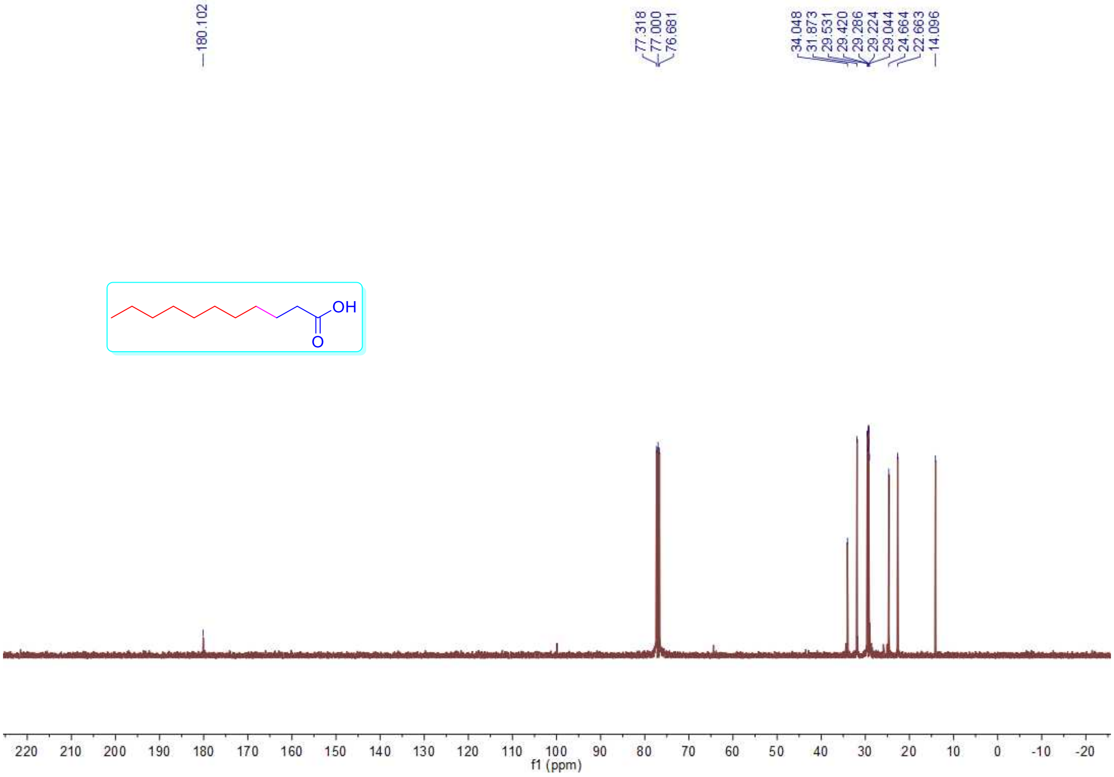

Supplementary Figure 3. ^{1}H NMR Spectrum of 3ab (400 MHz, $CDCl_3$)

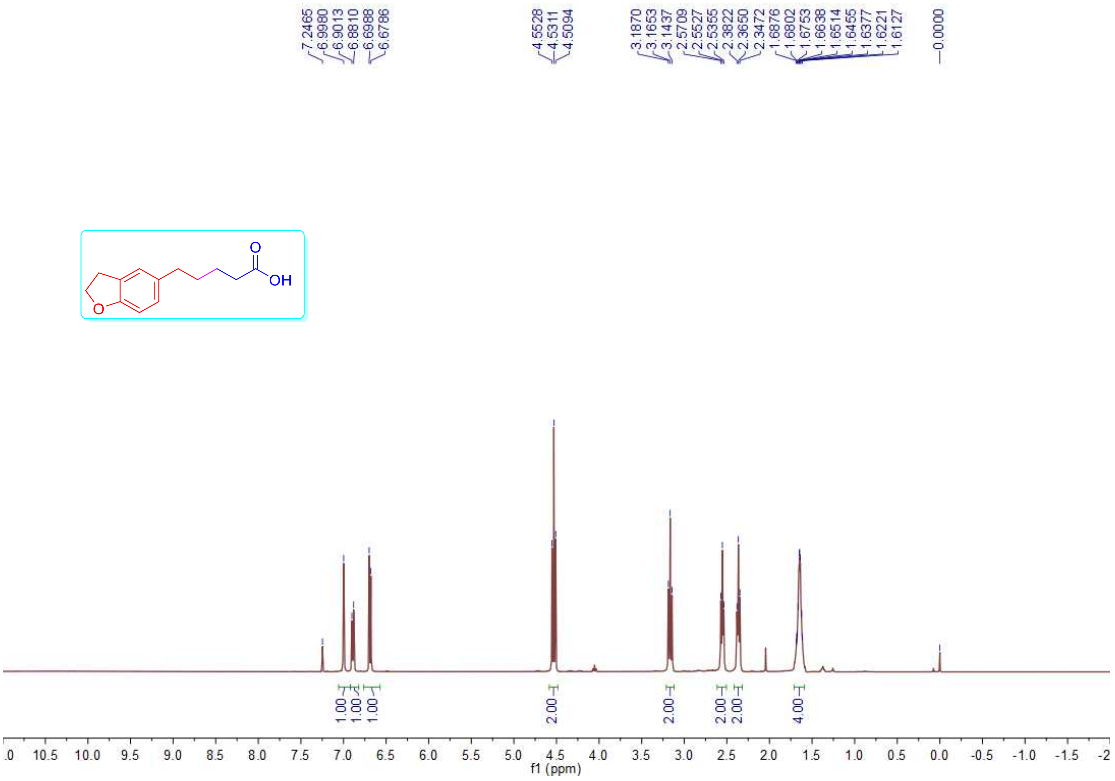

Supplementary Figure 4. ^{13}C NMR Spectrum of 3ab (101 MHz, $CDCl_3$)

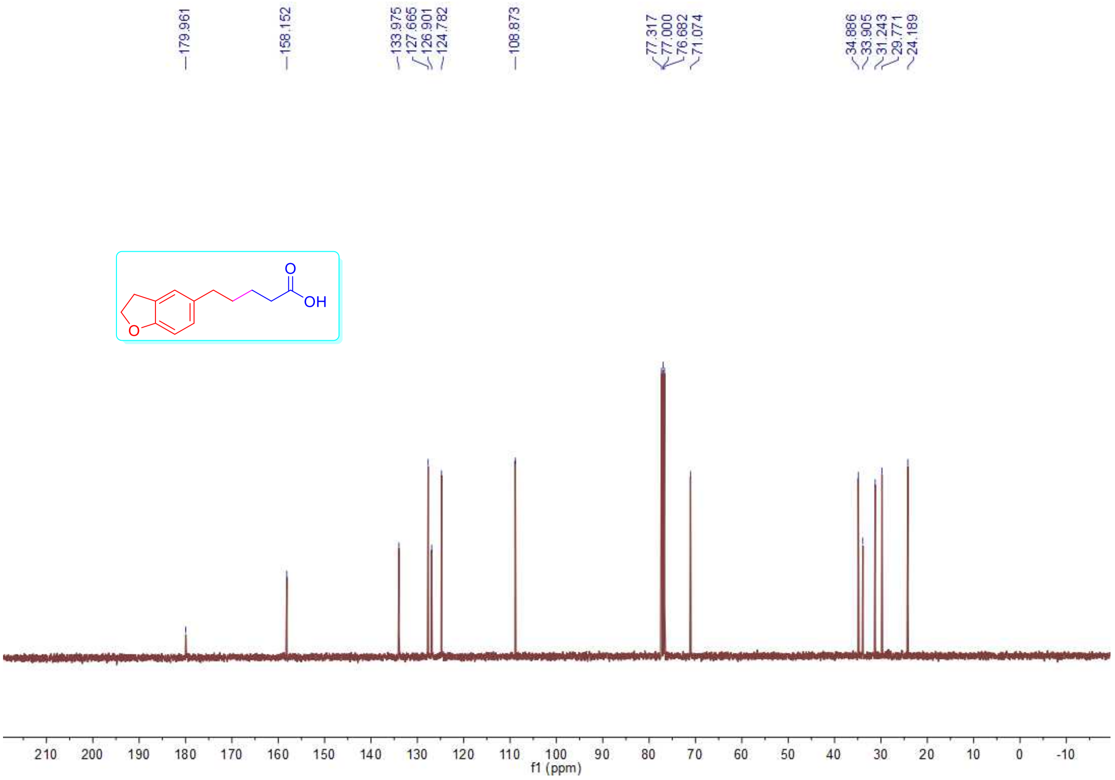

Supplementary Figure 5. ^{1}H NMR Spectrum of 3ac (400 MHz, $CDCl_3$)

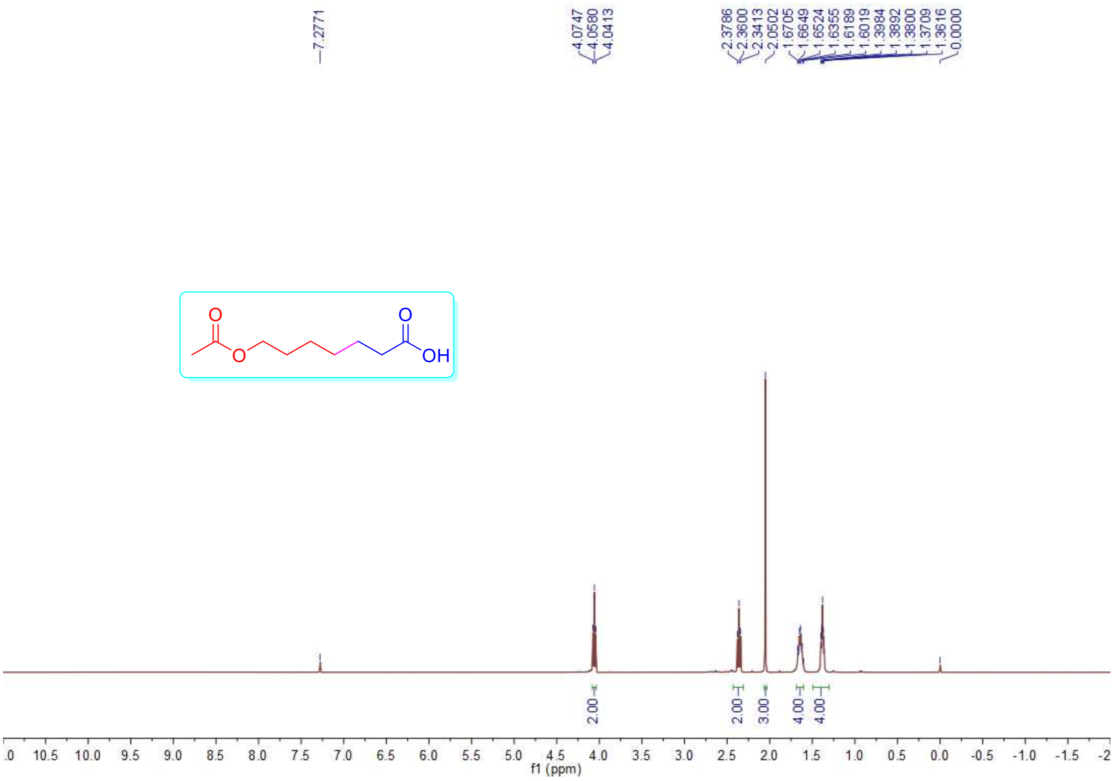

Supplementary Figure 6. ^{13}C NMR Spectrum of 3ac (101 MHz, $CDCl_3$)

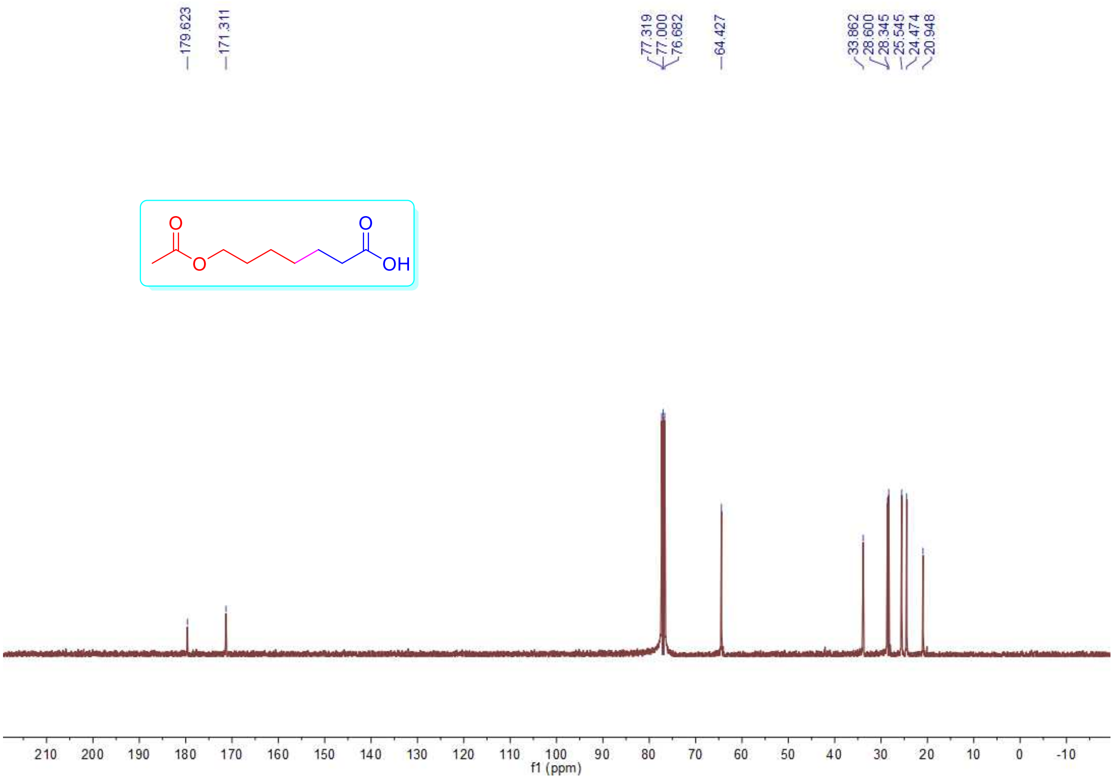

Supplementary Figure 7. ^{1}H NMR Spectrum of 3ad (400 MHz, $CDCl_3$)

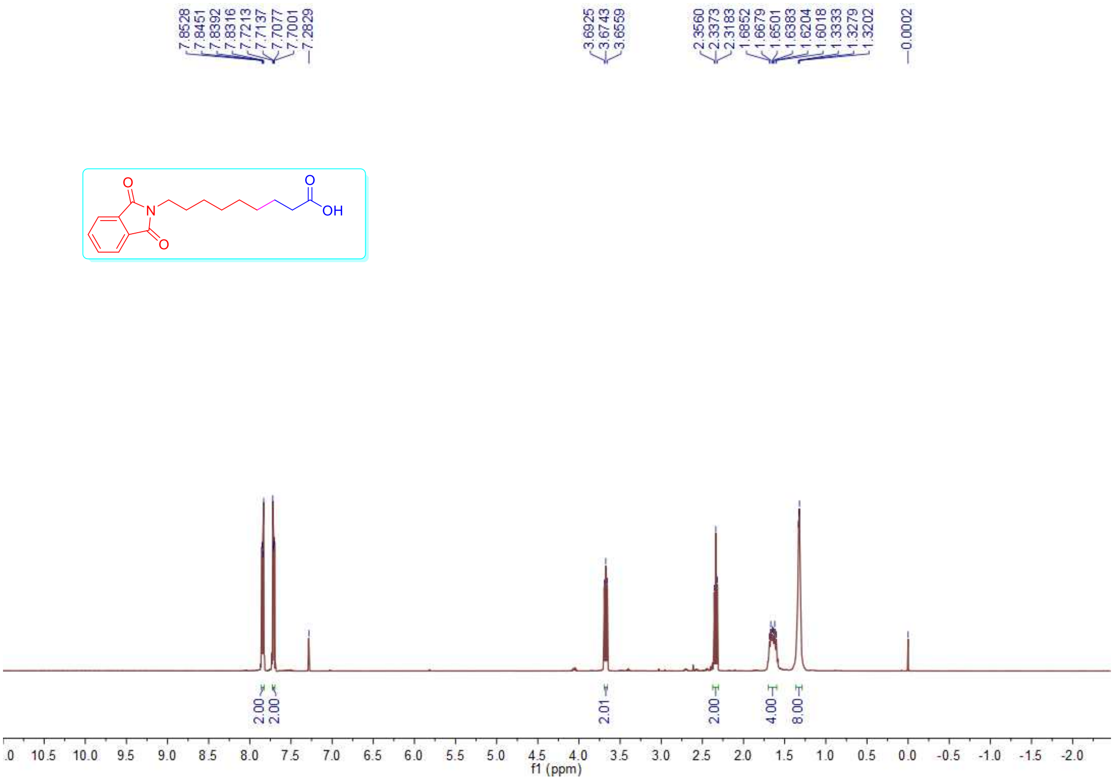

Supplementary Figure 8. ^{13}C NMR Spectrum of 3ad (101 MHz, $CDCl_3$)

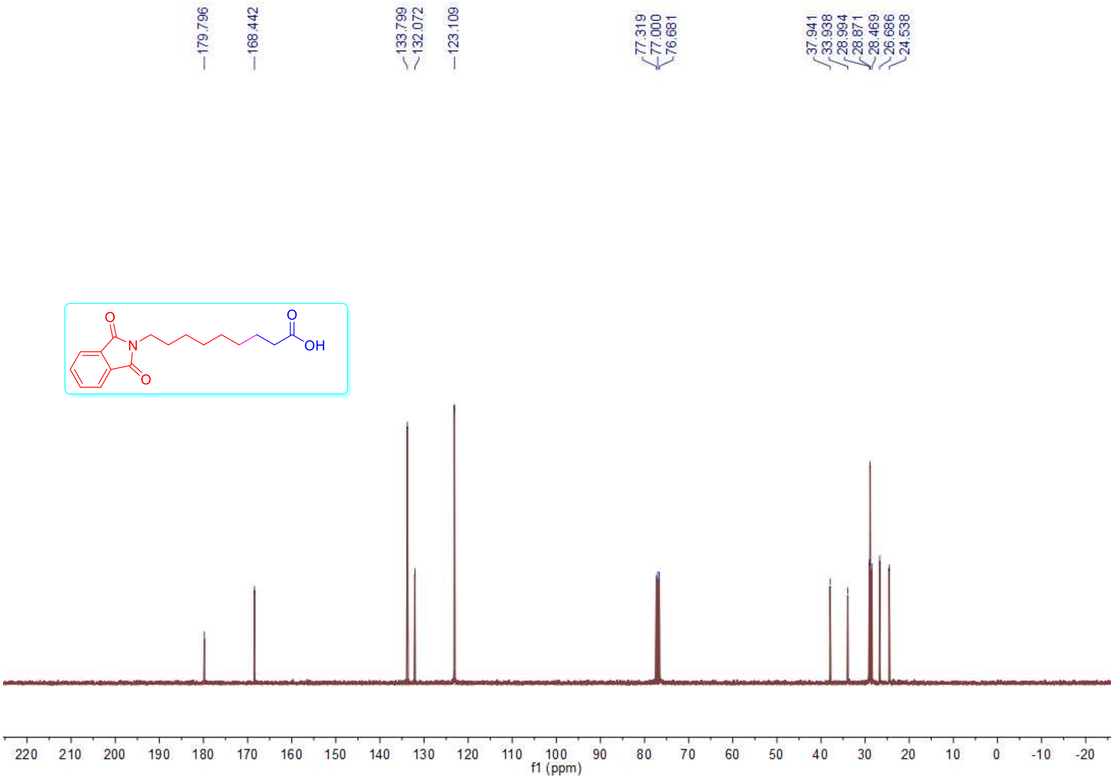

Supplementary Figure 9. ^{1}**H NMR Spectrum of 3ae (400 MHz, CDCl$_3$)**

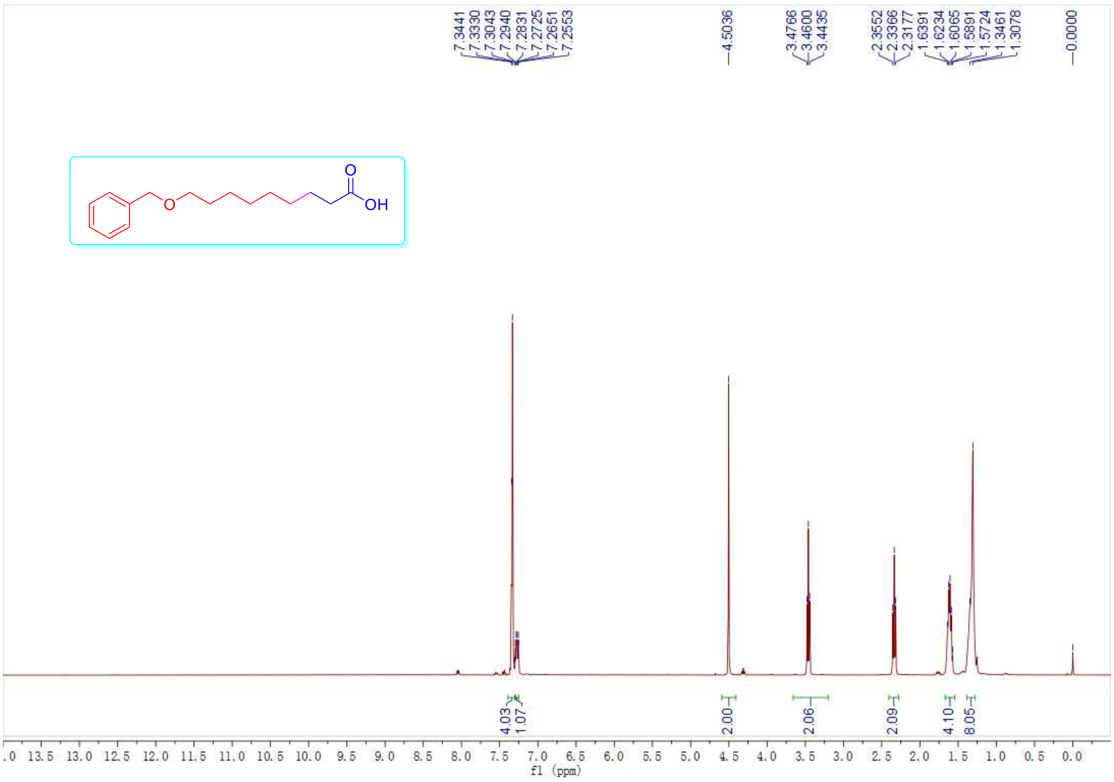

Supplementary Figure 10. ^{13}**C NMR Spectrum of 3ae (101 MHz, CDCl$_3$)**

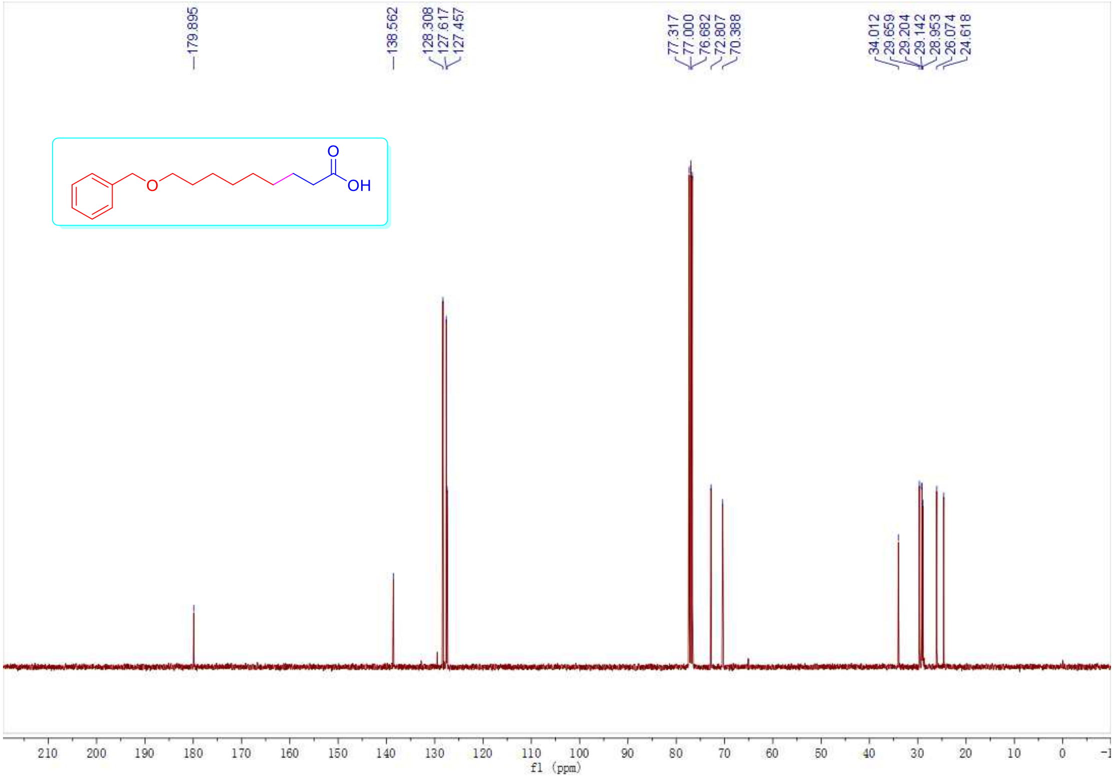

Supplementary Figure 11. ^{1}**H NMR Spectrum of 3af (400 MHz, CDCl$_3$)**

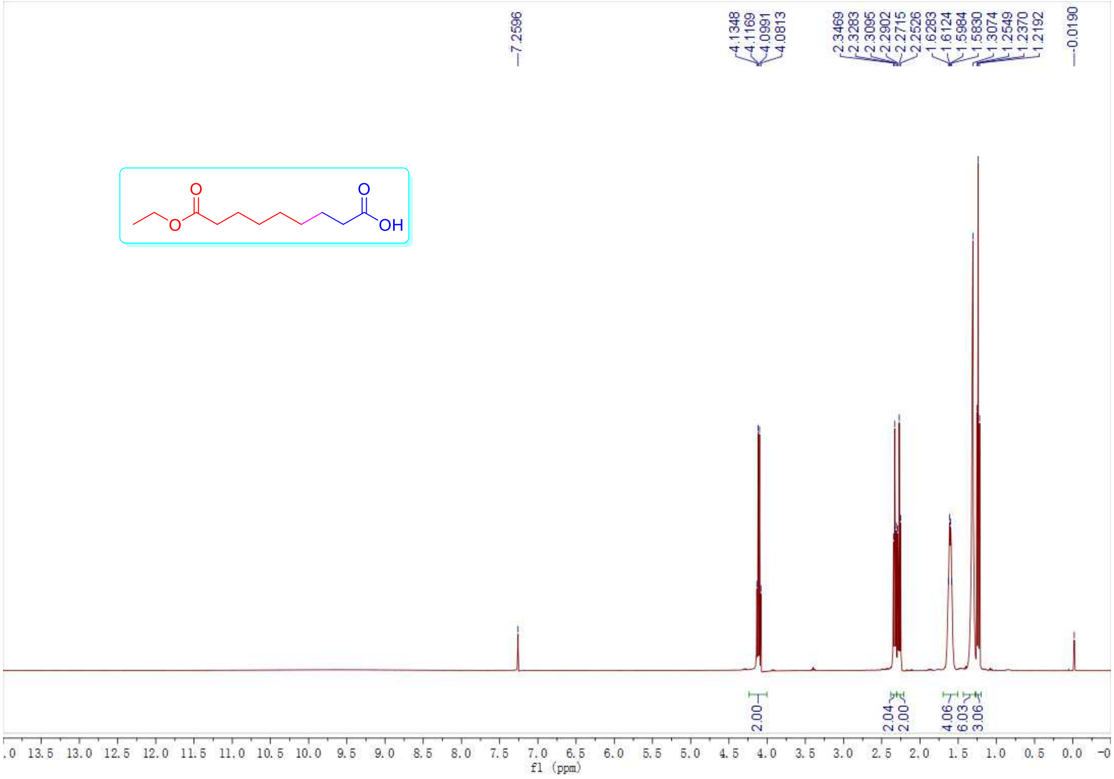

Supplementary Figure 12. ^{13}**C NMR Spectrum of 3af (101 MHz, CDCl$_3$)**

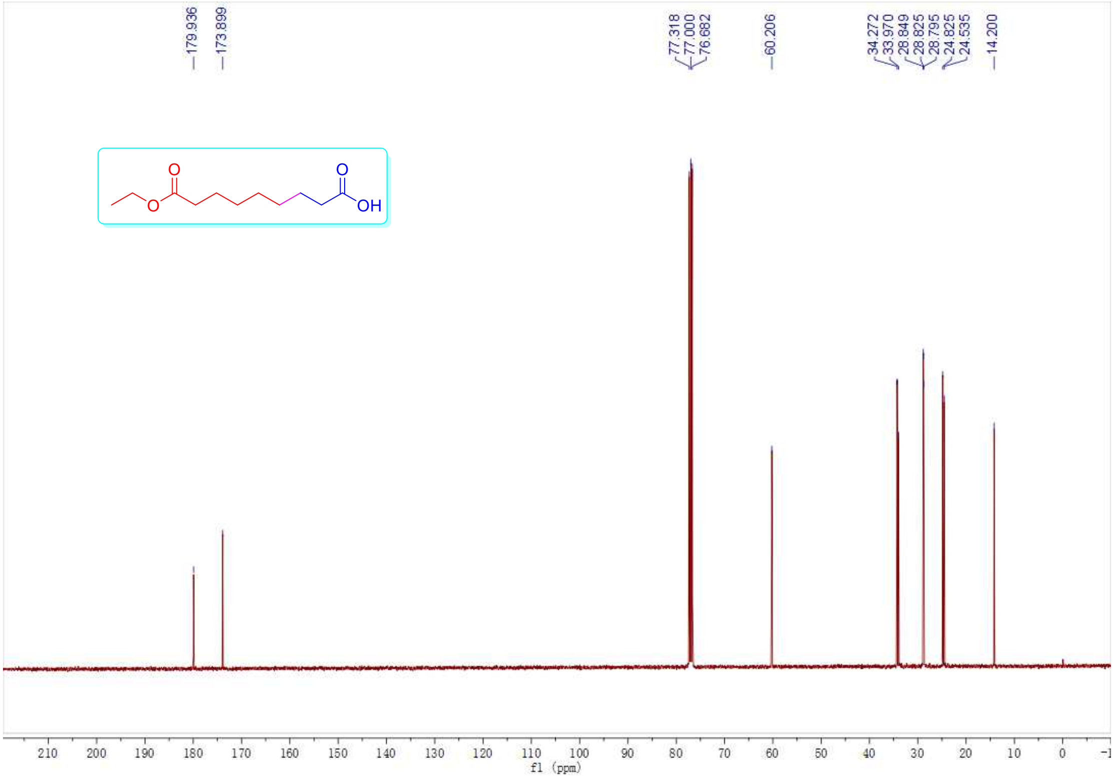

Supplementary Figure 13. ^{1}H NMR Spectrum of 3an (400 MHz, $CDCl_3$)

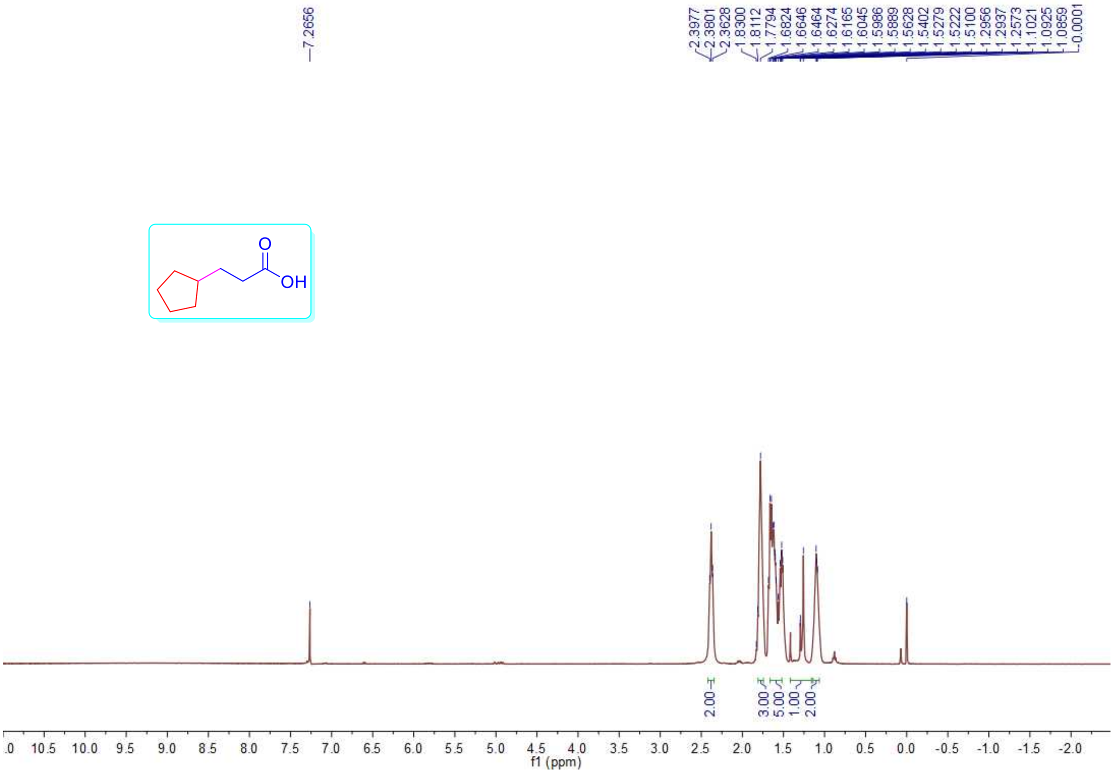

Supplementary Figure 14. ^{13}C NMR Spectrum of 3an (101 MHz, $CDCl_3$)

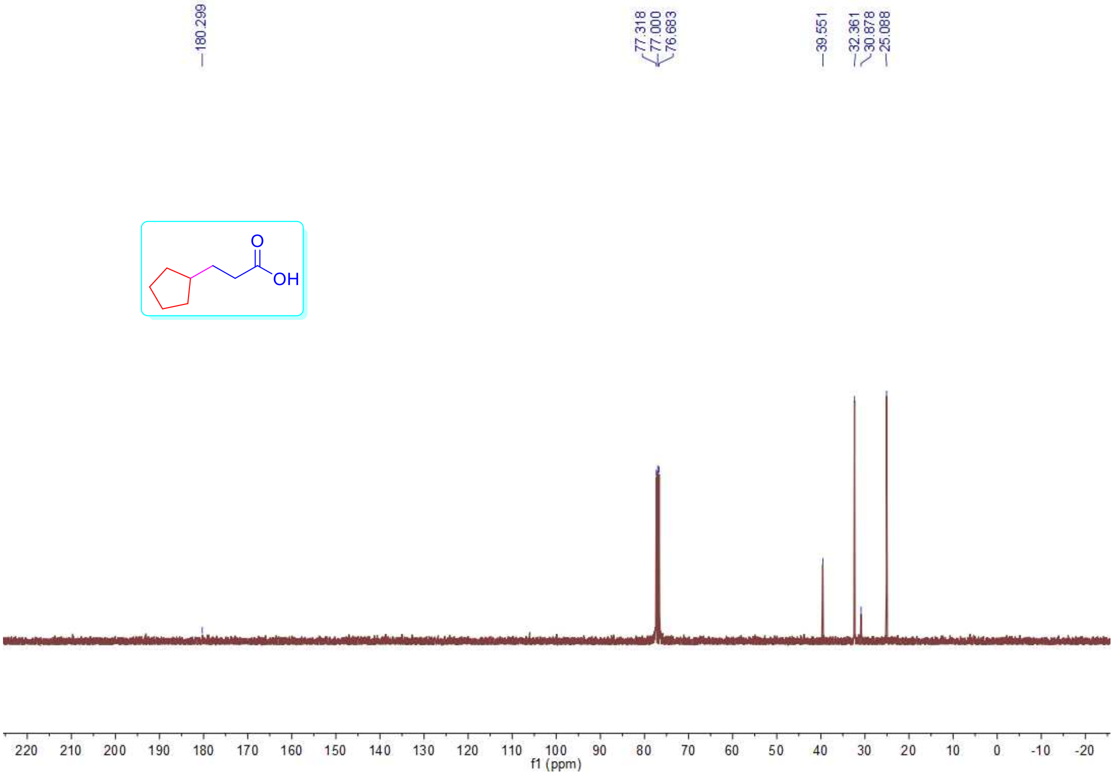

Supplementary Figure 15. ^{1}H NMR Spectrum of 3ao (400 MHz, $CDCl_3$)

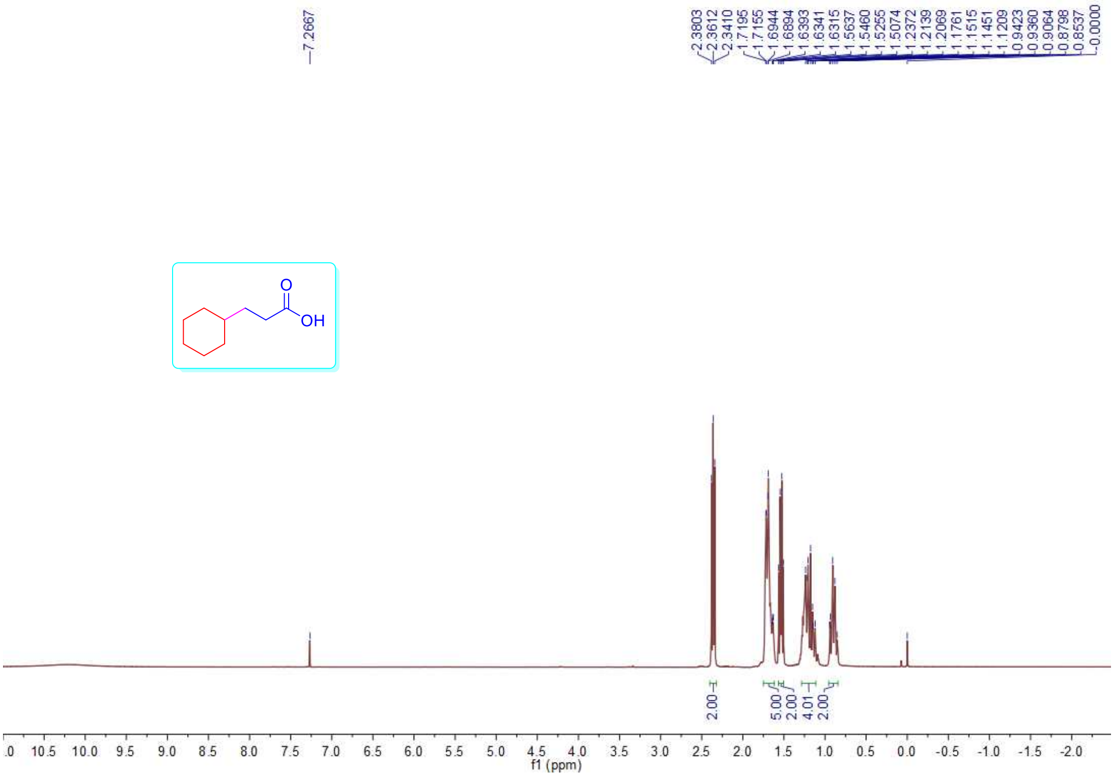

Supplementary Figure 16. ^{13}C NMR Spectrum of 3ao (101 MHz, $CDCl_3$)

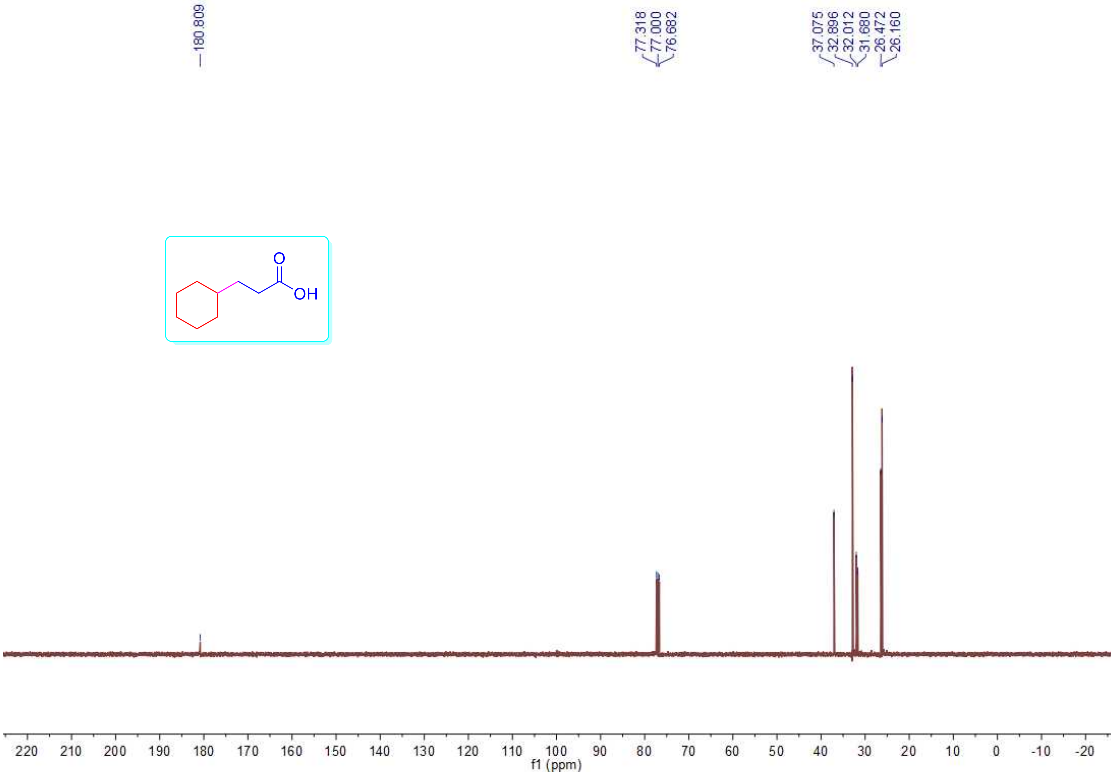

Supplementary Figure 17. ^{1}H NMR Spectrum of 4bg (400 MHz, $CDCl_3$)

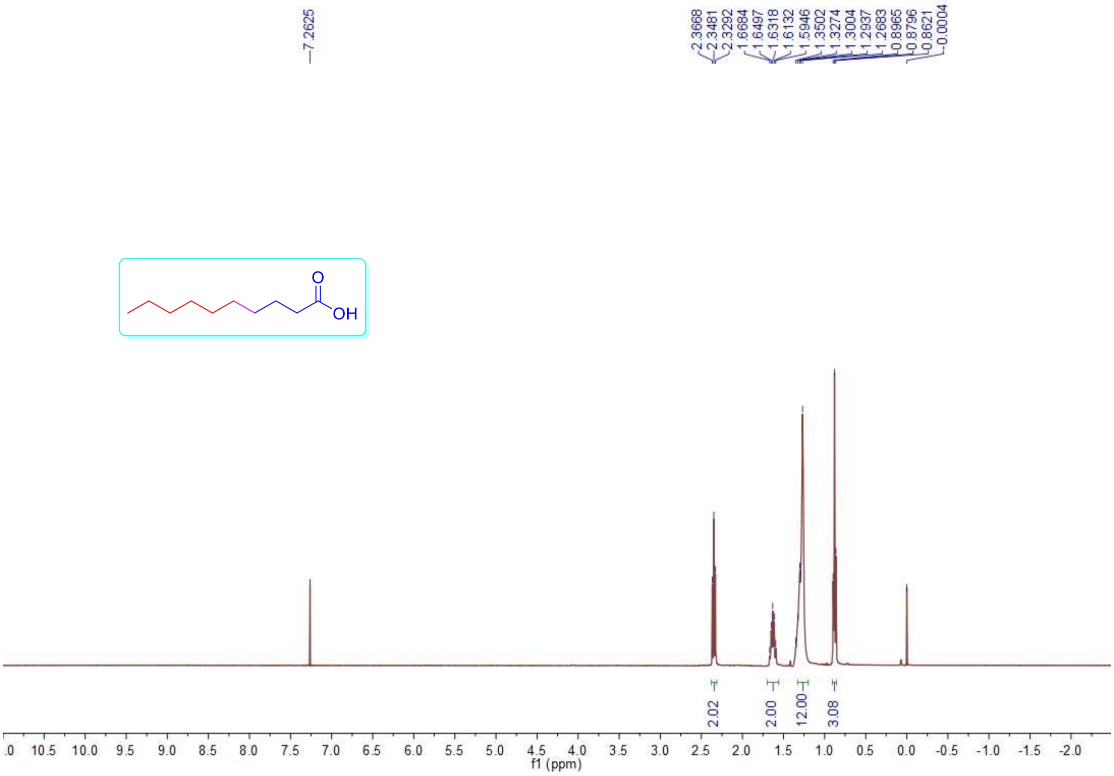

Supplementary Figure 18. ^{13}C NMR Spectrum of 4bg (101 MHz, $CDCl_3$)

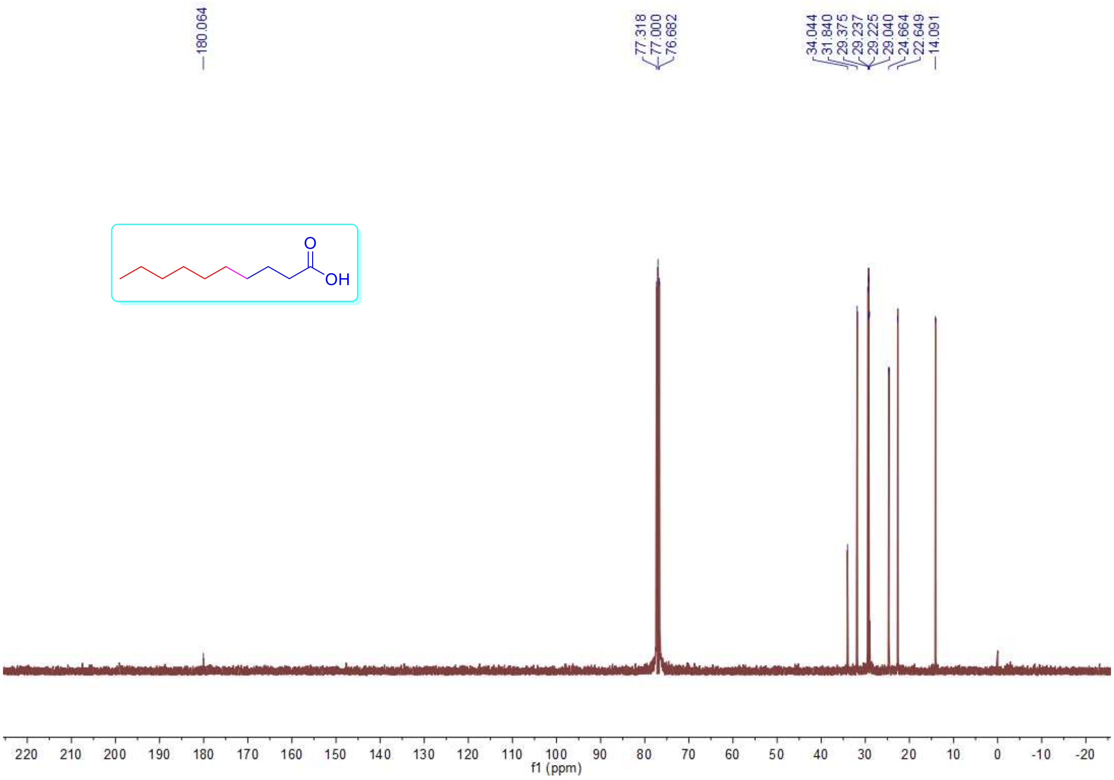

Supplementary Figure 19. ^{1}H NMR Spectrum of 4ba (400 MHz, $CDCl_3$)

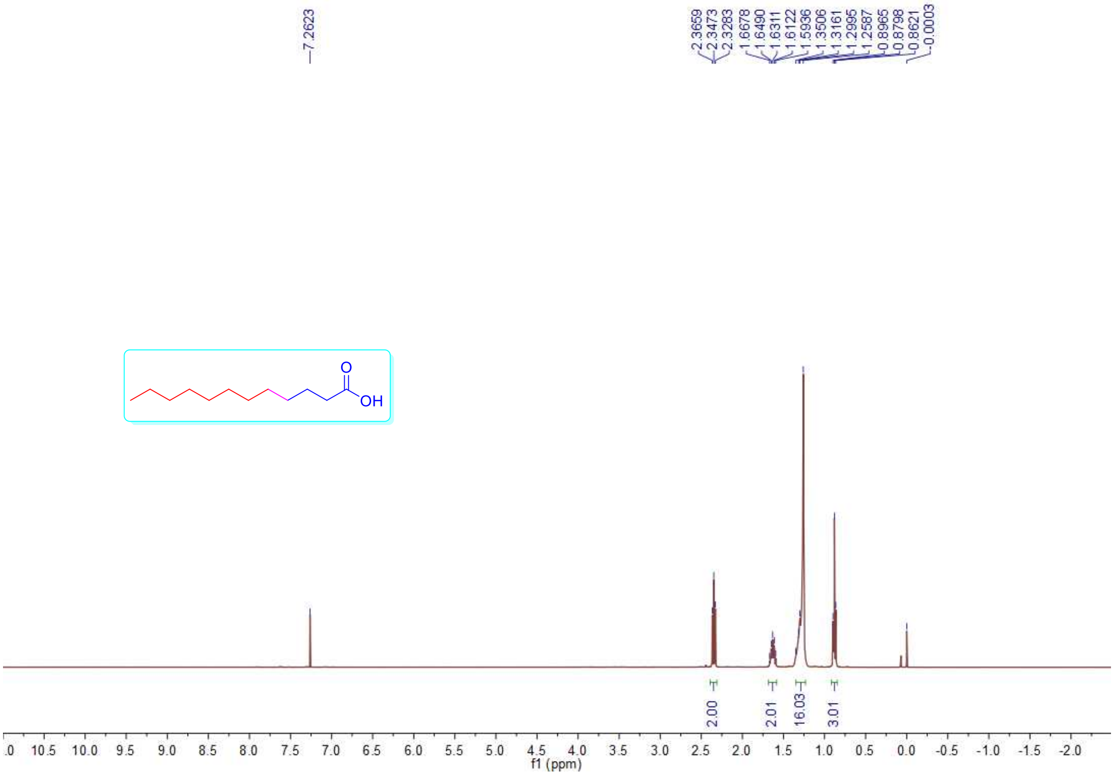

Supplementary Figure 20. ^{13}C NMR Spectrum of 4ba (101 MHz, $CDCl_3$)

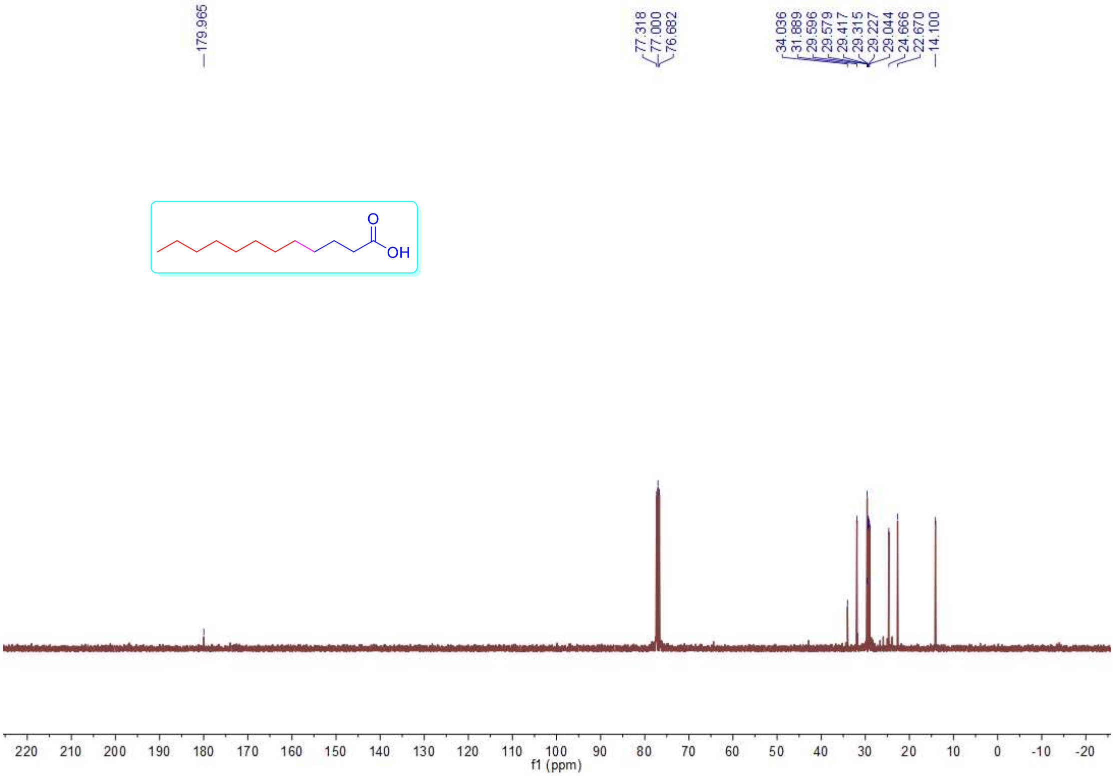

Supplementary Figure 21. ^{1}H NMR Spectrum of 4bb (400 MHz, $CDCl_3$)

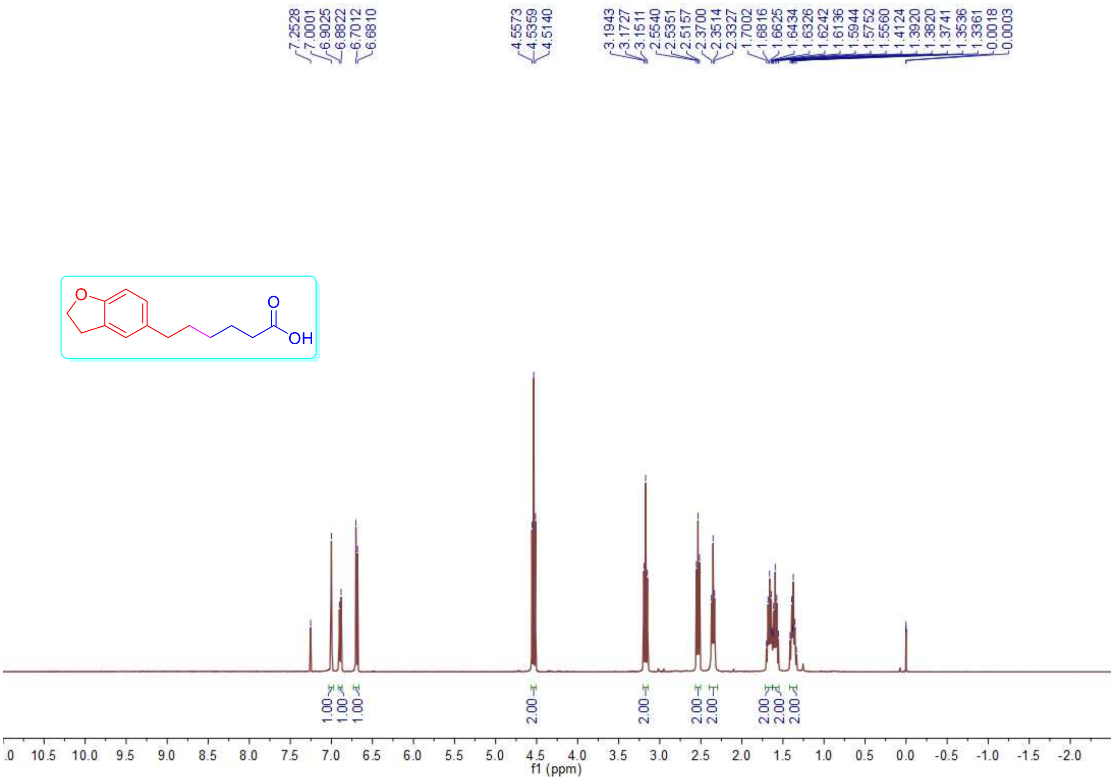

Supplementary Figure 22. ^{13}C NMR Spectrum of 4bb (101 MHz, $CDCl_3$)

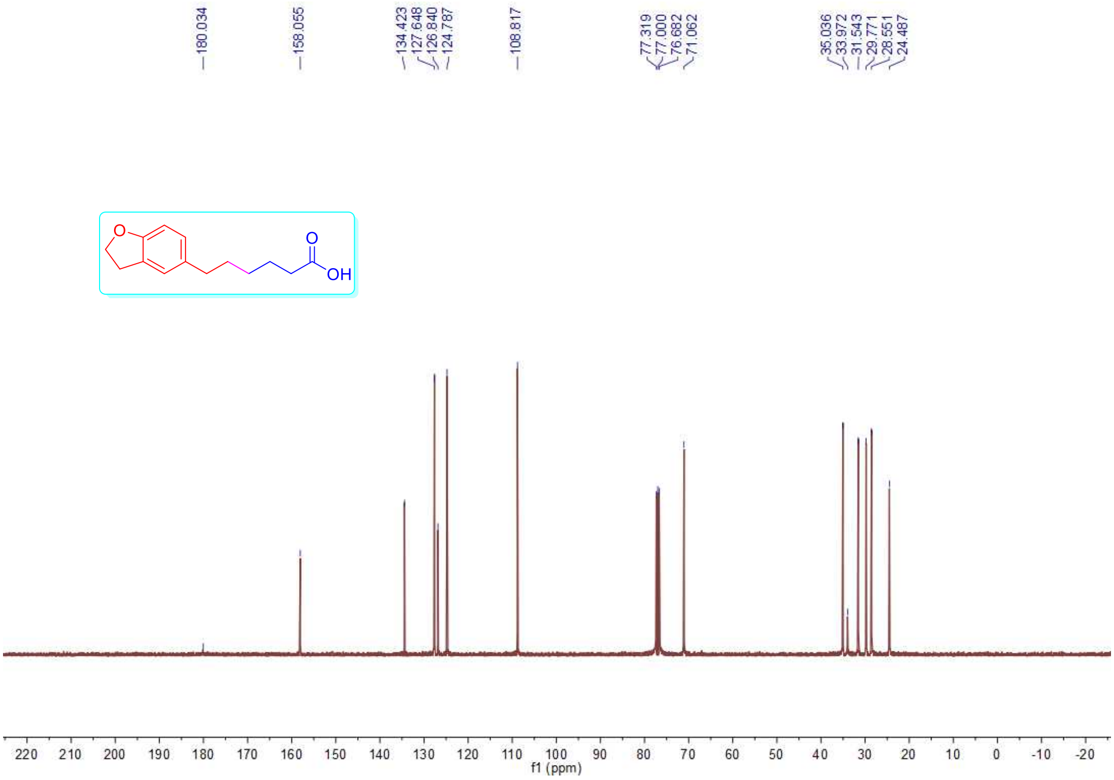

Supplementary Figure 23. ^{1}H NMR Spectrum of 4bc (400 MHz, $CDCl_3$)

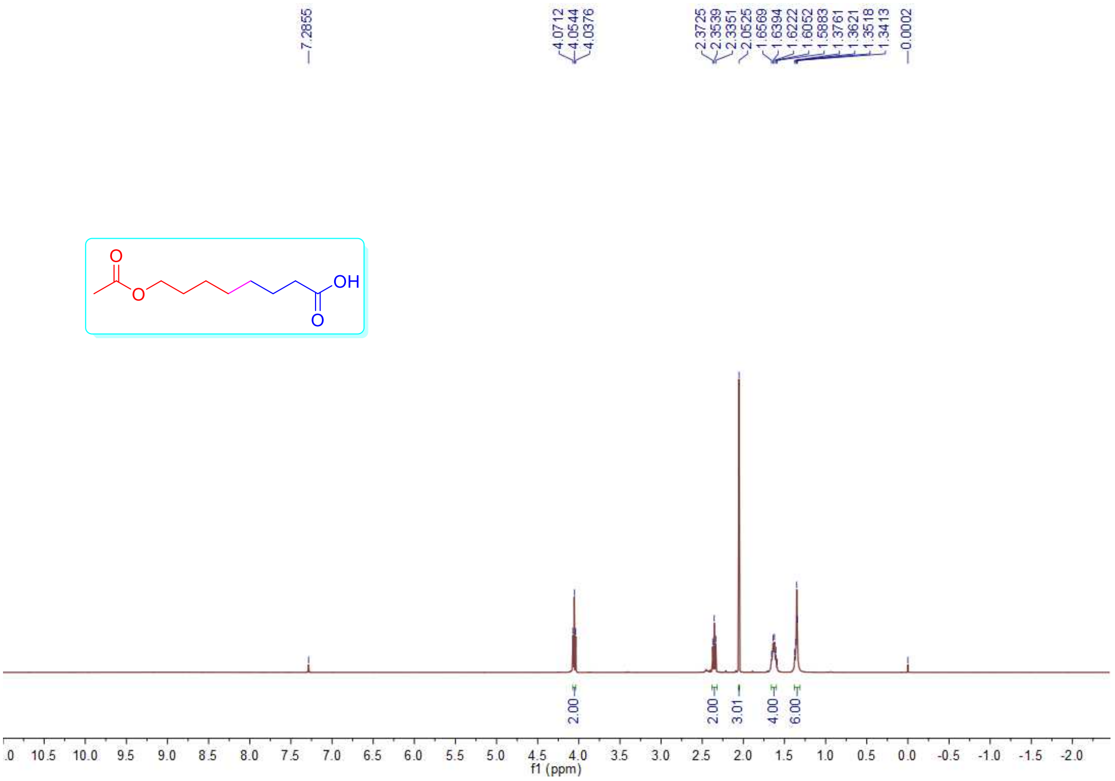

Supplementary Figure 24. ^{13}C NMR Spectrum of 4bc (101 MHz, $CDCl_3$)

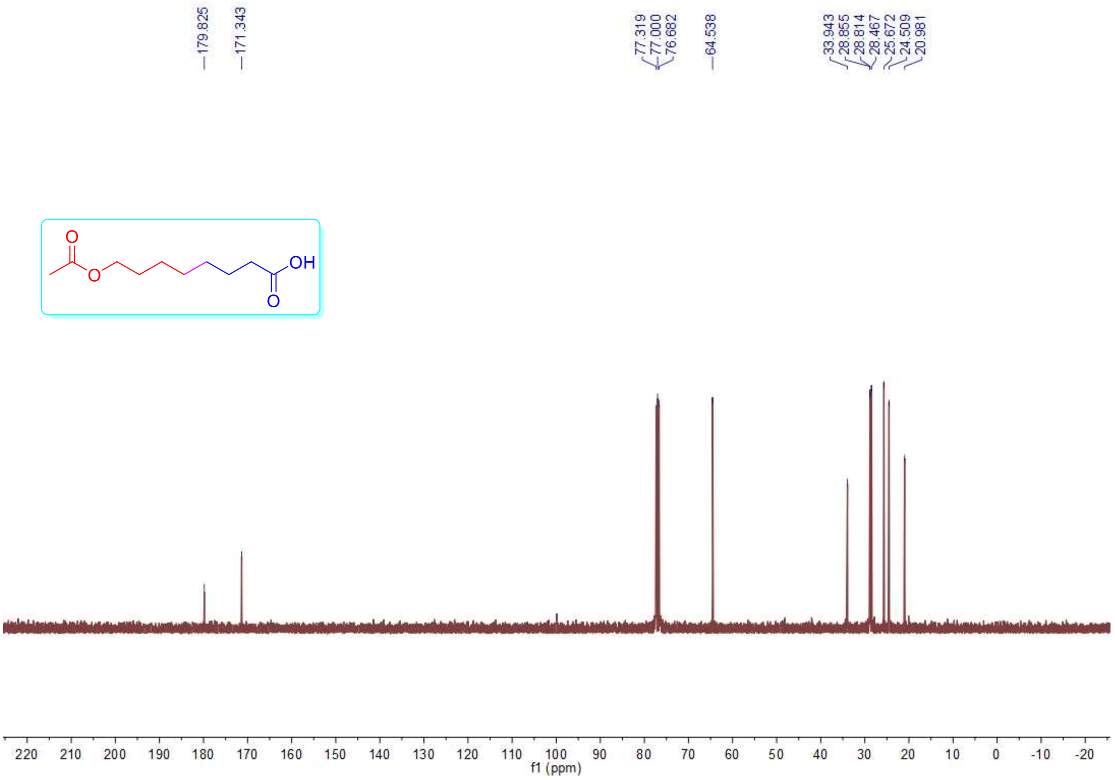

Supplementary Figure 25. ^{1}H NMR Spectrum of 4bd (400 MHz, $CDCl_3$)

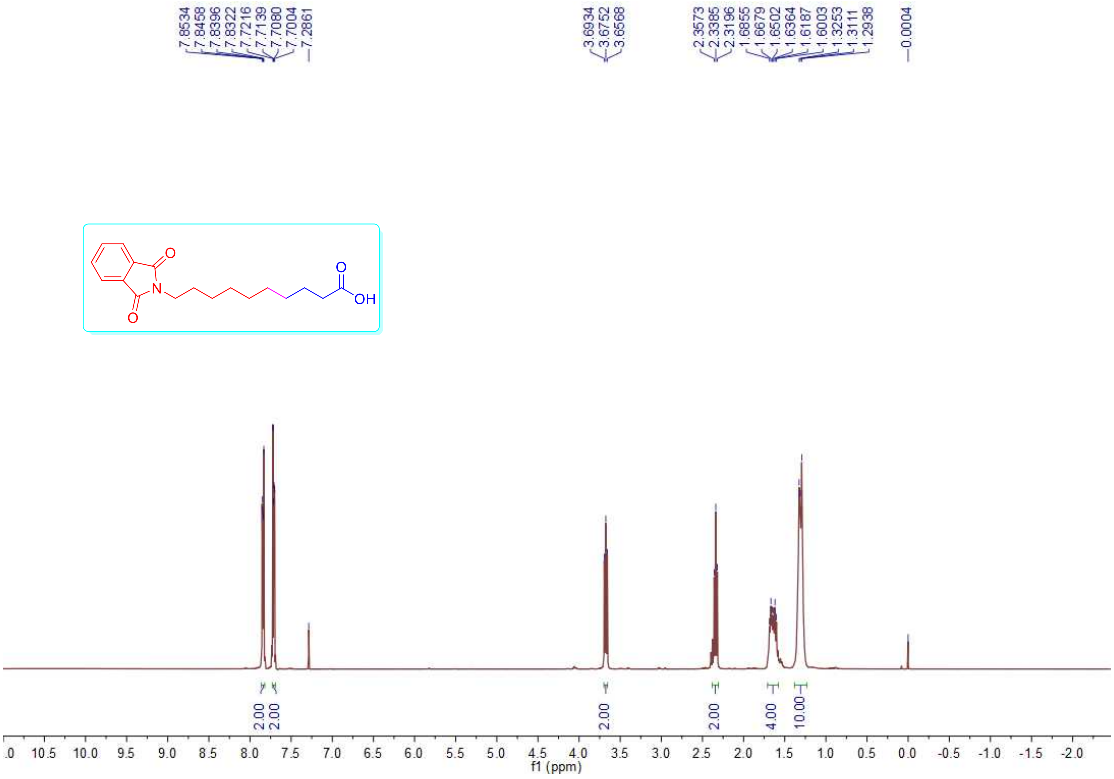

Supplementary Figure 26. ^{13}C NMR Spectrum of 4bd (101 MHz, $CDCl_3$)

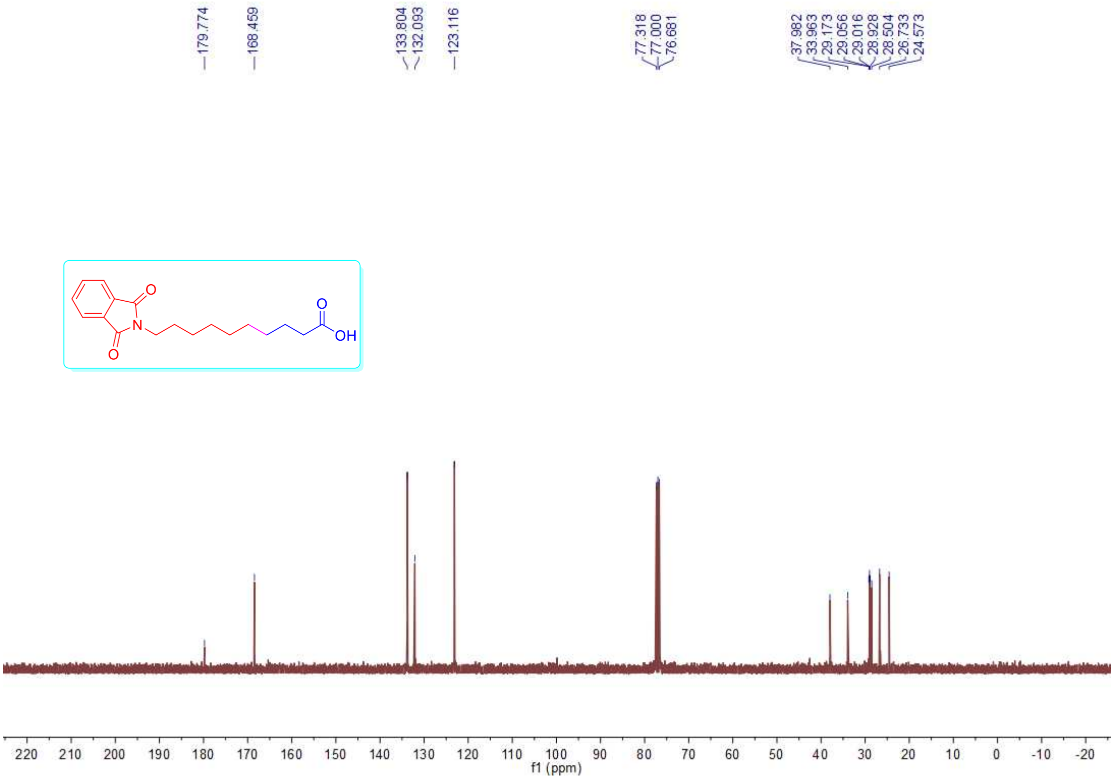

Supplementary Figure 27. ^{1}**H NMR Spectrum of 4be (400 MHz, CDCl$_3$)**

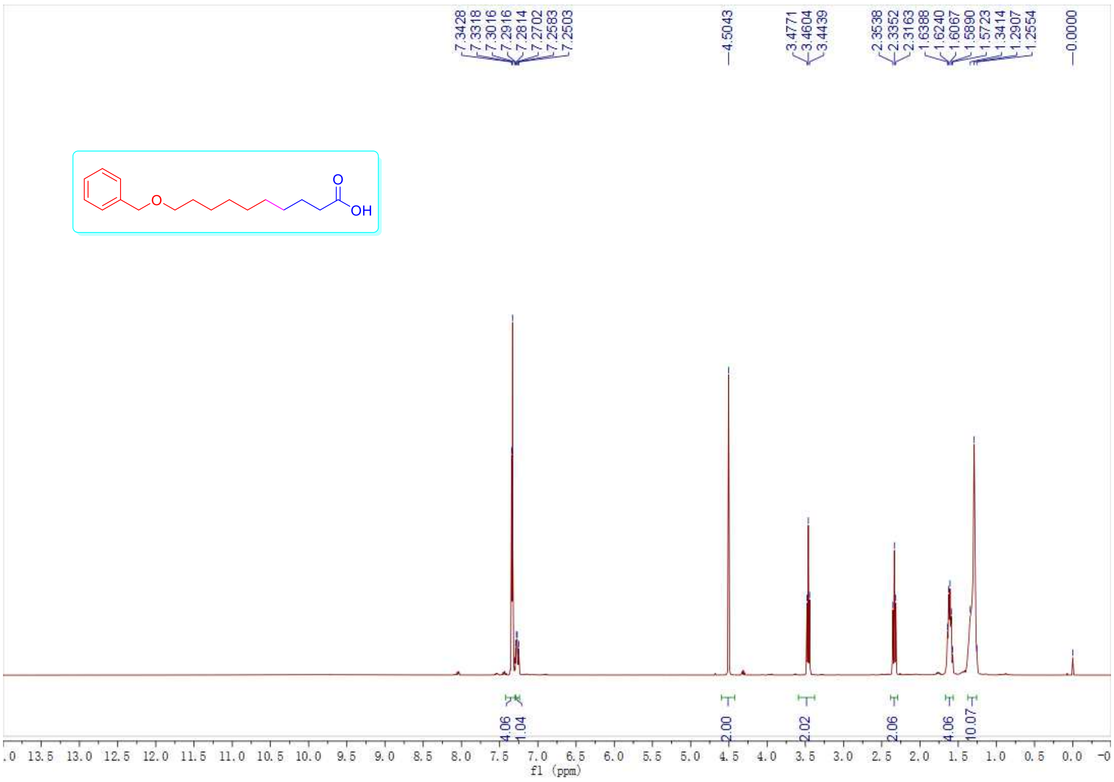

Supplementary Figure 28. ^{13}**C NMR Spectrum of 4be (101 MHz, CDCl$_3$)**

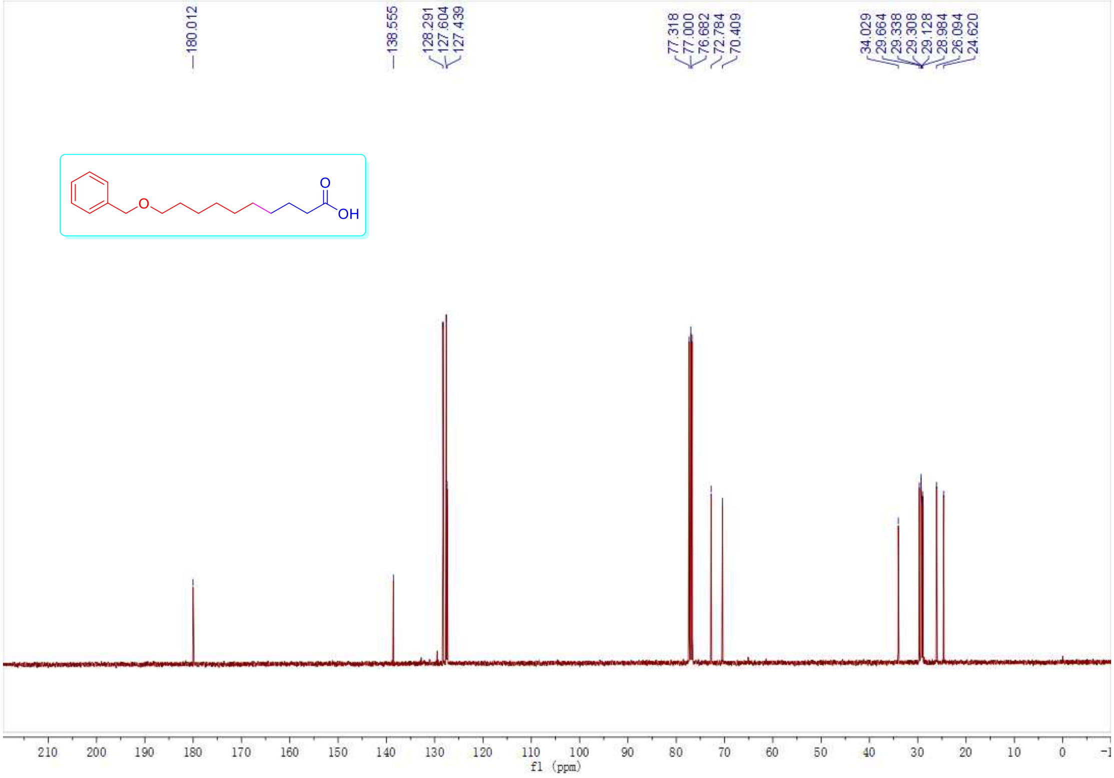

Supplementary Figure 29. ^{1}H NMR Spectrum of 4bf (400 MHz, $CDCl_3$)

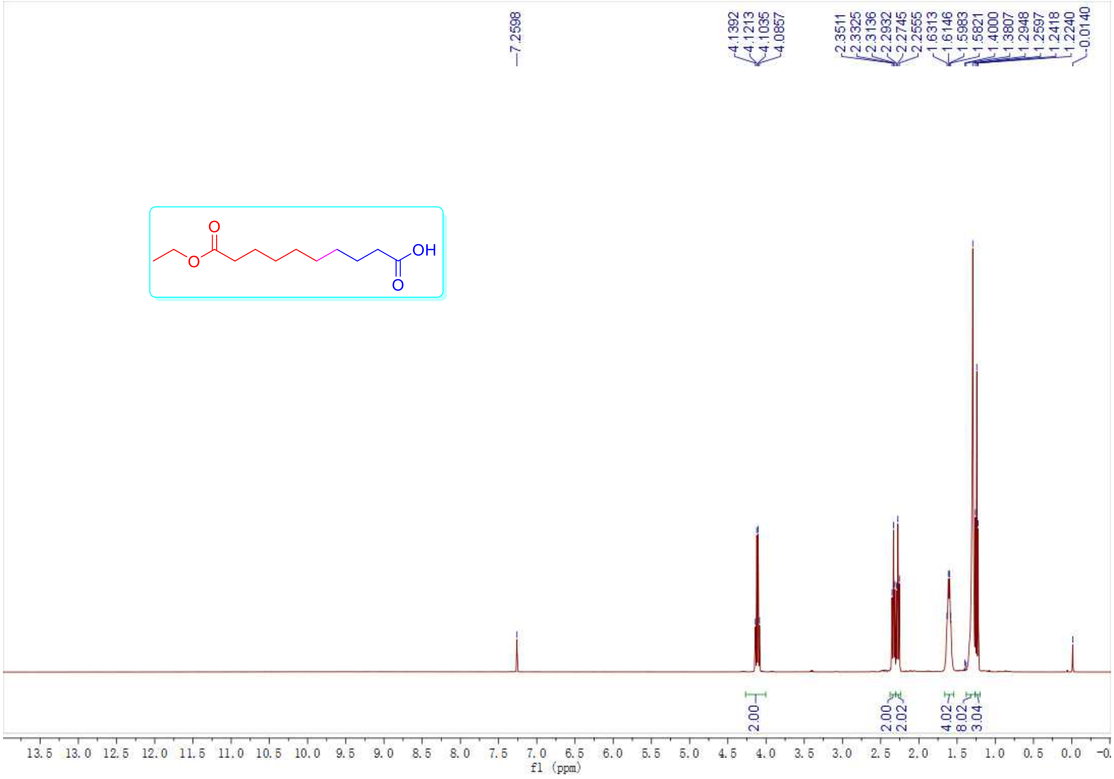

Supplementary Figure 30. ^{13}C NMR Spectrum of 4bf (101 MHz, $CDCl_3$)

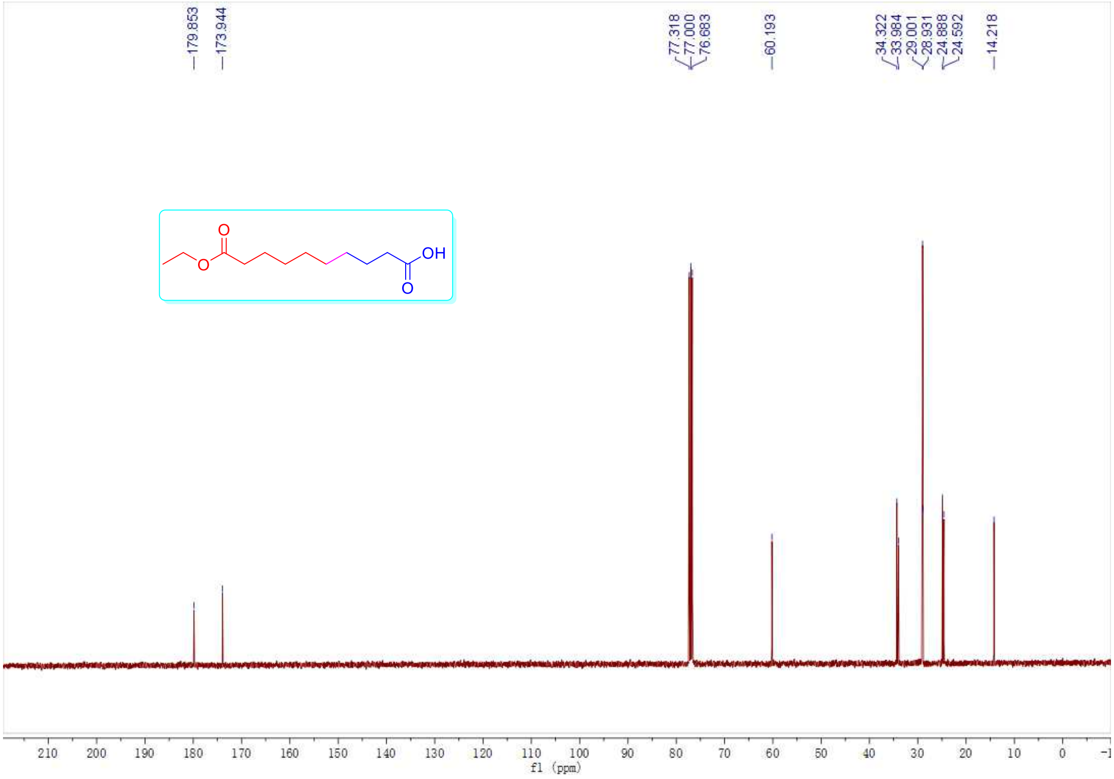

Supplementary Figure 31. ^{1}H NMR Spectrum of 4bh (400 MHz, $CDCl_3$)

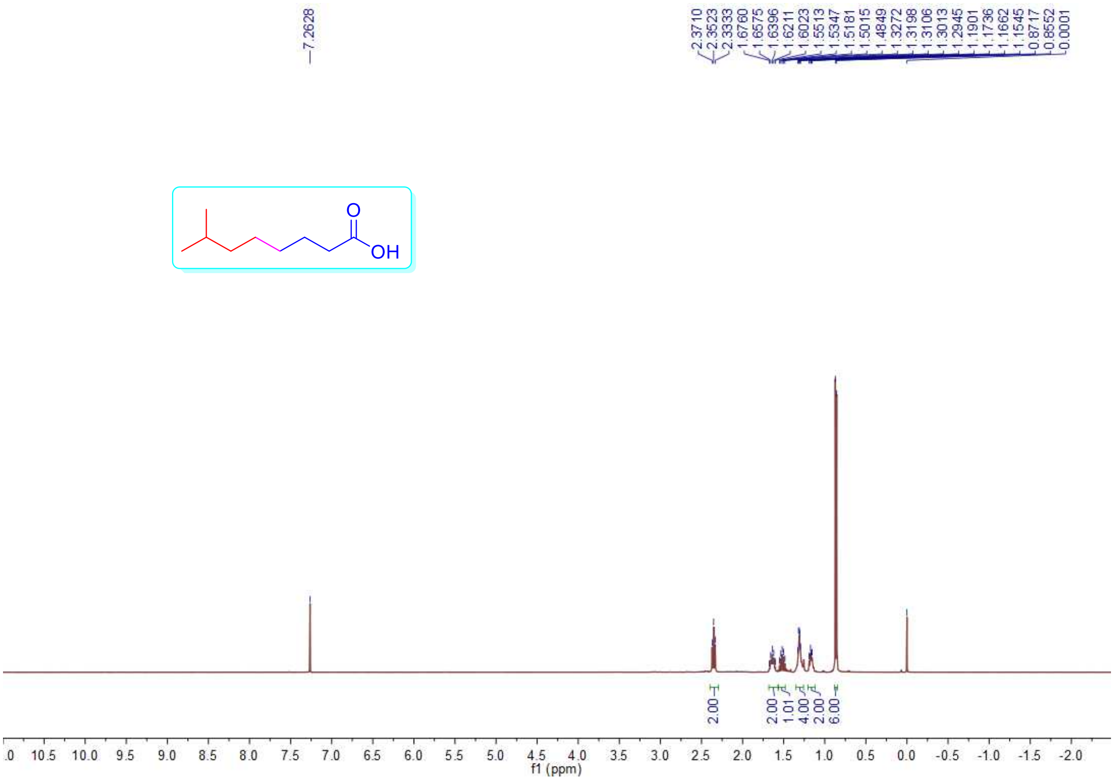

Supplementary Figure 32. ^{13}C NMR Spectrum of 4bh (101 MHz, $CDCl_3$)

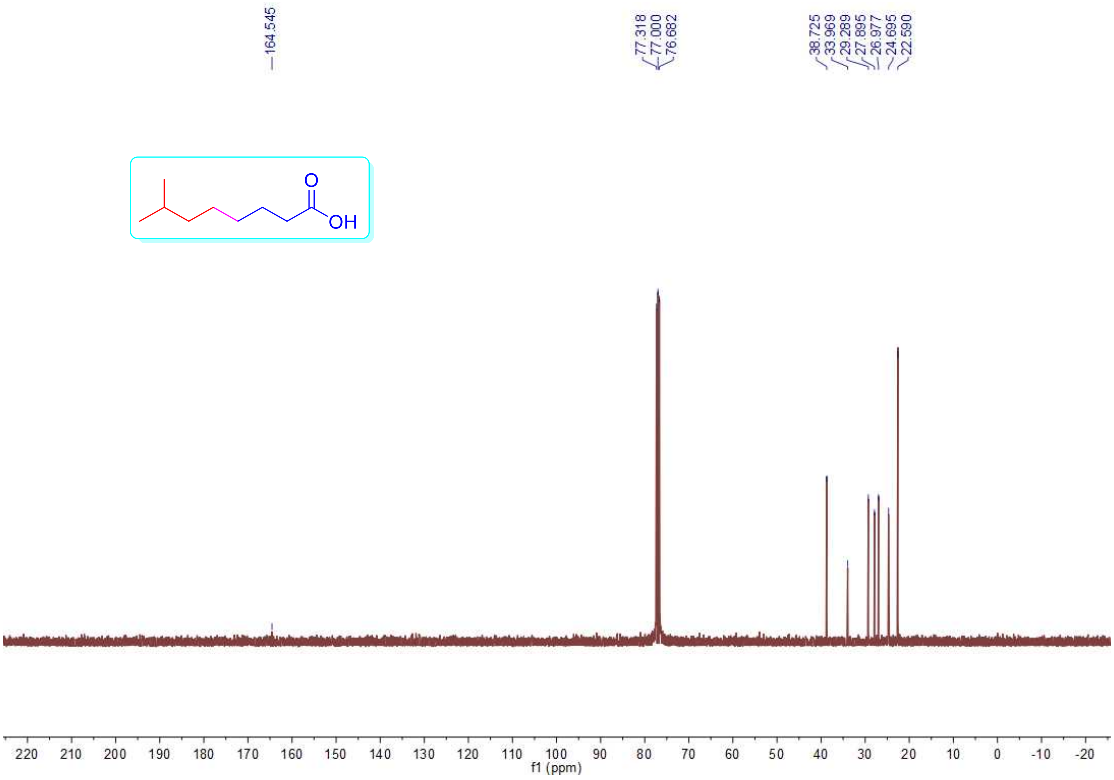

Supplementary Figure 33. ^{1}H NMR Spectrum of 4bi (400 MHz, $CDCl_3$)

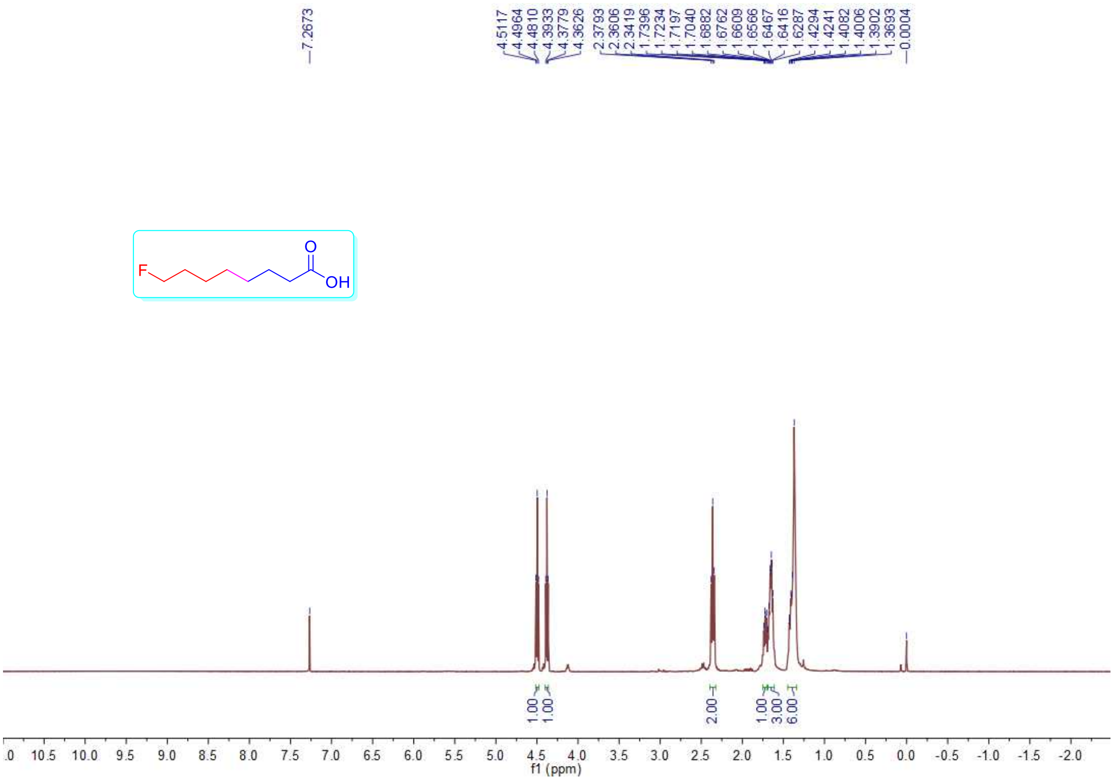

Supplementary Figure 34. ^{13}C NMR Spectrum of 4bi (101 MHz, $CDCl_3$)

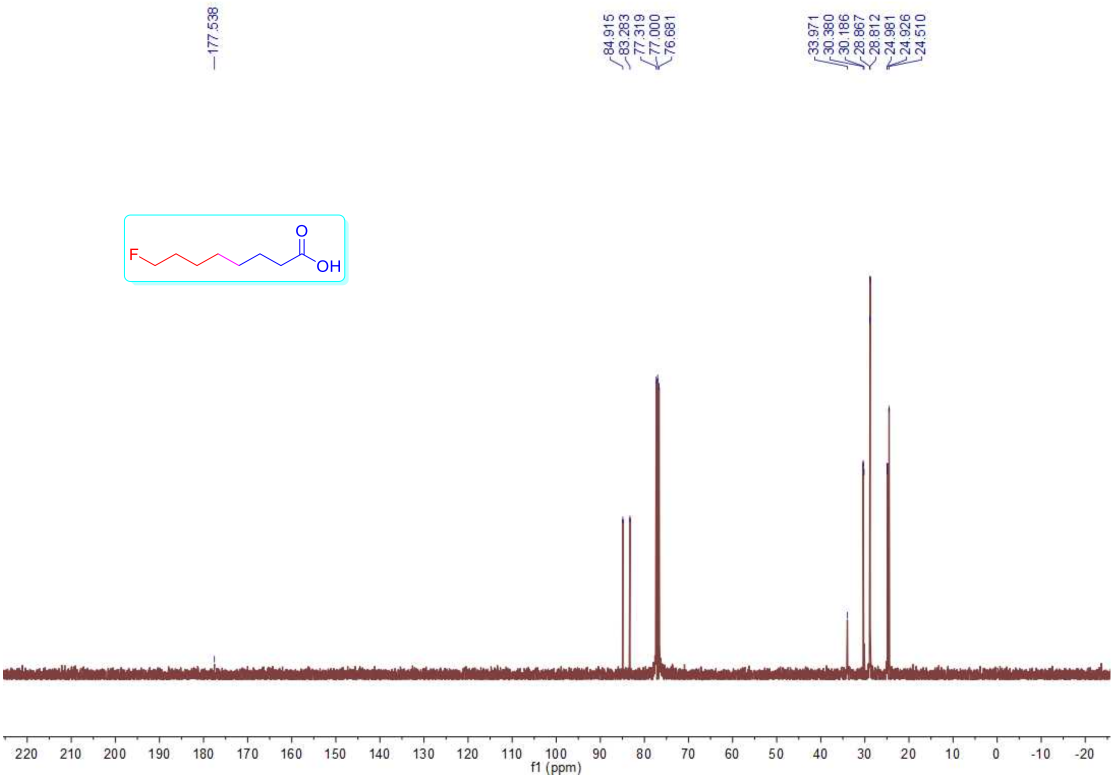

Supplementary Figure 35. ^{19}F NMR Spectrum of 4bi (376 MHz, $CDCl_3$)

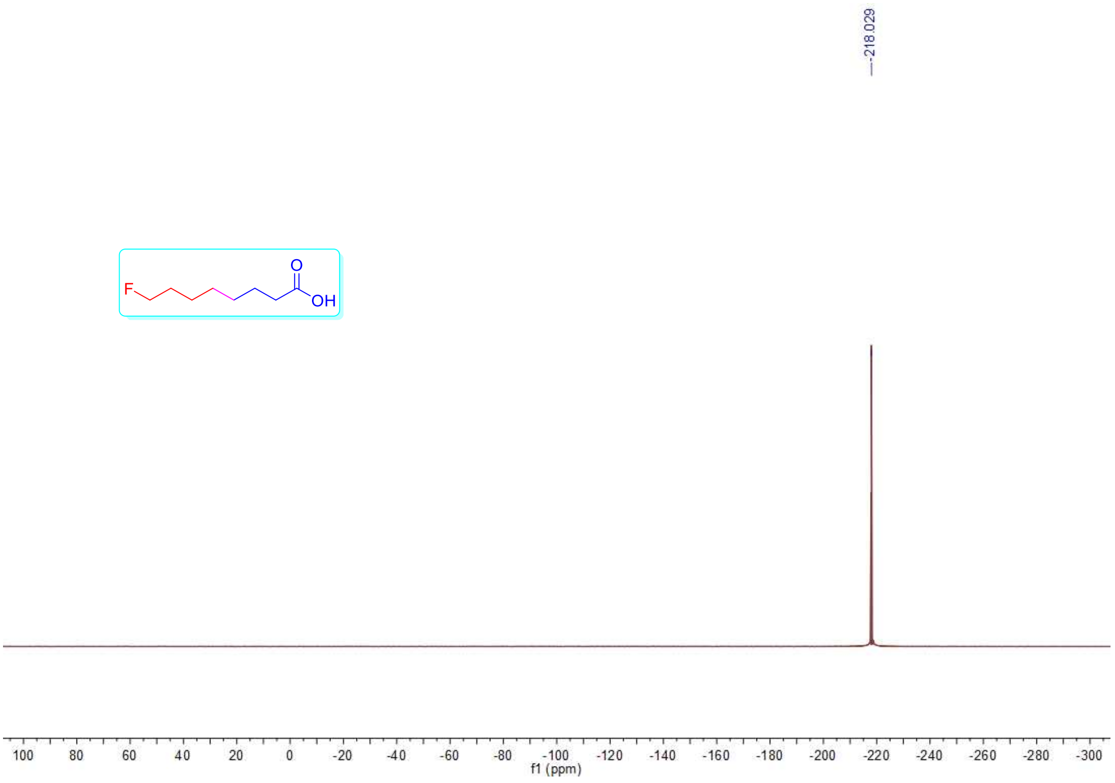

Supplementary Figure 36. ^{1}H NMR Spectrum of 4bj (400 MHz, $CDCl_3$)

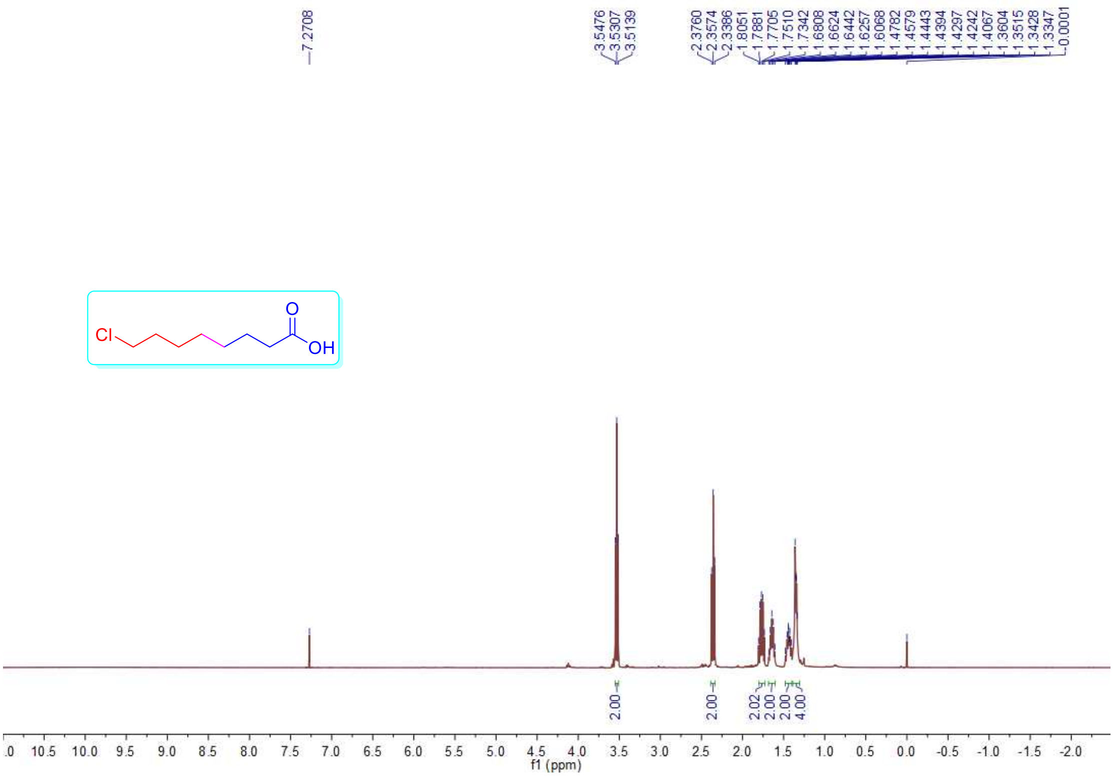

Supplementary Figure 37. ^{13}C NMR Spectrum of 4bj (101 MHz, $CDCl_3$)

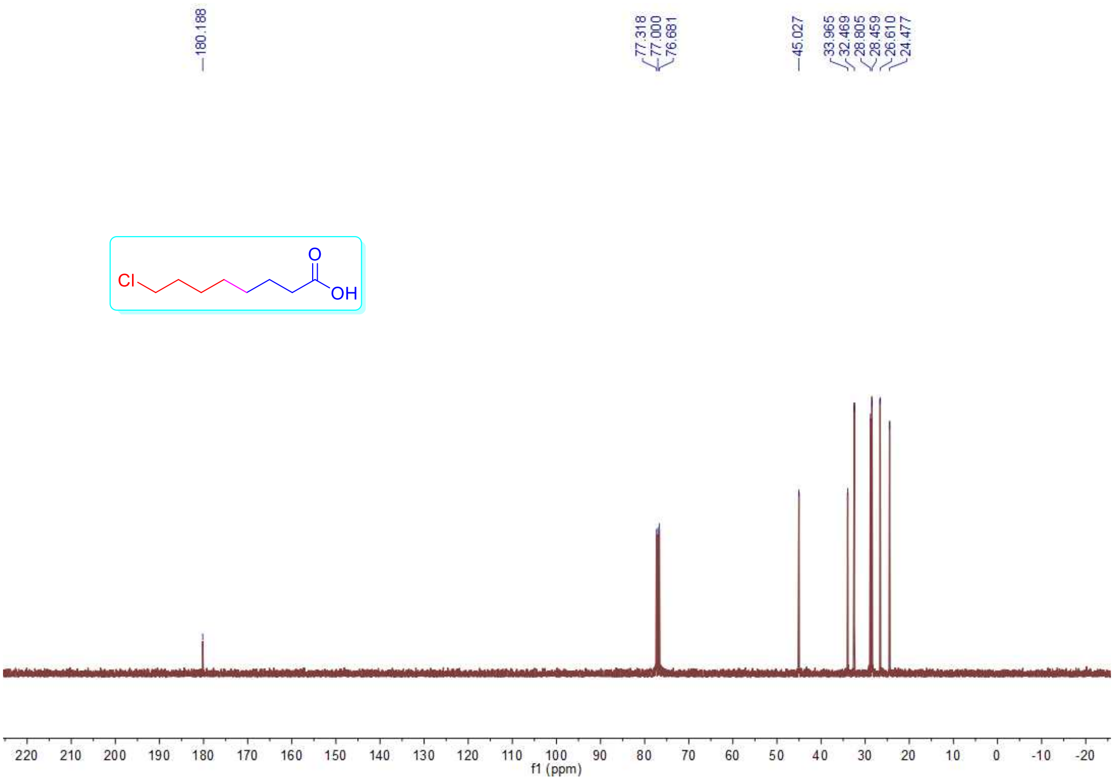

Supplementary Figure 38. ^{1}H NMR Spectrum of 4bk (400 MHz, $CDCl_3$)

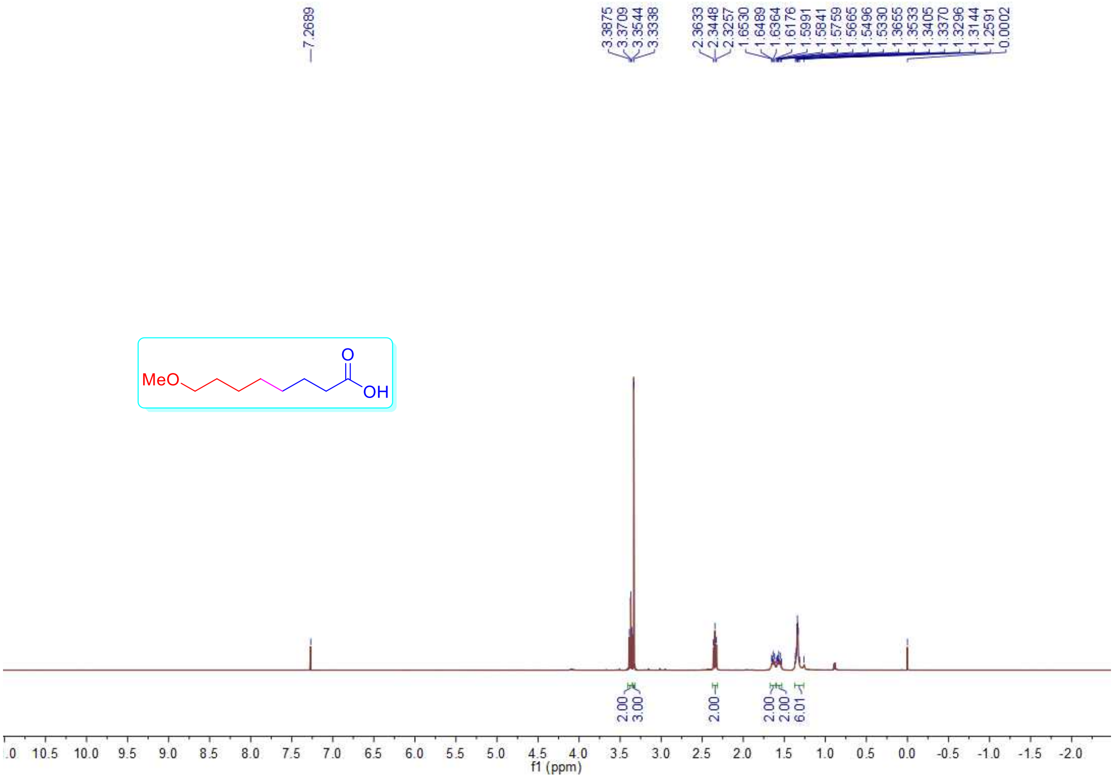

Supplementary Figure 39. ^{13}C NMR Spectrum of 4bk (101 MHz, $CDCl_3$)

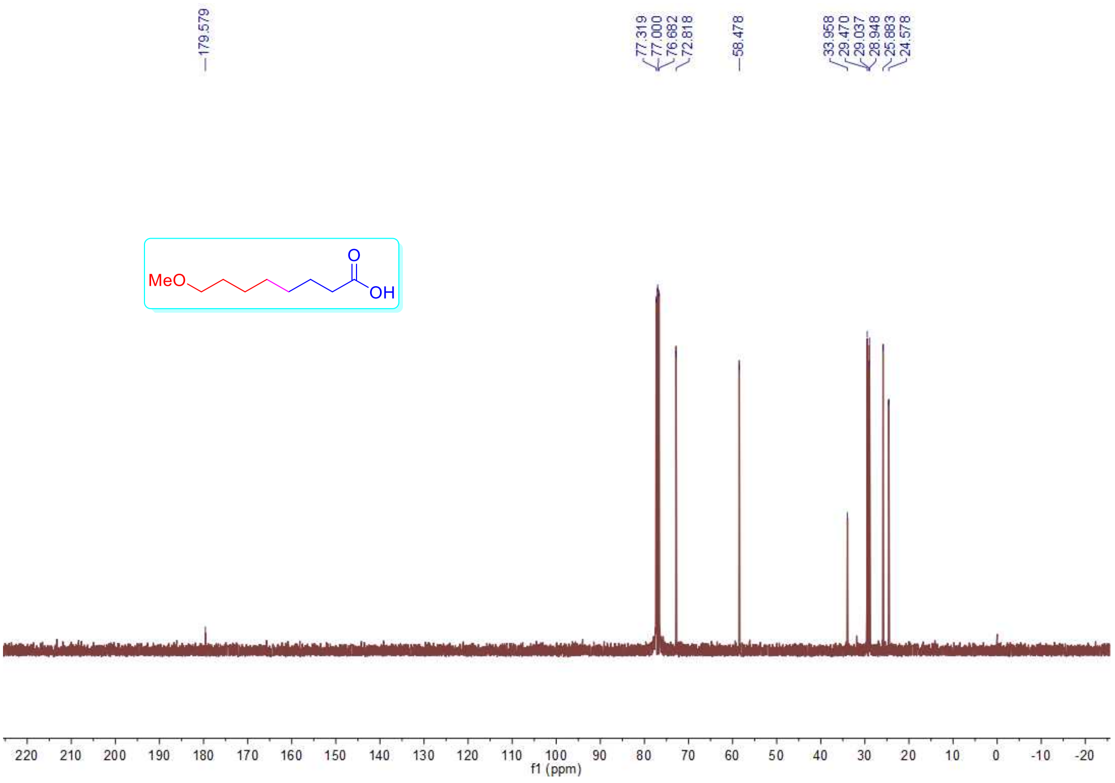

Supplementary Figure 40. ^{1}H NMR Spectrum of 4bl (400 MHz, $CDCl_3$)

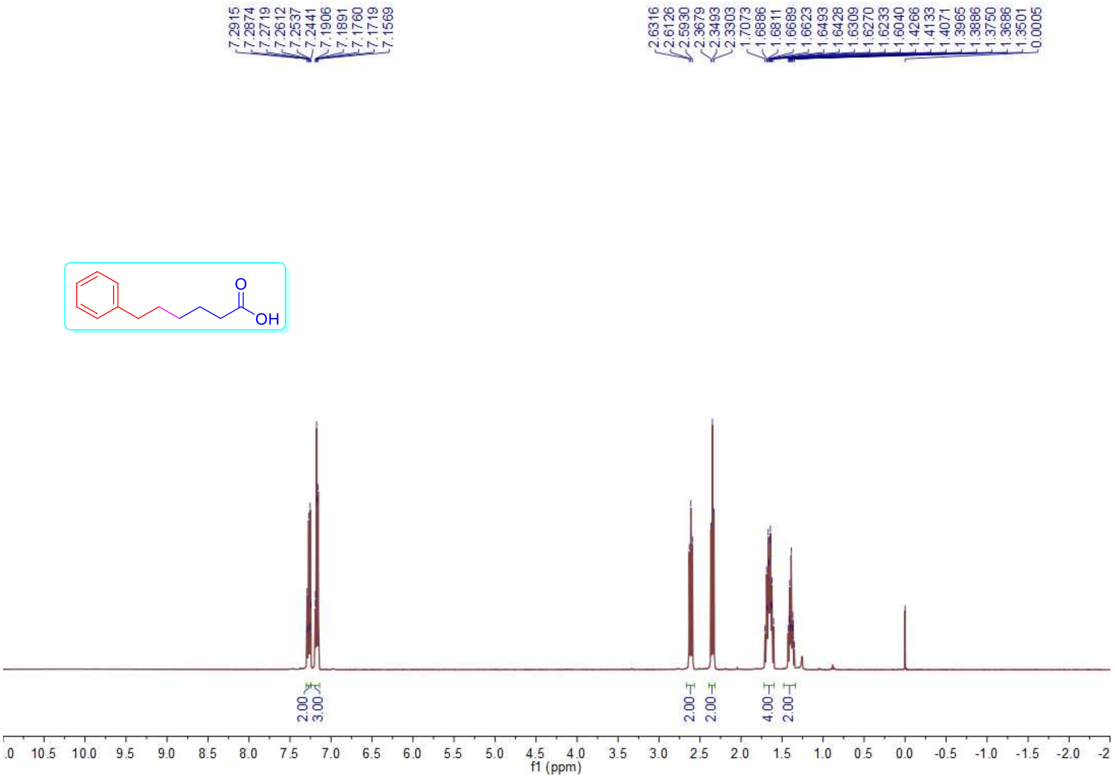

Supplementary Figure 41. ^{13}C NMR Spectrum of 4bl (101 MHz, $CDCl_3$)

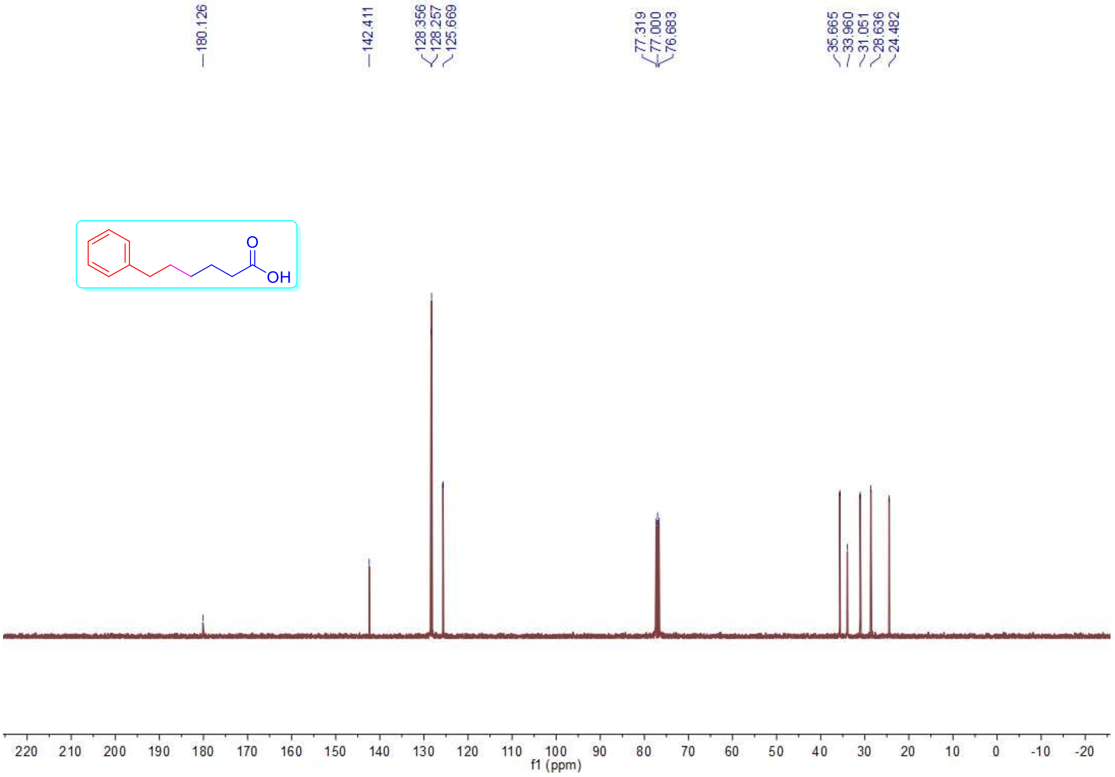

Supplementary Figure 42. ^{1}H NMR Spectrum of 4bm (400 MHz, $CDCl_3$)

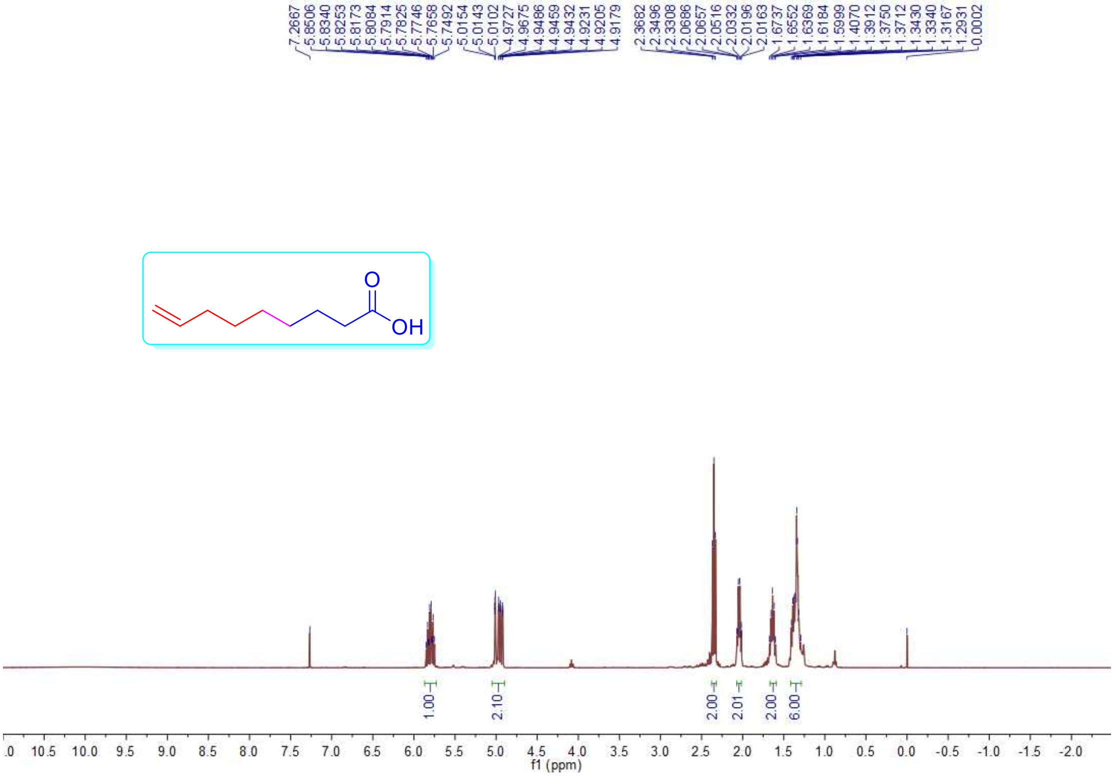

Supplementary Figure 43. ^{13}C NMR Spectrum of 4bm (101 MHz, $CDCl_3$)

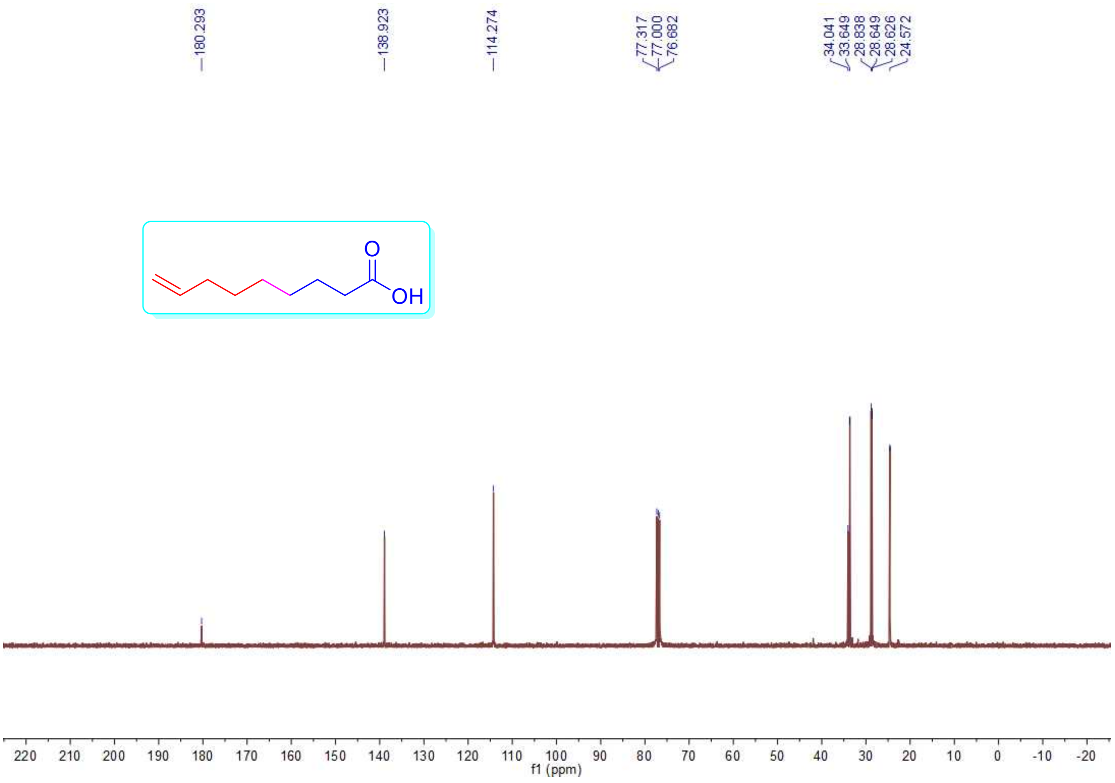

Supplementary Figure 44. ^{1}H NMR Spectrum of 4bp (400 MHz, $CDCl_3$)

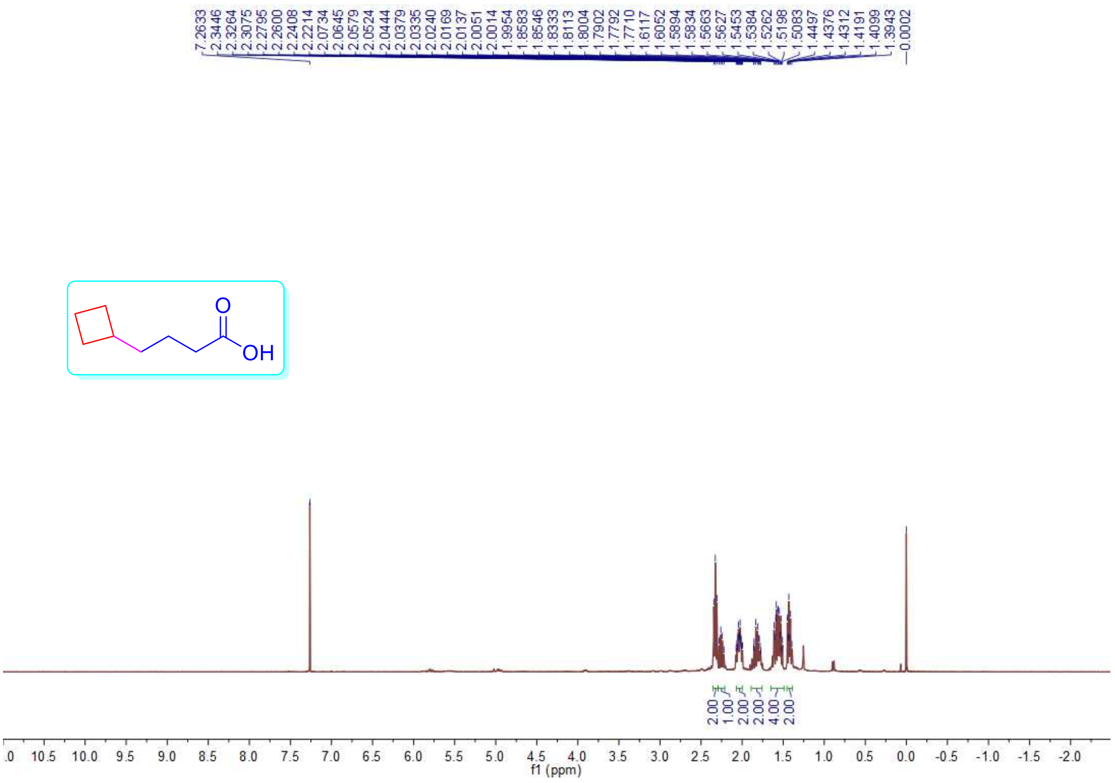

Supplementary Figure 45. ^{13}C NMR Spectrum of 4bp (101 MHz, $CDCl_3$)

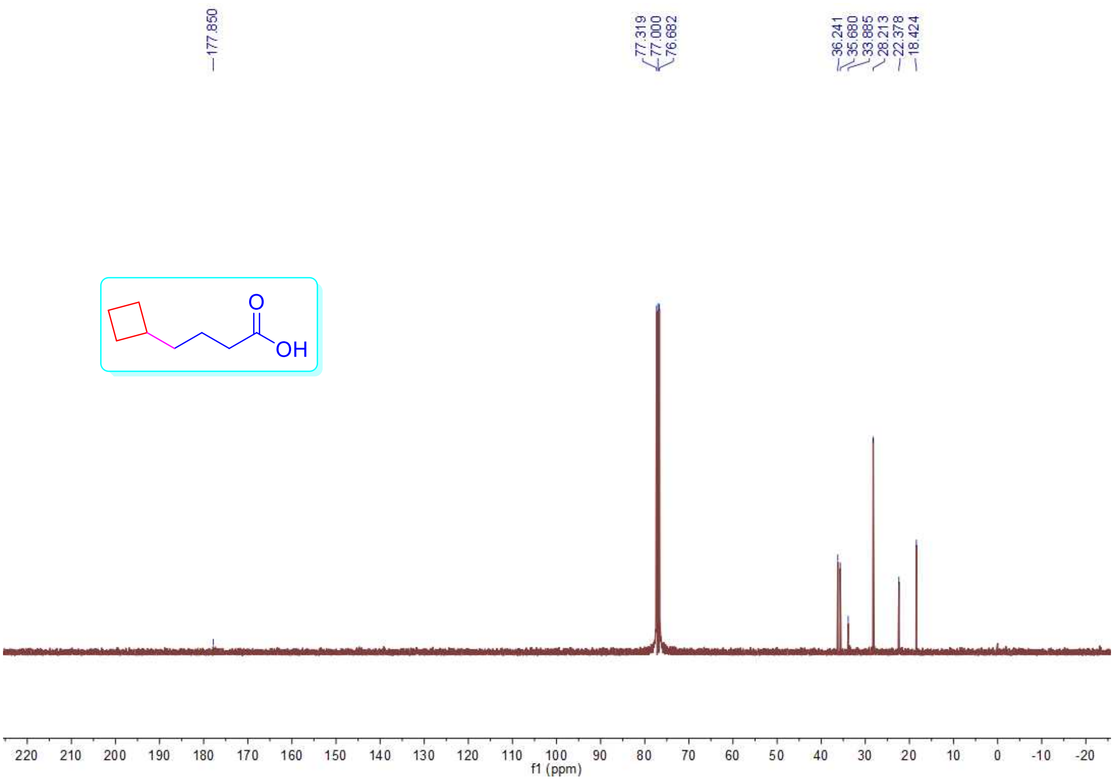

Supplementary Figure 46. ^{1}H NMR Spectrum of 4bn (400 MHz, $CDCl_3$)

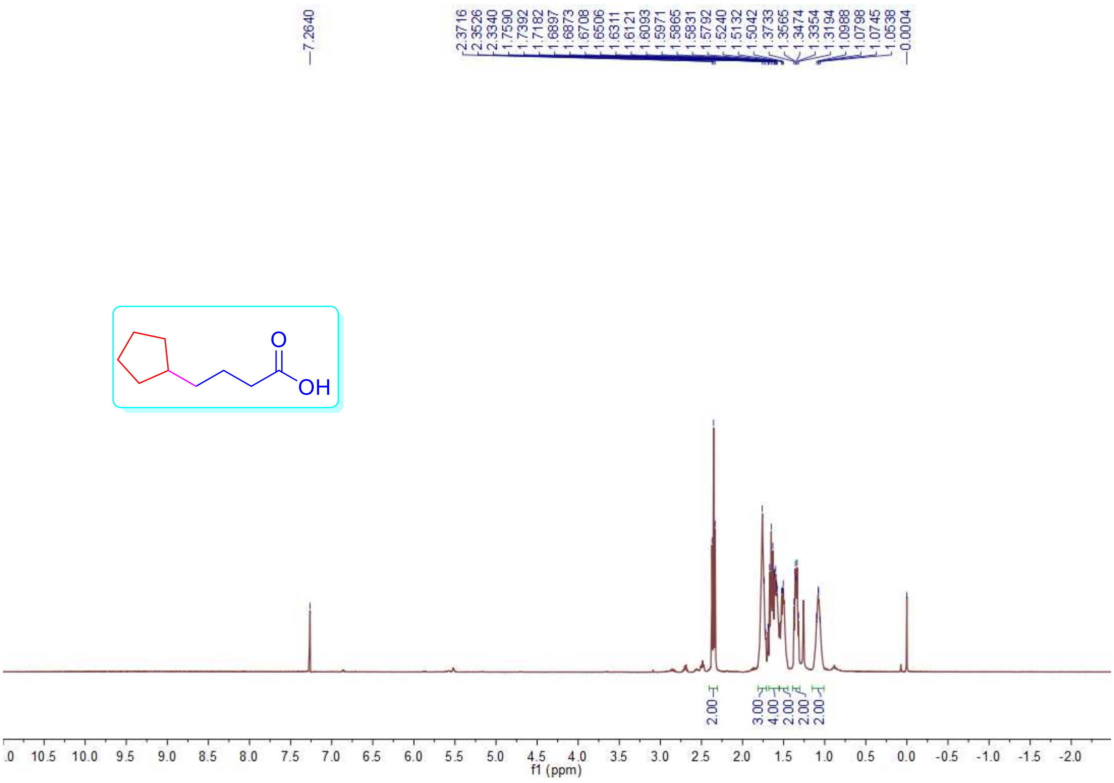

Supplementary Figure 47. ^{13}C **NMR Spectrum of 4bn (101 MHz,** $CDCl_3$**)**

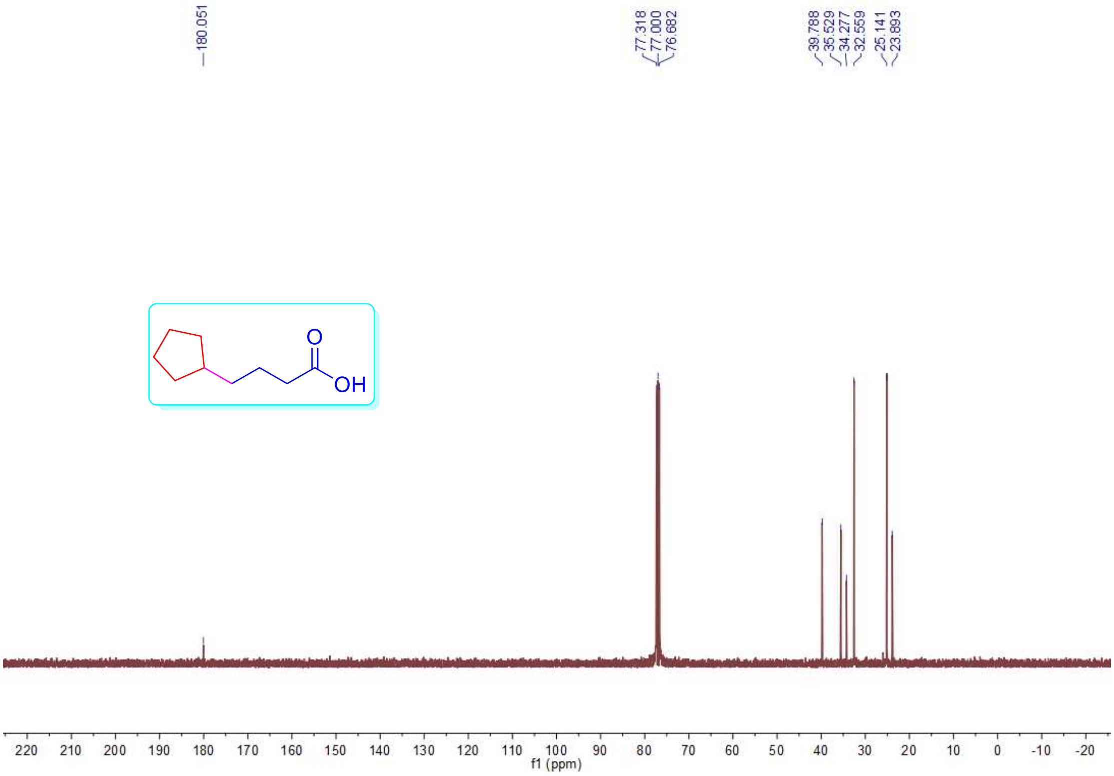

Supplementary Figure 48. ^{1}H **NMR Spectrum of 4bo (400 MHz,** $CDCl_3$**)**

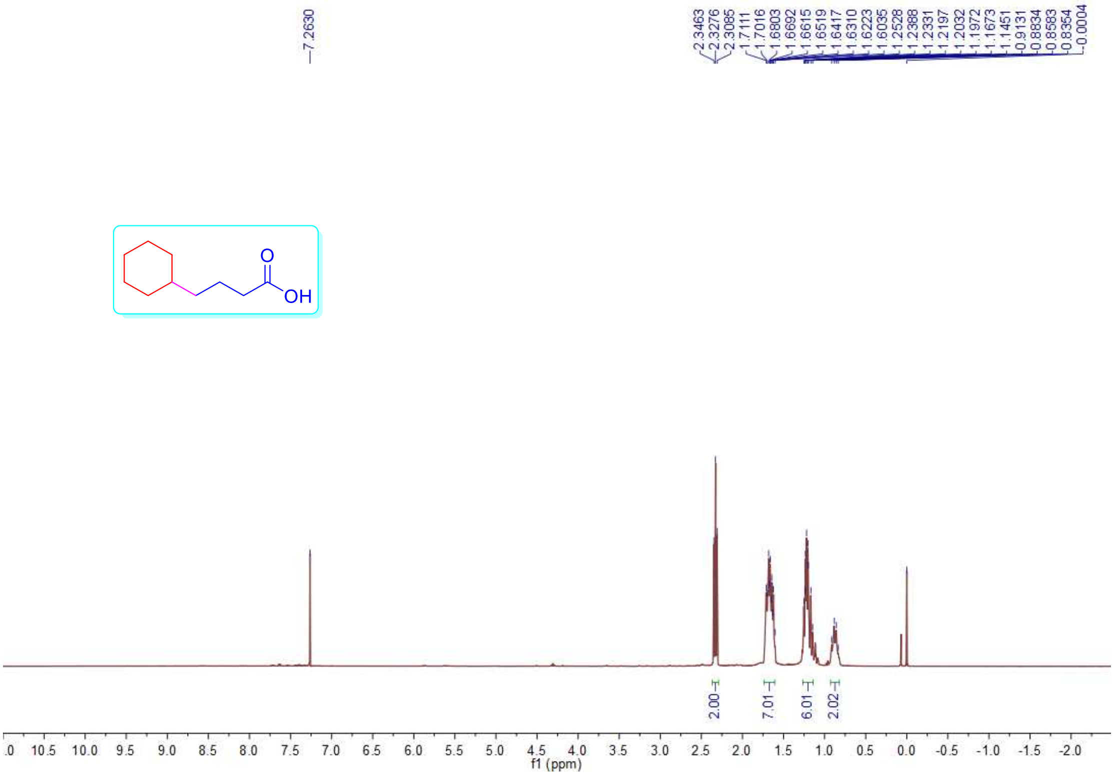

Supplementary Figure 49. ^{13}C NMR Spectrum of 4bo (101 MHz, $CDCl_3$)

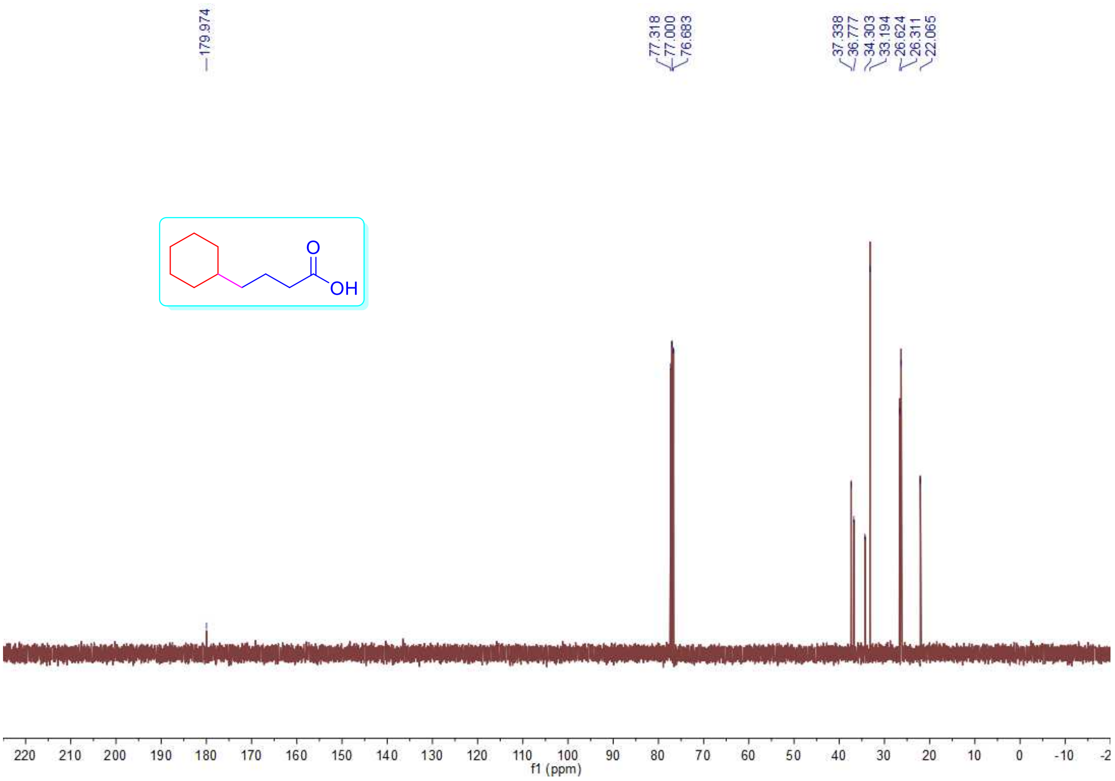

Supplementary Figure 50. ^{1}H NMR Spectrum of 4bq (400 MHz, $CDCl_3$)

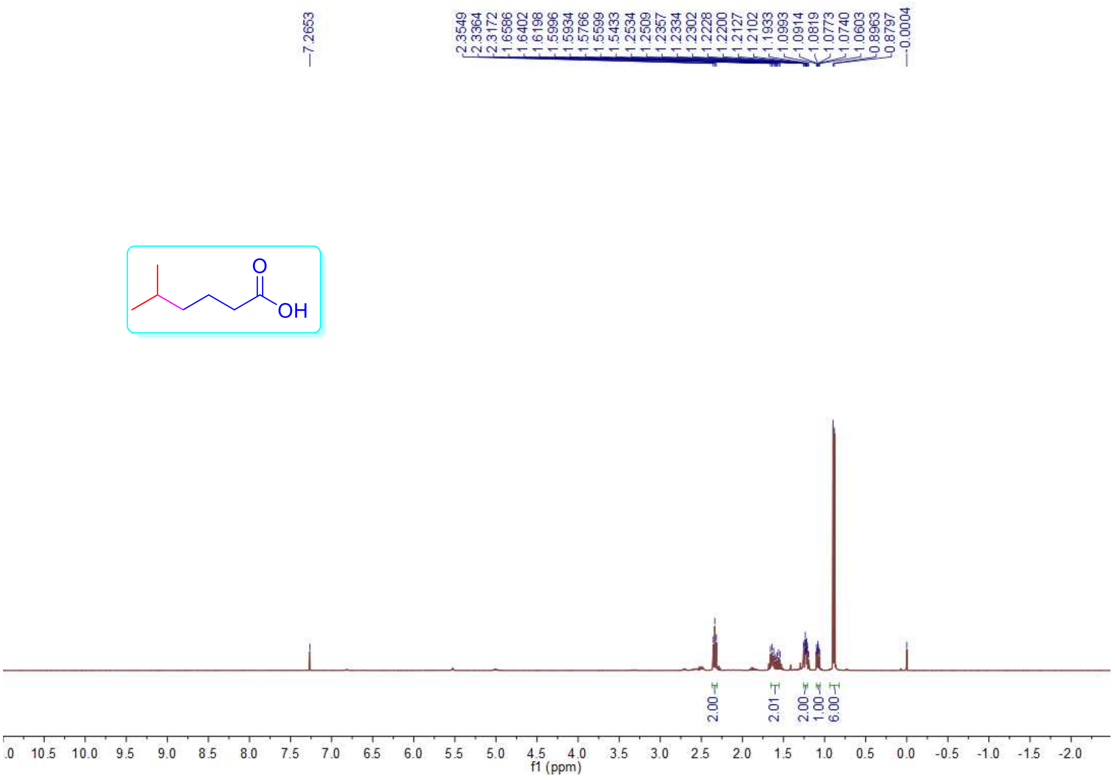

Supplementary Figure 51. ^{13}C NMR Spectrum of 4bq (101 MHz, $CDCl_3$)

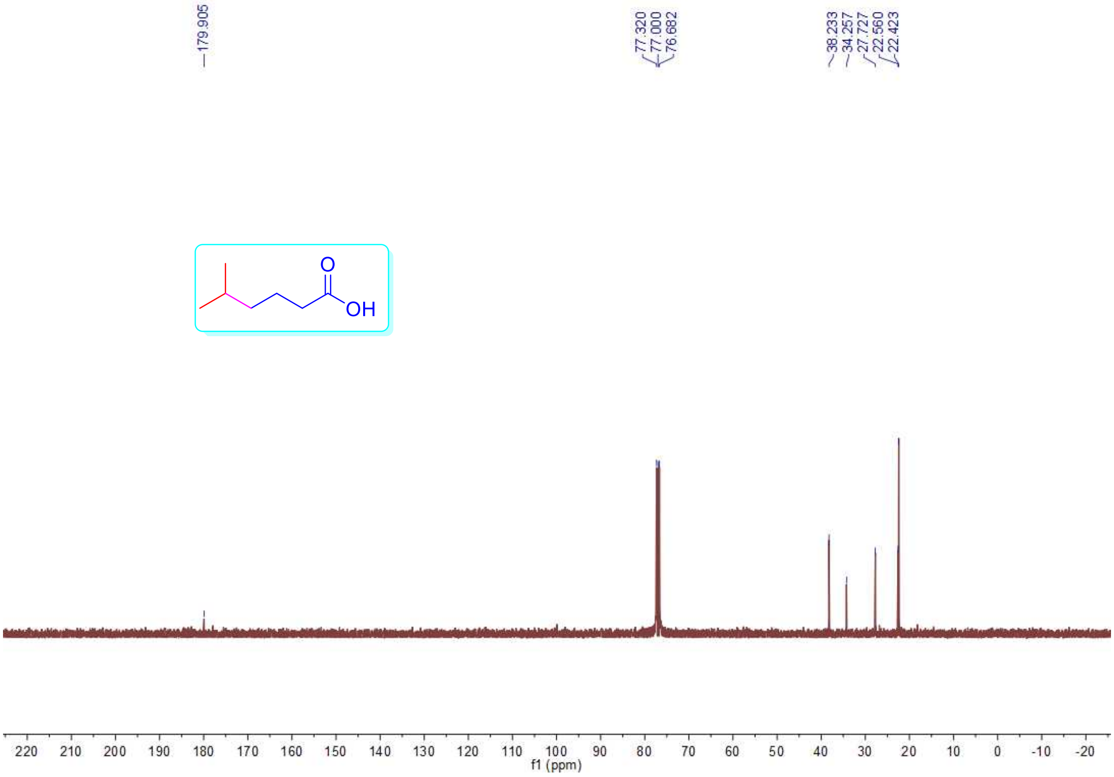

Supplementary Figure 52. ^{1}H NMR Spectrum of 4br (400 MHz, $CDCl_3$)

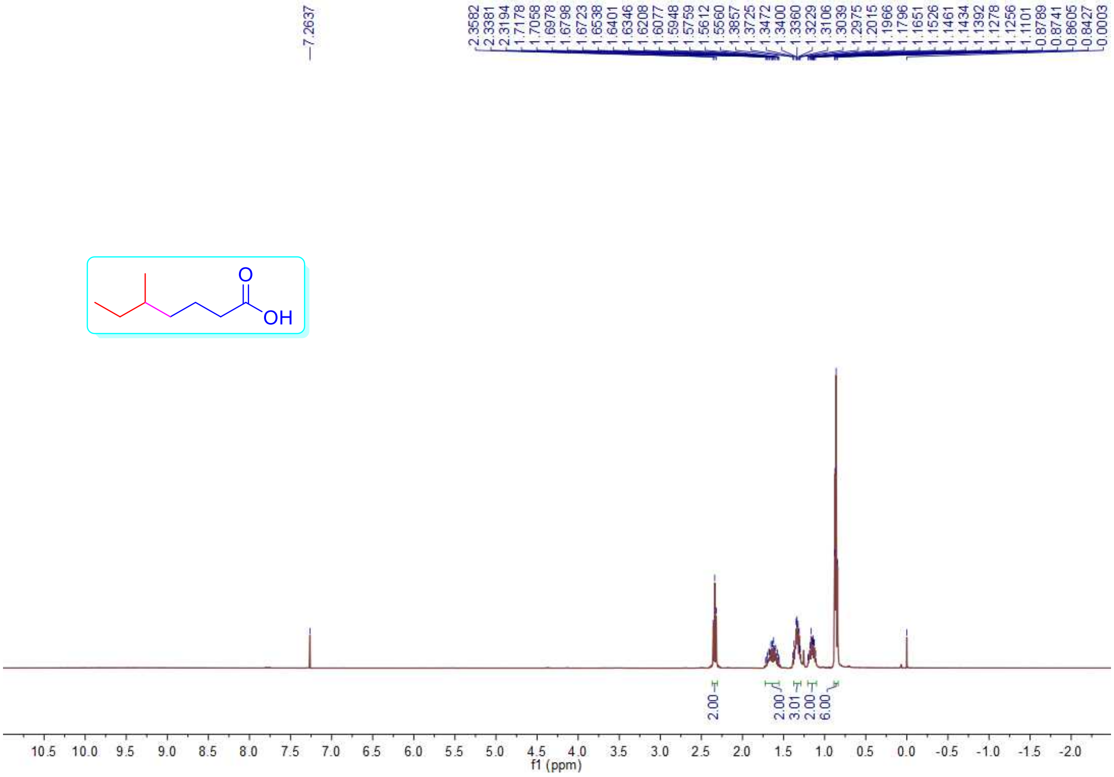

Supplementary Figure 53. ^{13}C NMR Spectrum of 4br (101 MHz, $CDCl_3$)

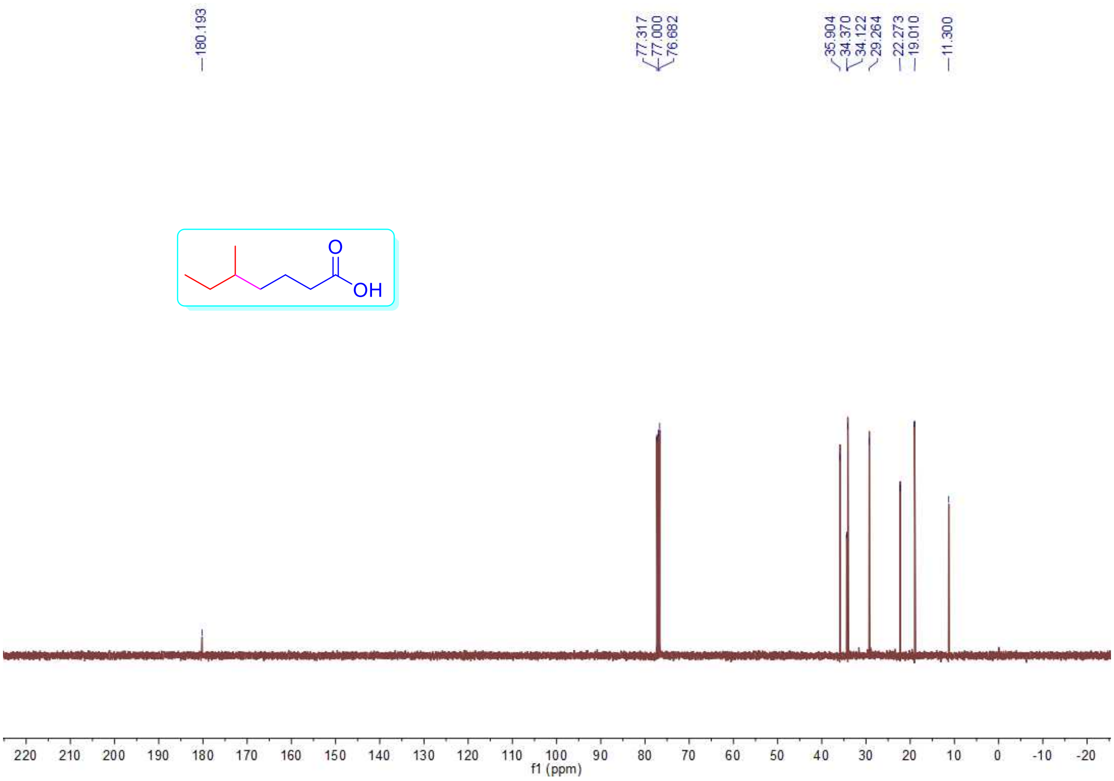

Supplementary Figure 54. ^{1}H NMR Spectrum of 4bs (400 MHz, $CDCl_3$)

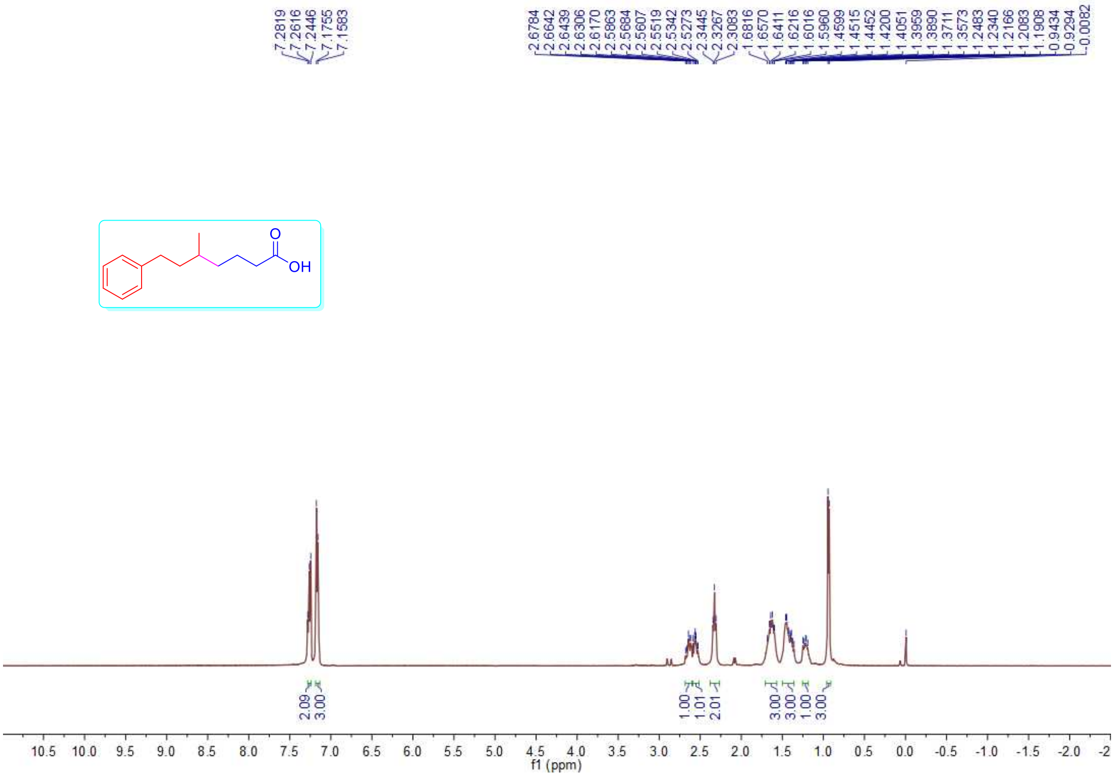

Supplementary Figure 55. ^{13}C **NMR Spectrum of 4bs (101 MHz,** $CDCl_3$**)**

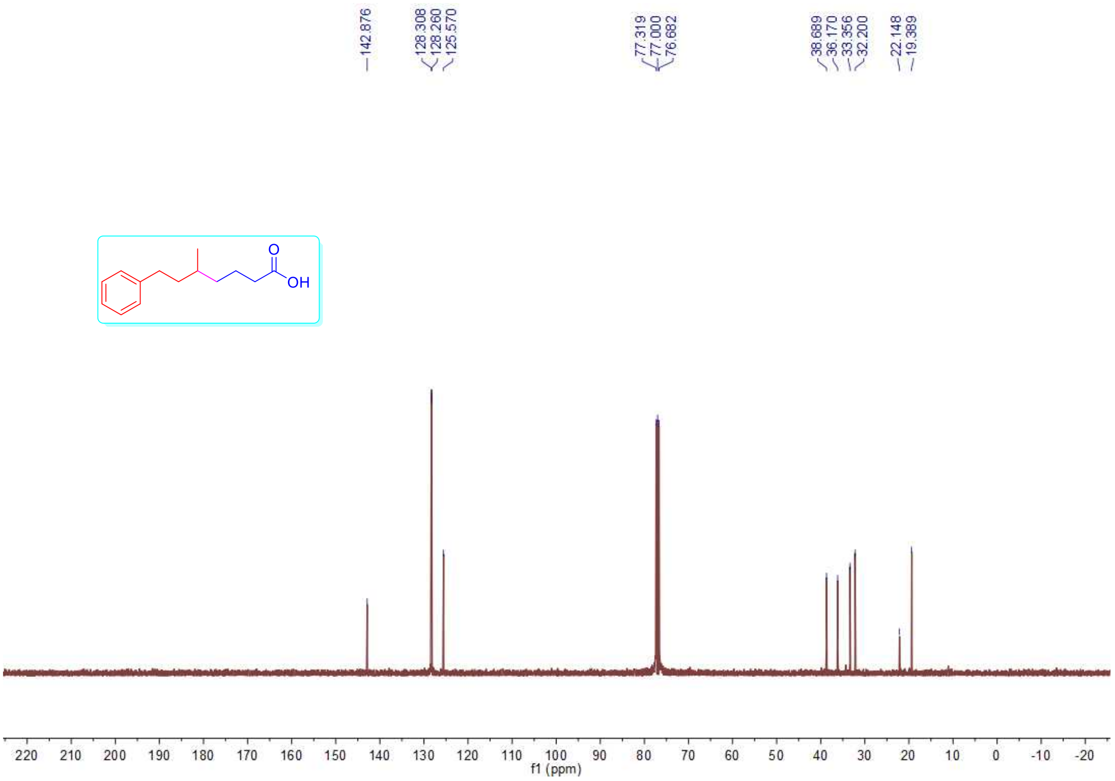

Supplementary Figure 56. ^{1}H **NMR Spectrum of 4bt (400 MHz,** $CDCl_3$**)**

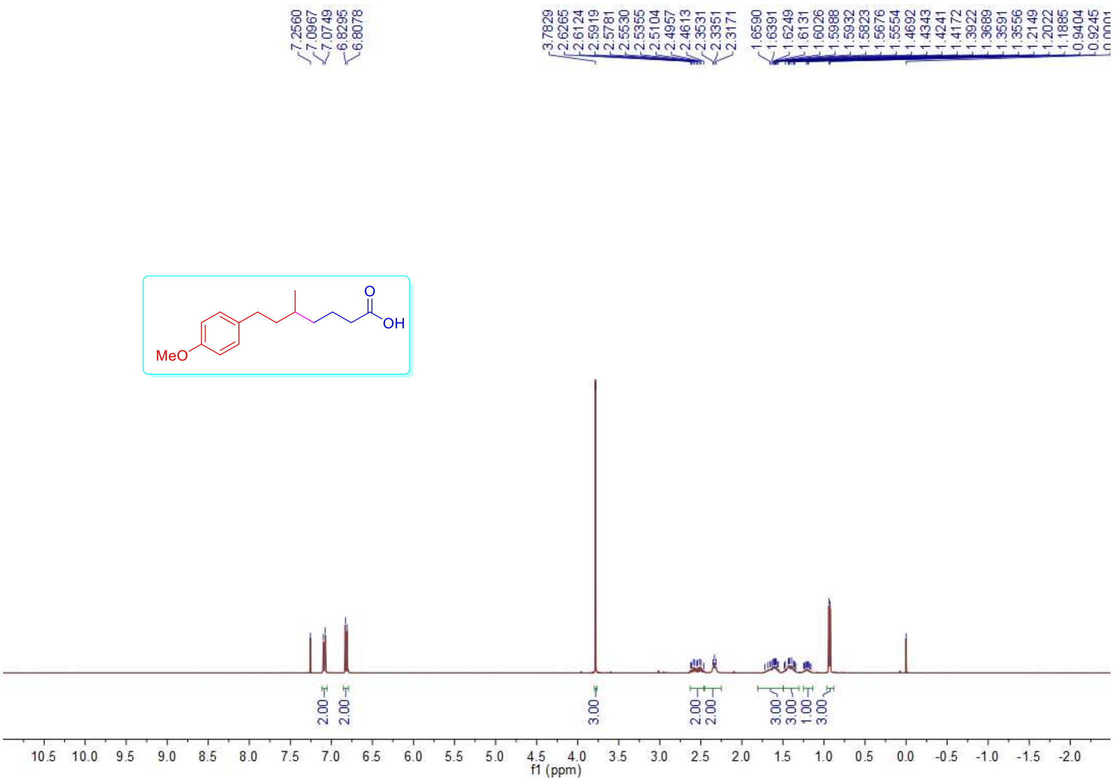

Supplementary Figure 57. ^{13}**C NMR Spectrum of 4bt (101 MHz,** $CDCl_3$**)**

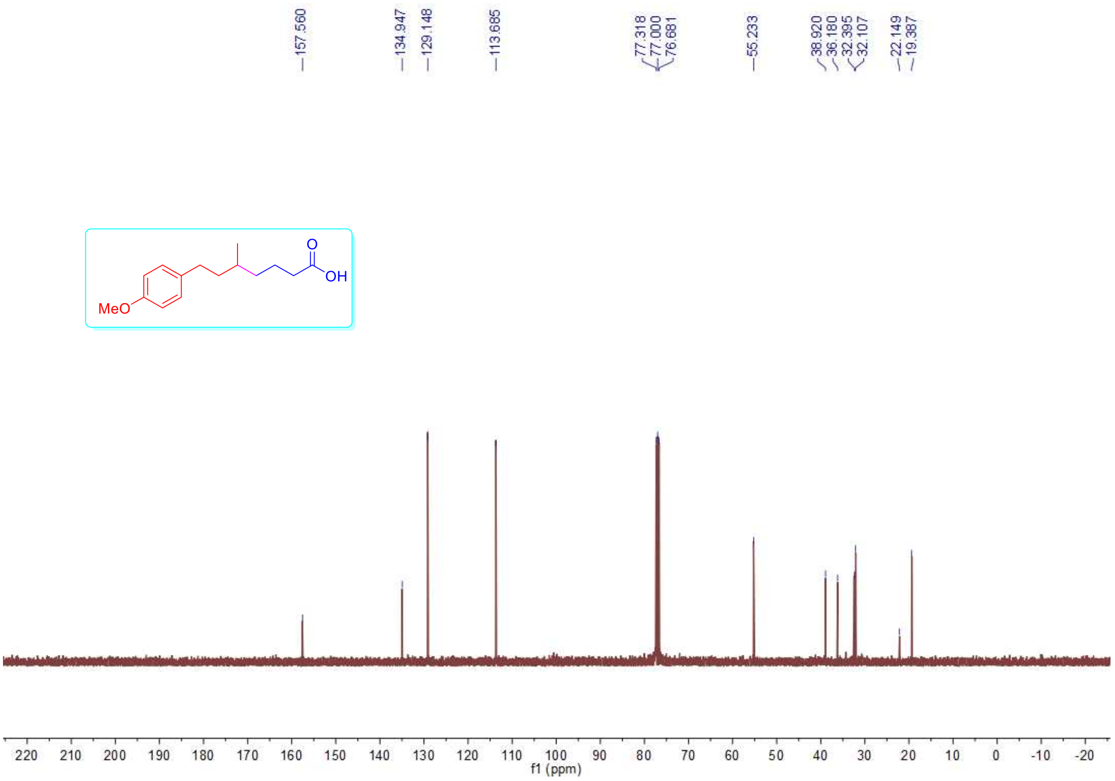

Supplementary Figure 58. ^{1}**H NMR Spectrum of 5cl (400 MHz,** $CDCl_3$**)**

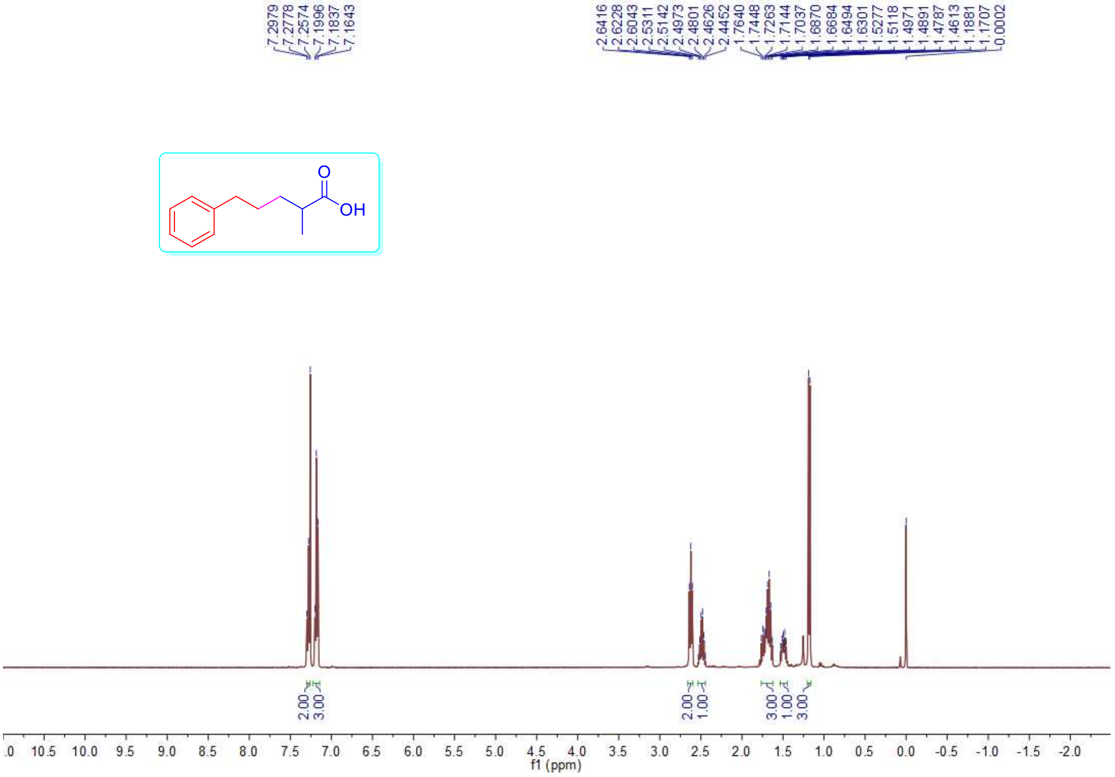

Supplementary Figure 59. ^{13}C **NMR Spectrum of 5cl (101 MHz,** $CDCl_3$**)**

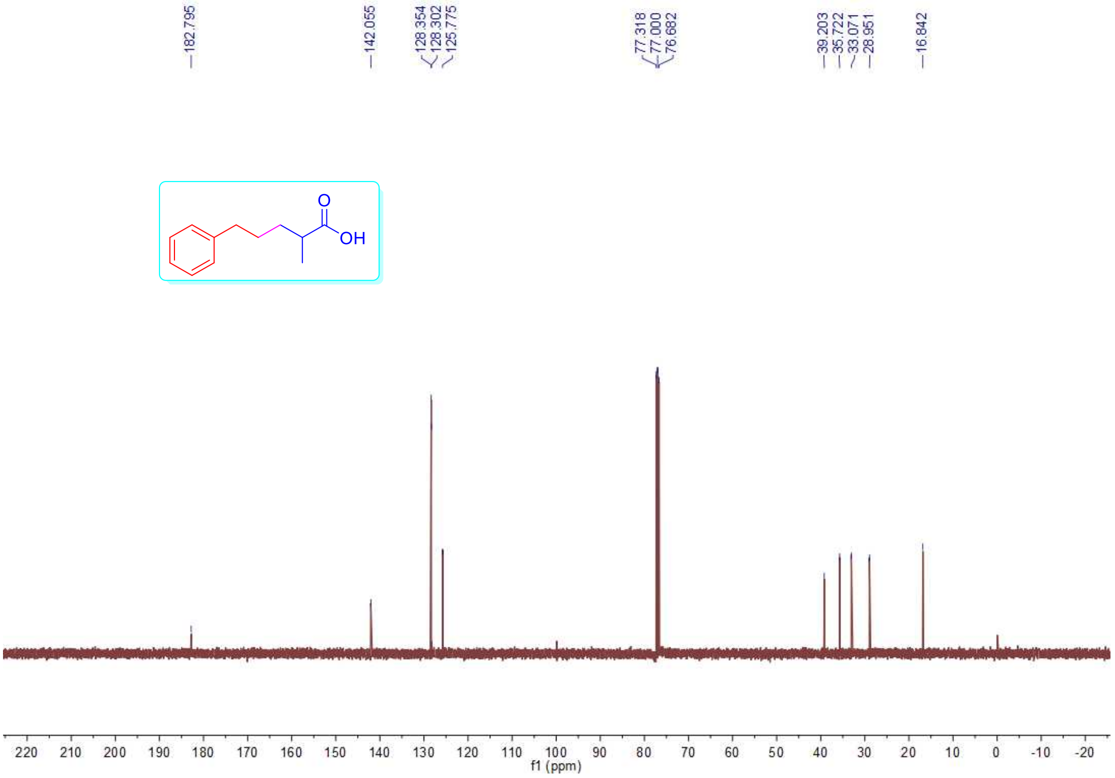

Supplementary Figure 60. ^{1}H **NMR Spectrum of 5cf (400 MHz,** $CDCl_3$**)**

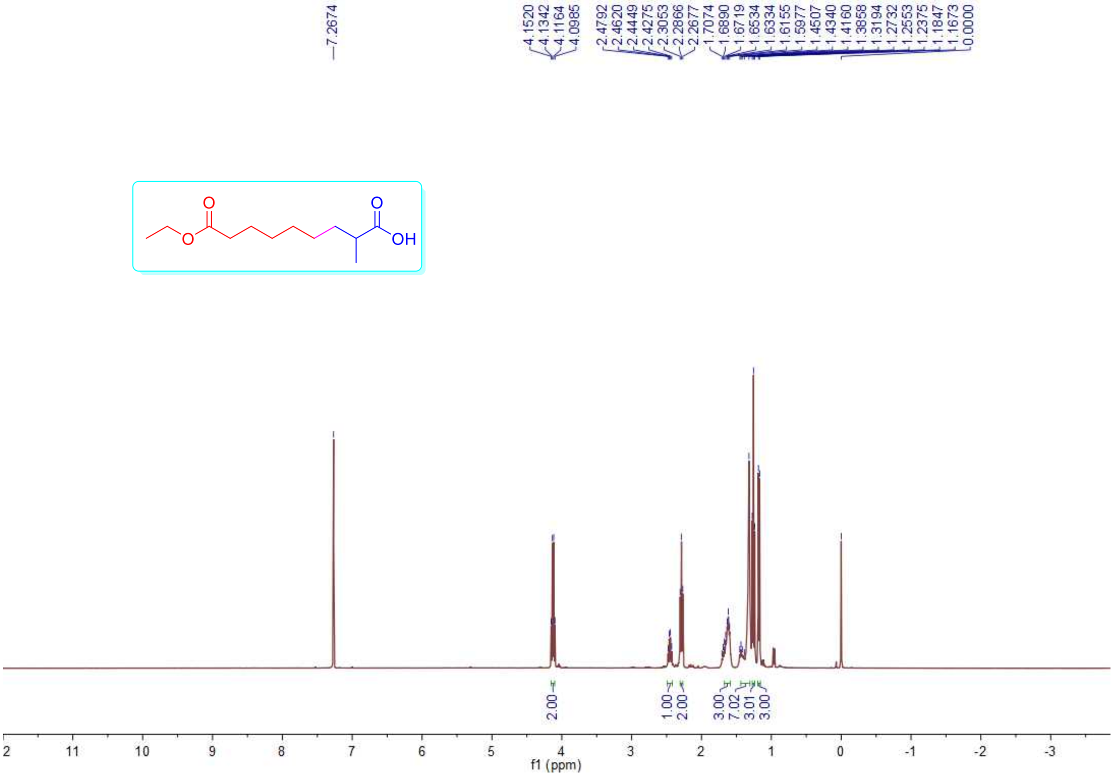

Supplementary Figure 61. ^{13}C NMR Spectrum of 5cf (101 MHz, $CDCl_3$)

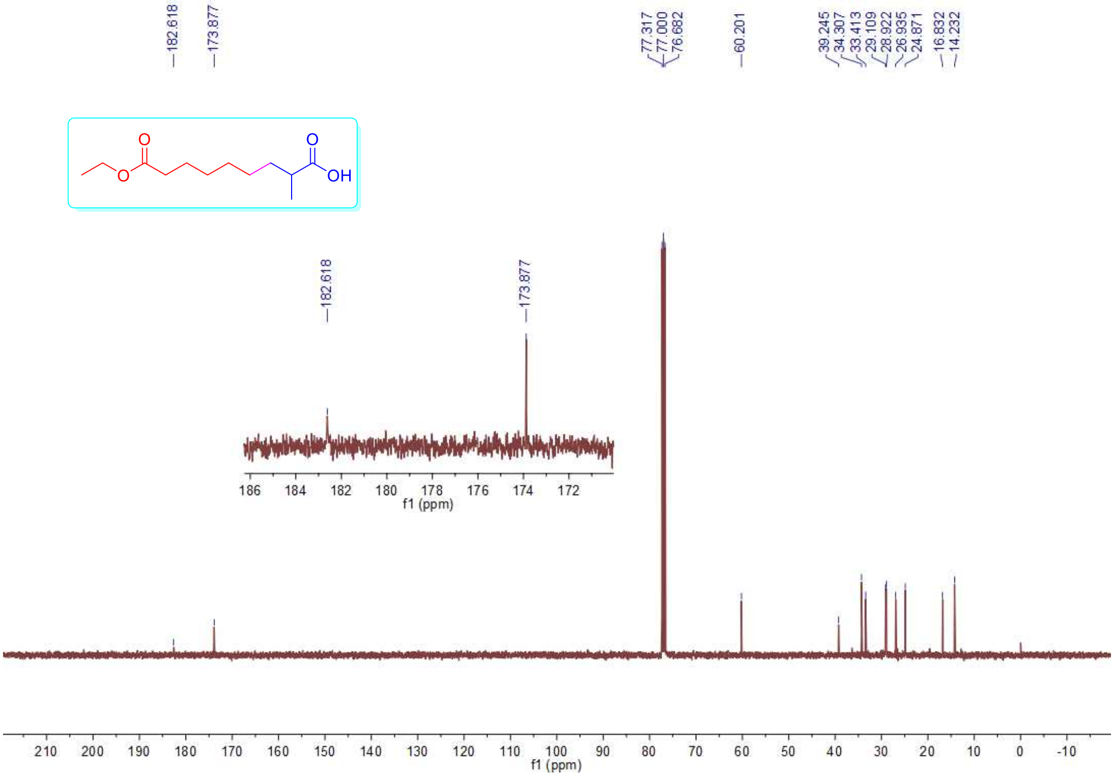

Supplementary Figure 62. ^{1}H NMR Spectrum of 6dl (400 MHz, $CDCl_3$)

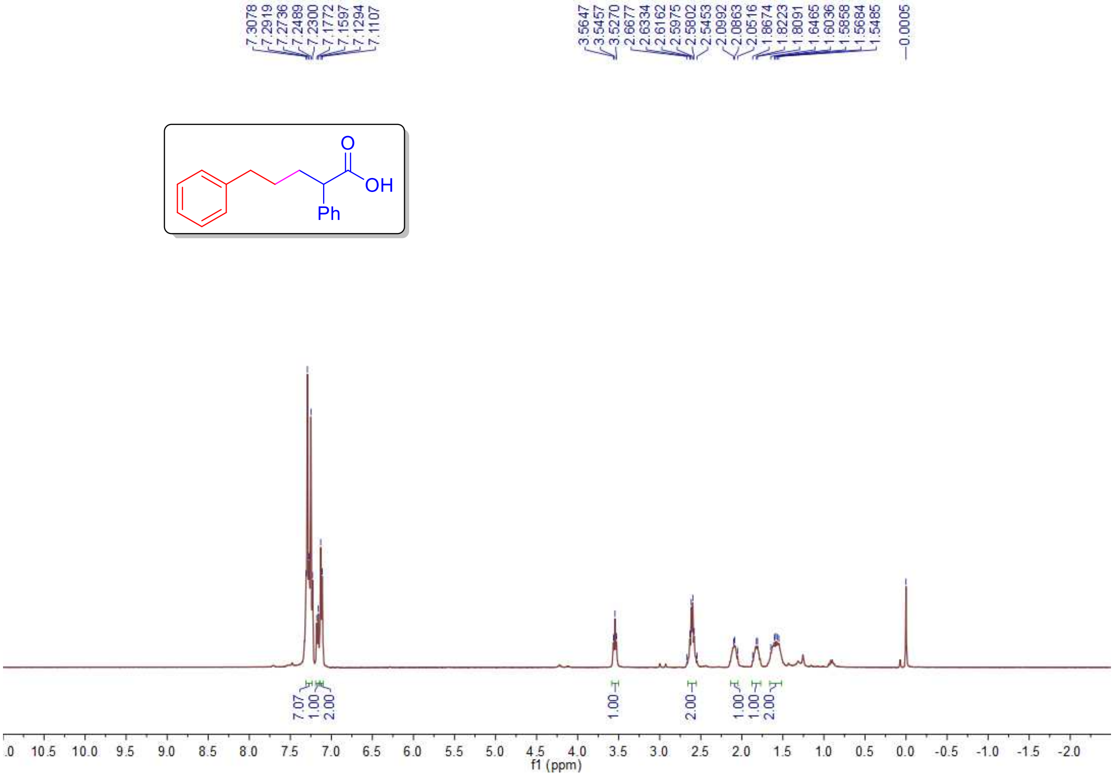

Supplementary Figure 63. ^{13}C NMR Spectrum of 6dl (101 MHz, $CDCl_3$)

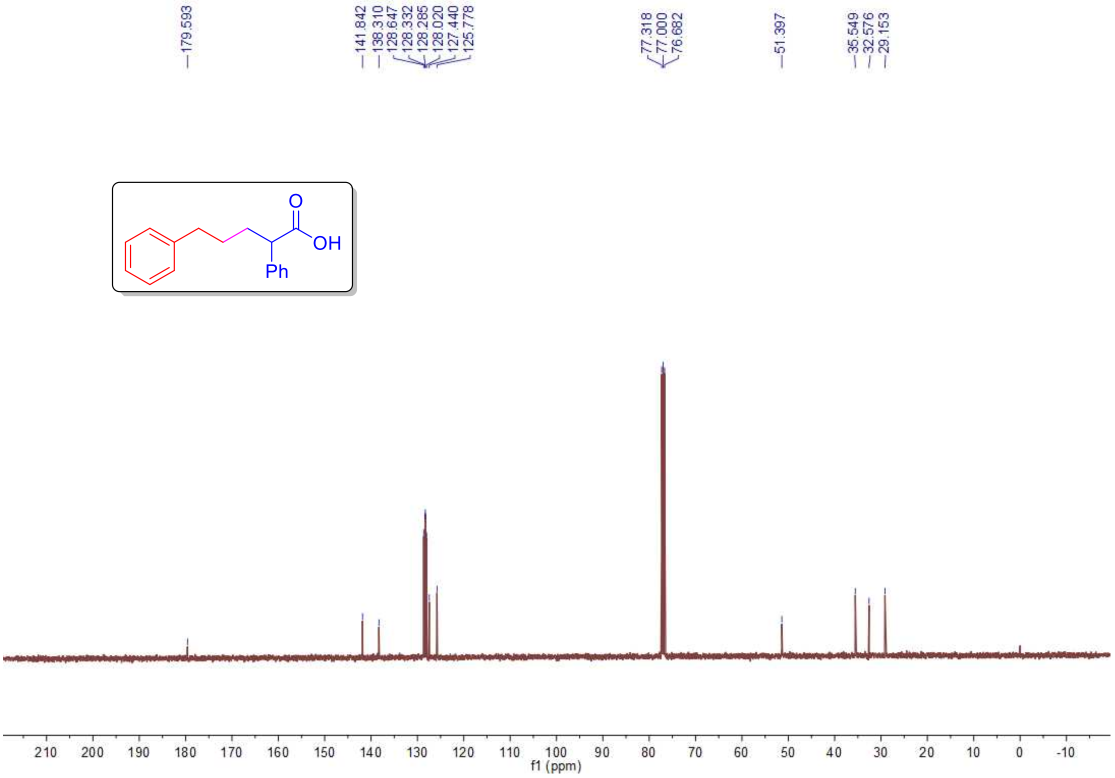

Supplementary Figure 64. ^{1}H NMR Spectrum of 6df (400 MHz, $CDCl_3$)

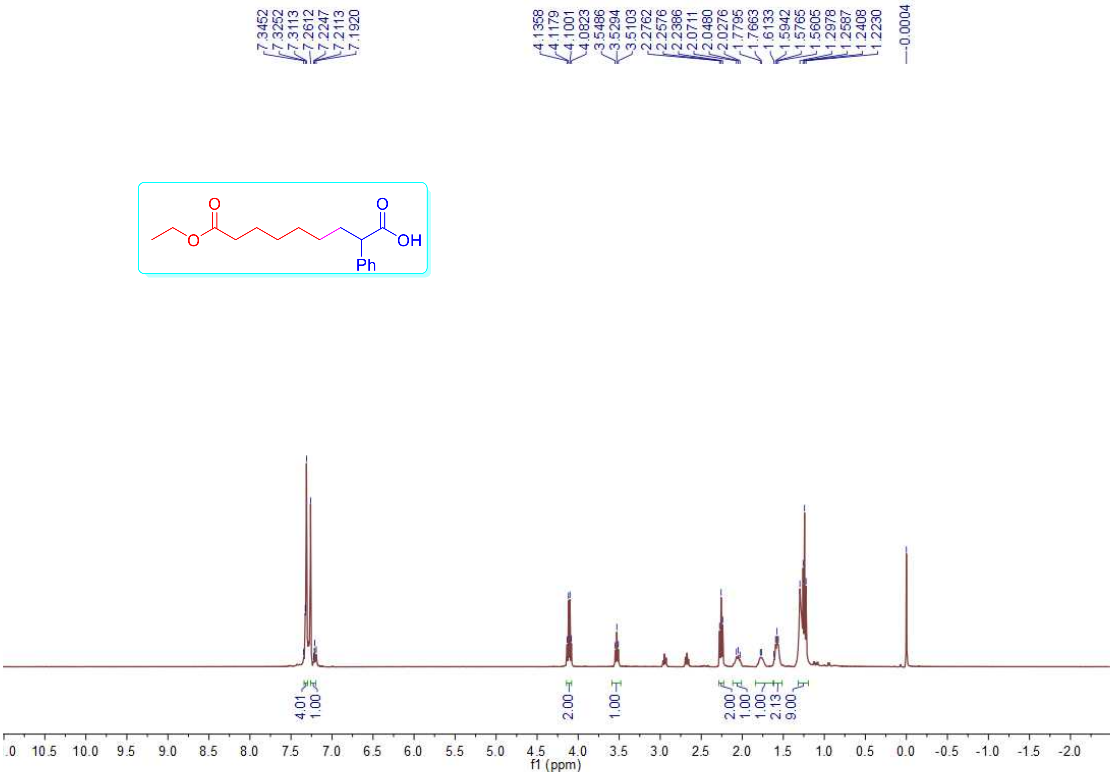

Supplementary Figure 65. ^{13}C NMR Spectrum of 6df (101 MHz, $CDCl_3$)

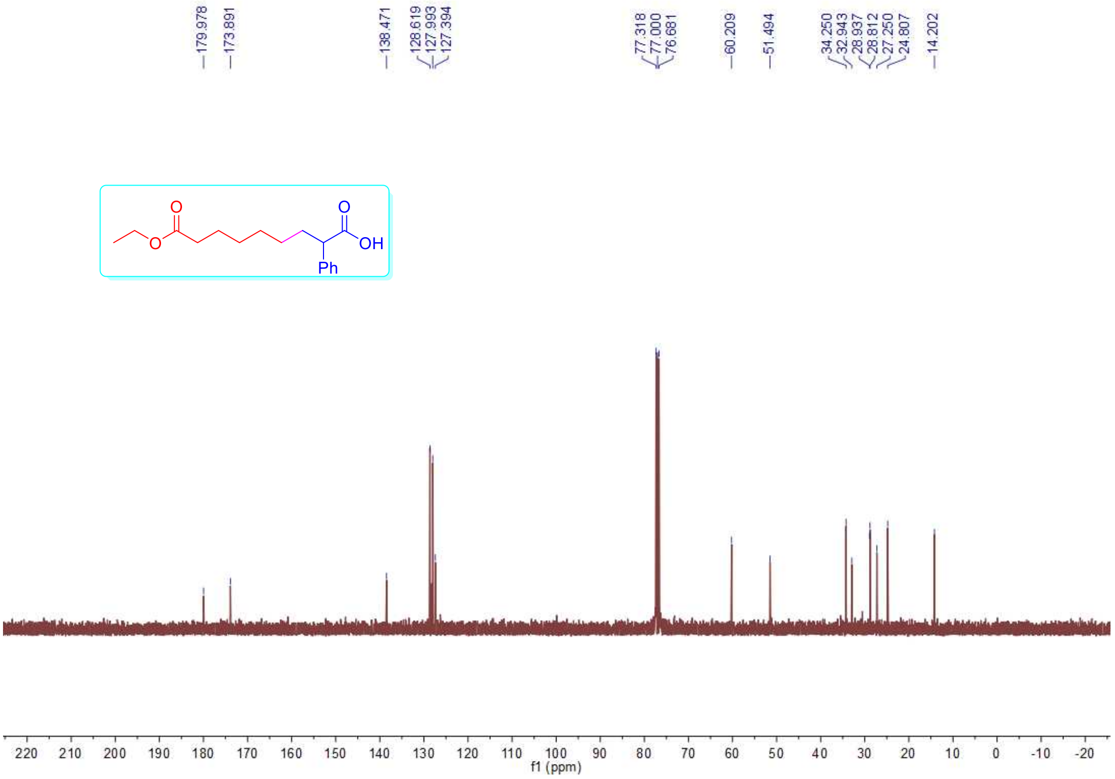

Supplementary Figure 66. ^{1}H NMR Spectrum of 7el (400 MHz, $CDCl_3$)

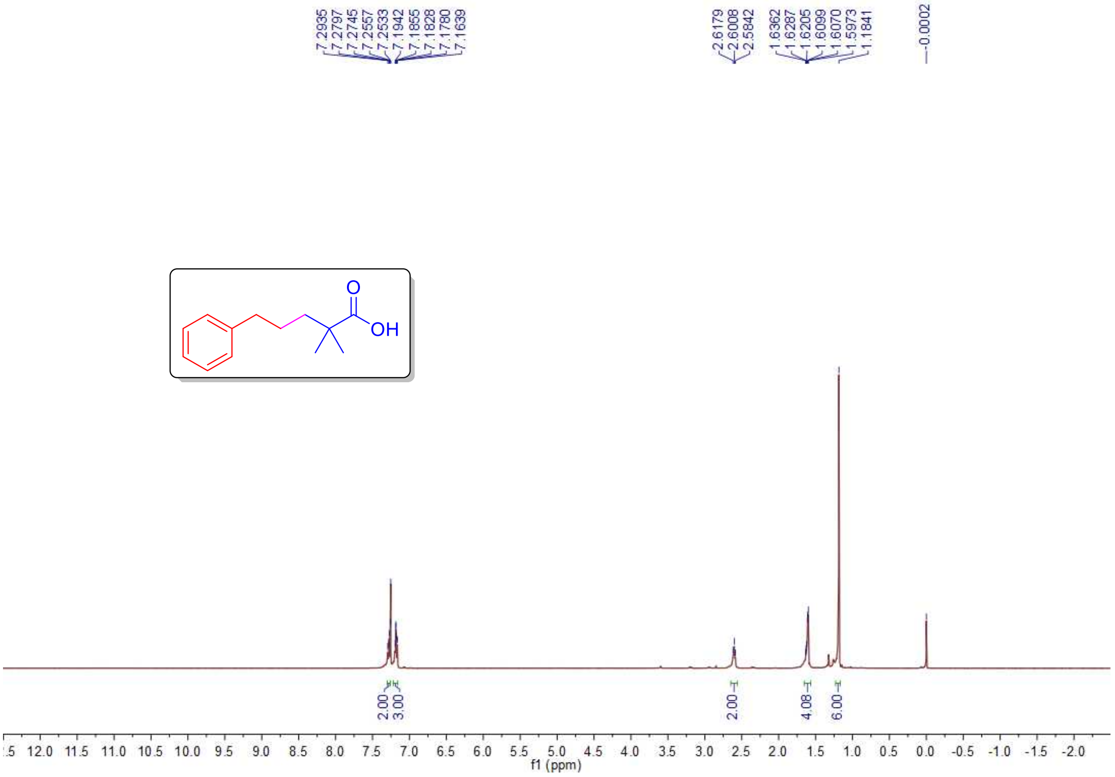

Supplementary Figure 67. ^{13}C NMR Spectrum of 7el (101 MHz, $CDCl_3$)

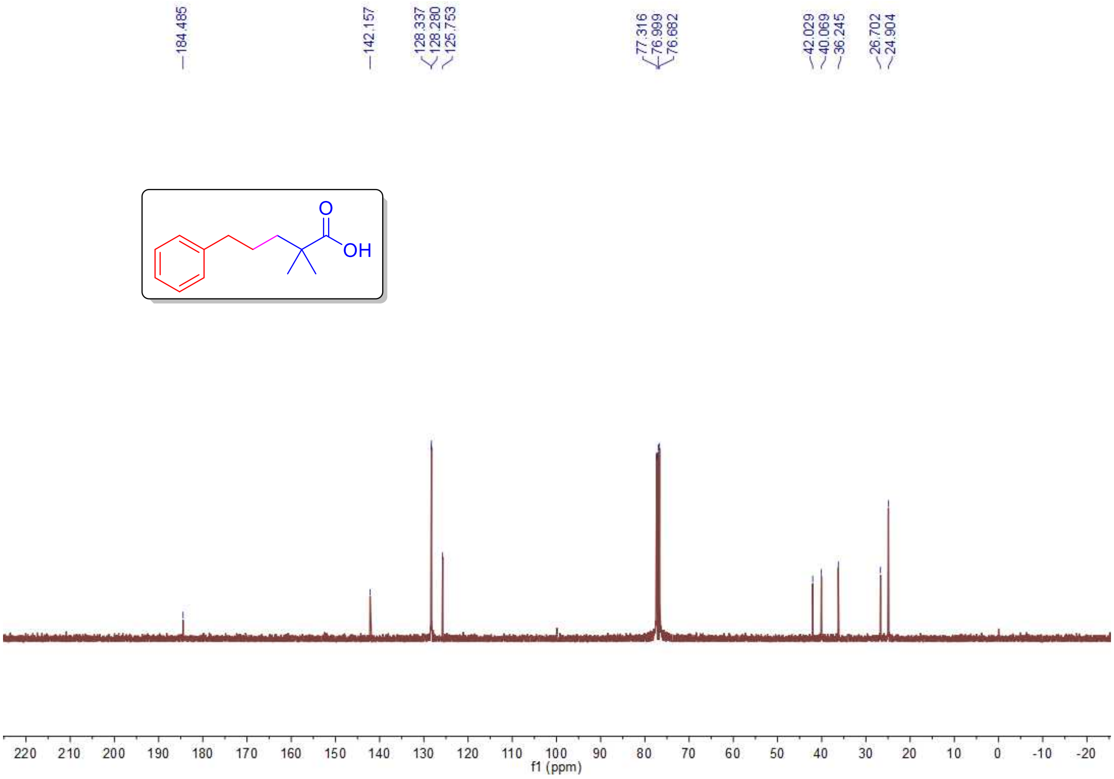

Supplementary Figure 68. ^{1}H NMR Spectrum of 7ef (400 MHz, $CDCl_3$)

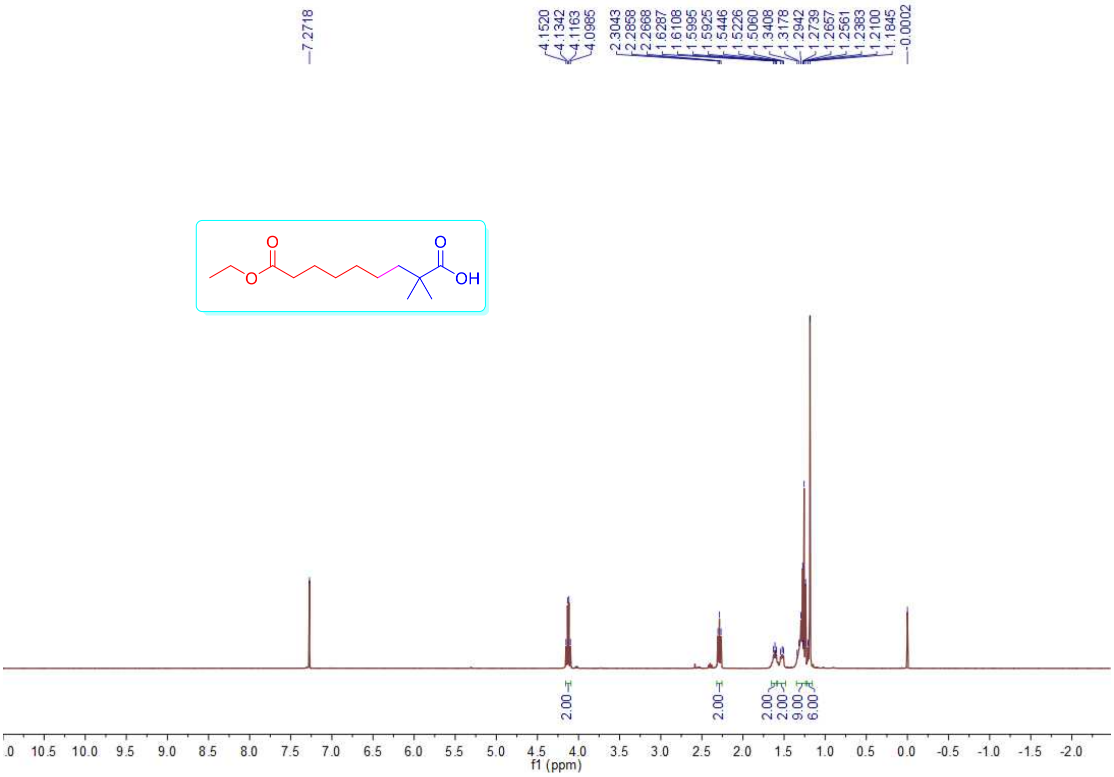

Supplementary Figure 69. ^{13}C NMR Spectrum of 7ef (101 MHz, $CDCl_3$)

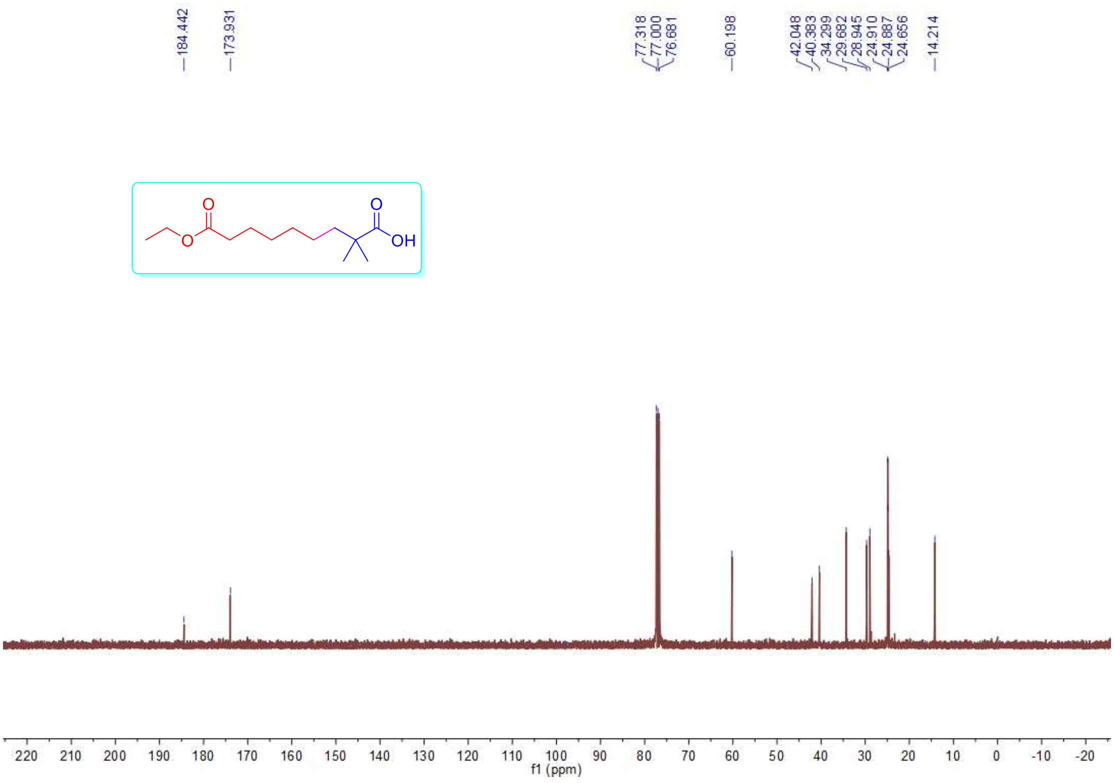

Supplementary Figure 70. ^{1}H NMR Spectrum of 8fl (400 MHz, $CDCl_3$)

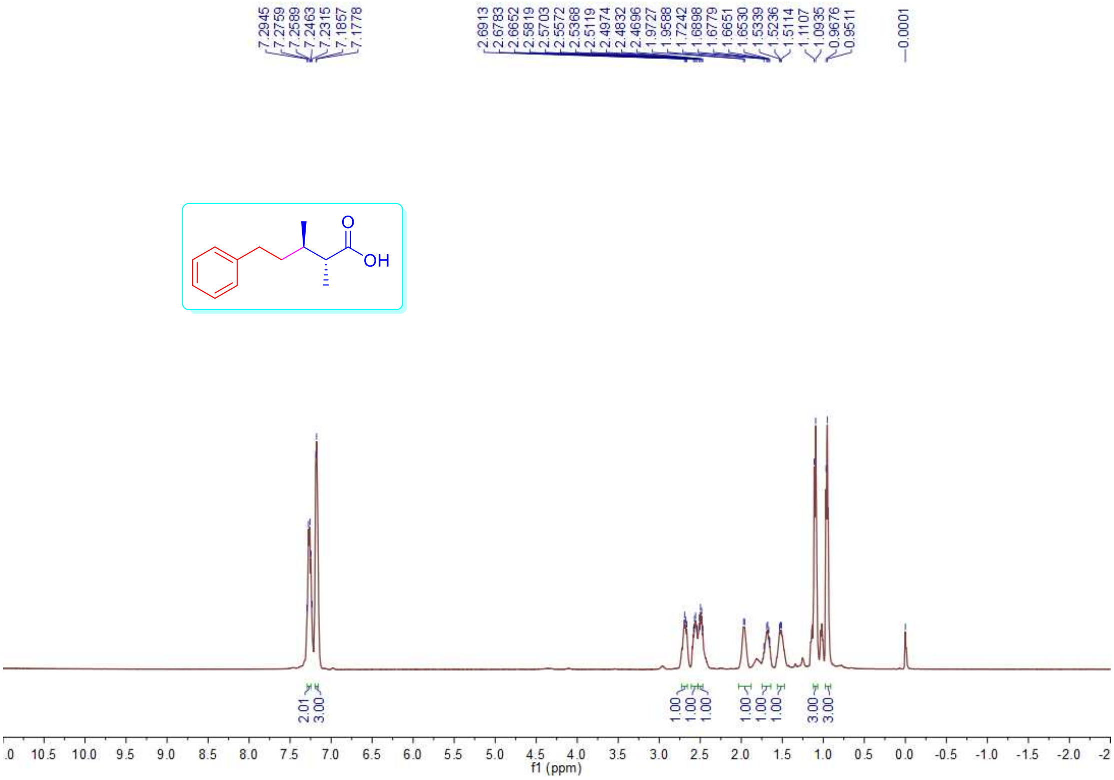

Supplementary Figure 71. ^{13}C NMR Spectrum of 8fl (101 MHz, $CDCl_3$)

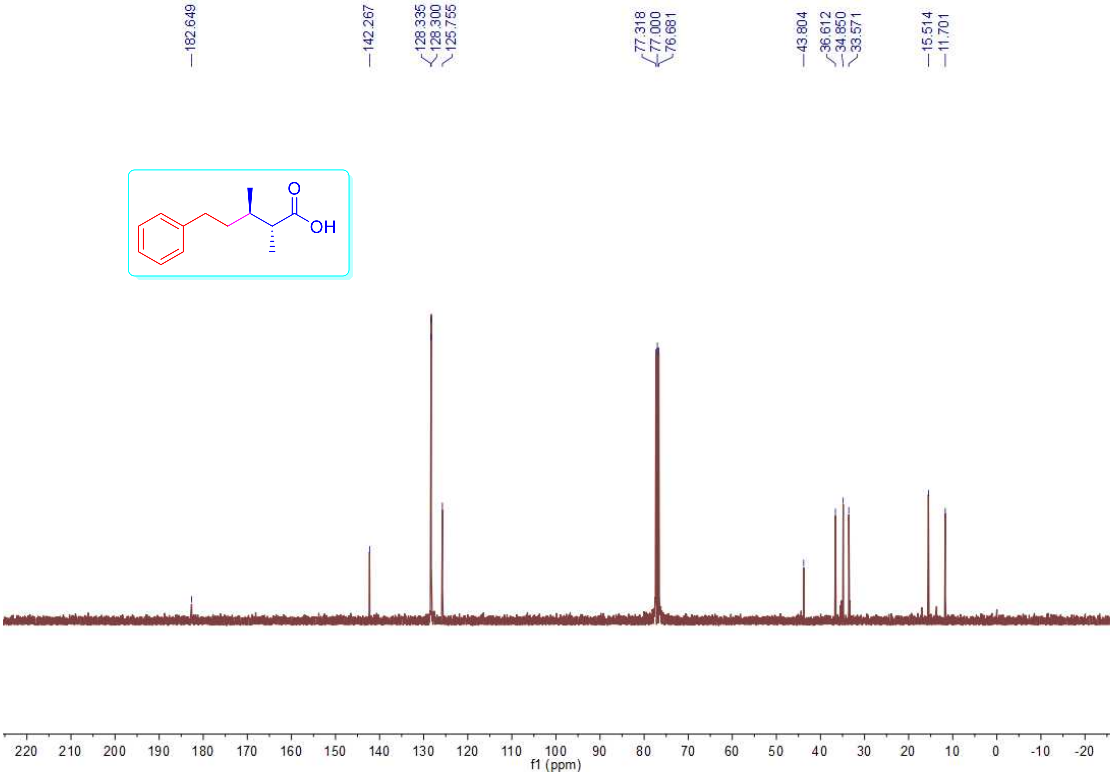

Supplementary Figure 72. ^{1}H NMR Spectrum of 8ff (400 MHz, $CDCl_3$)

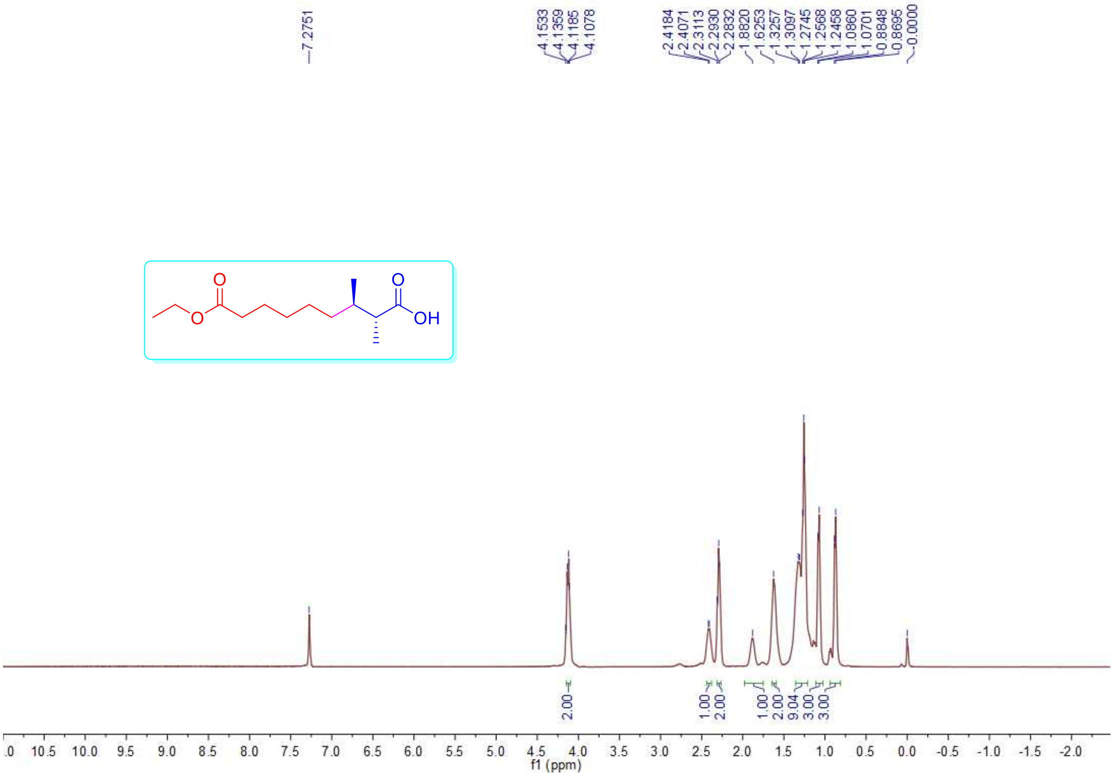

Supplementary Figure 73. ^{13}C NMR Spectrum of 8ff (101 MHz, $CDCl_3$)

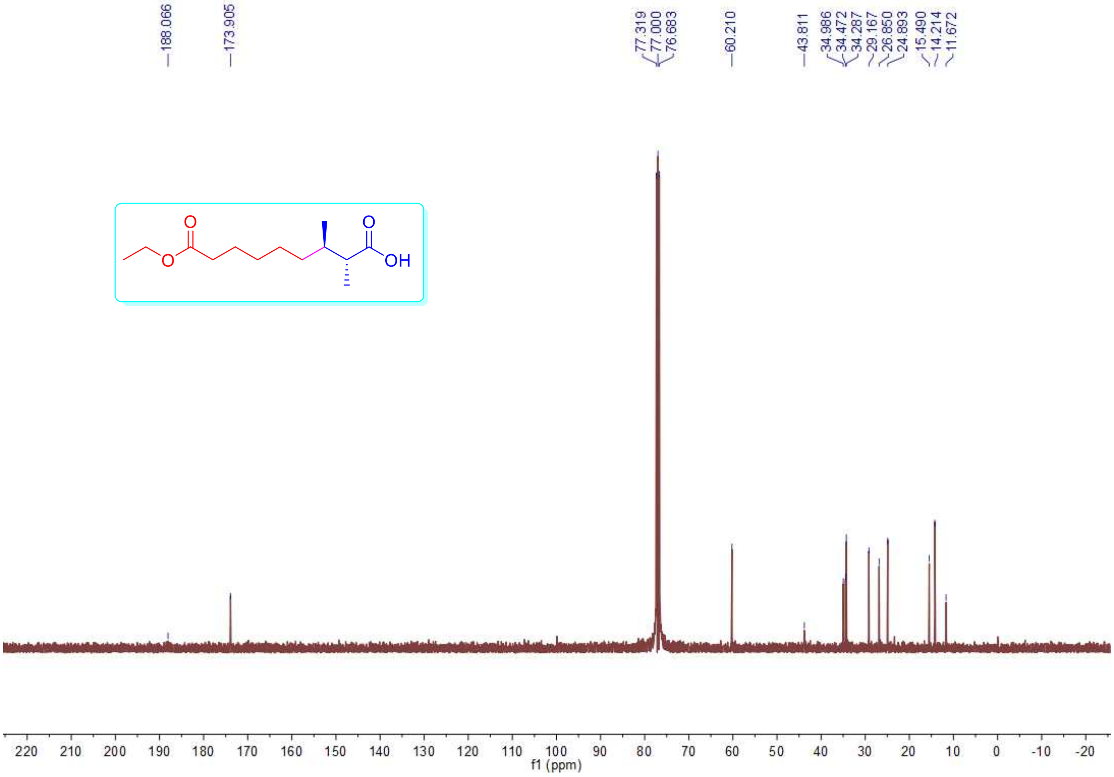

Supplementary Figure 74. ^{1}H NMR Spectrum of 9ge (400 MHz, $CDCl_3$)

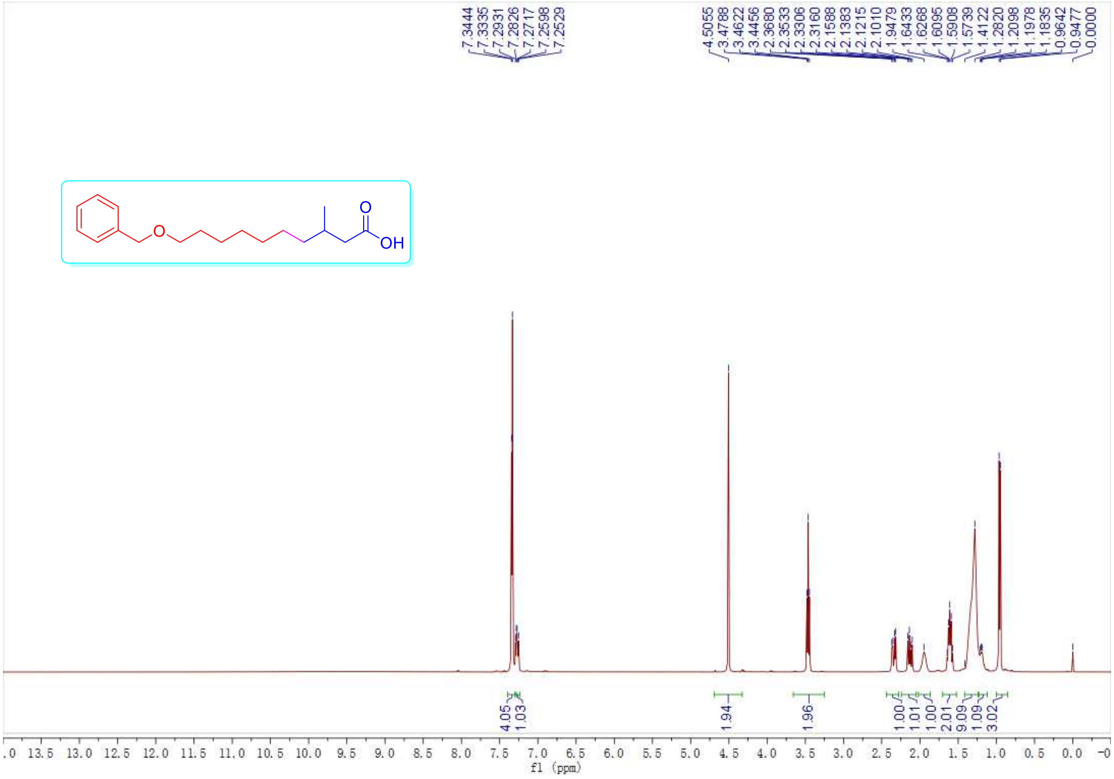

Supplementary Figure 75. ^{13}C NMR Spectrum of 9ge (101 MHz, $CDCl_3$)

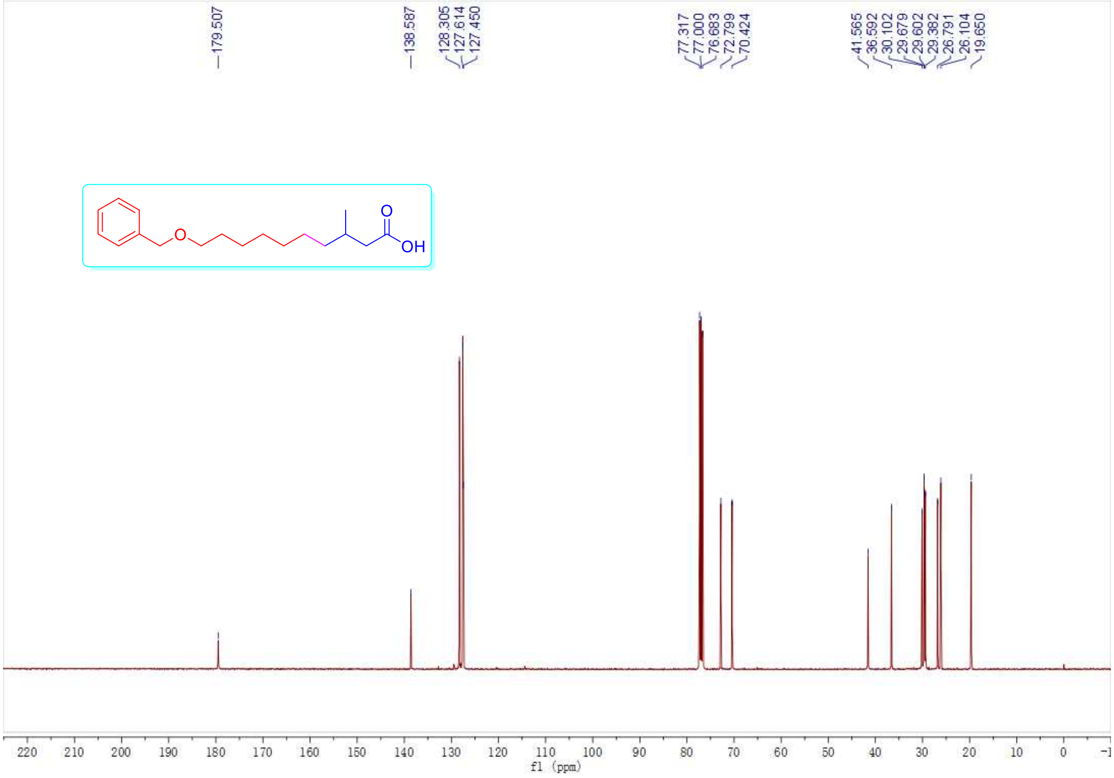

Supplementary Figure 76. ^{1}H NMR Spectrum of 9gf (400 MHz, $CDCl_3$)

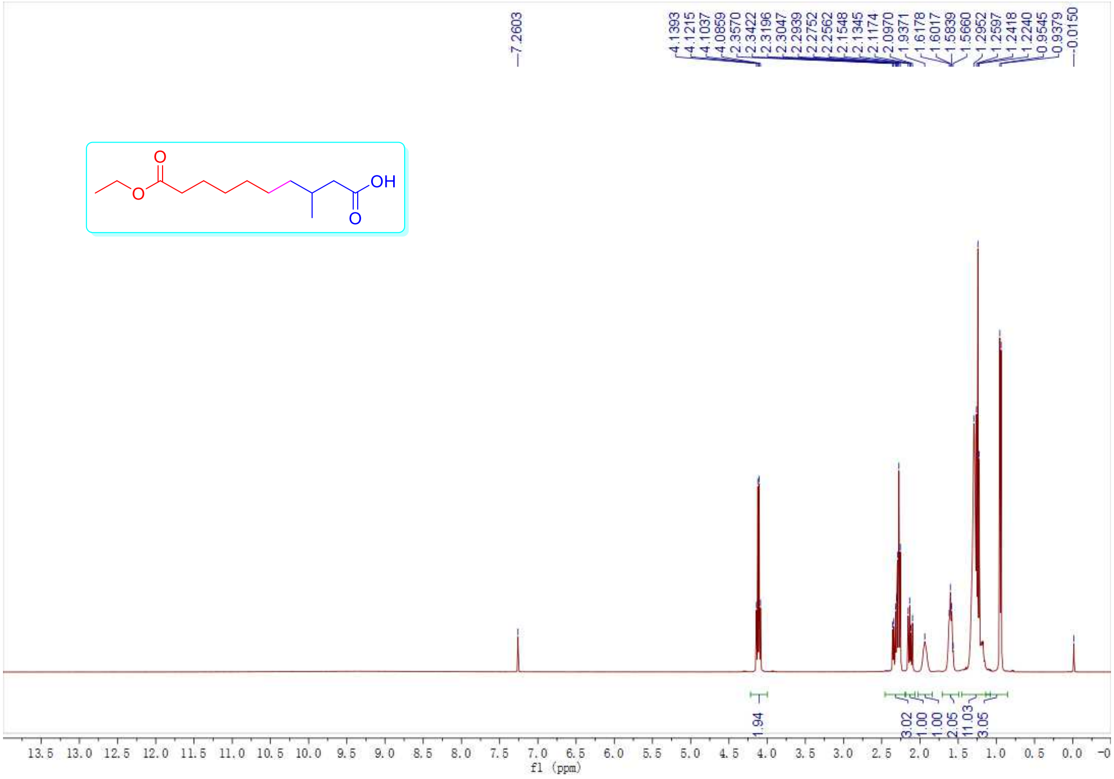

Supplementary Figure 77. ^{13}C NMR Spectrum of 9gf (101 MHz, $CDCl_3$)

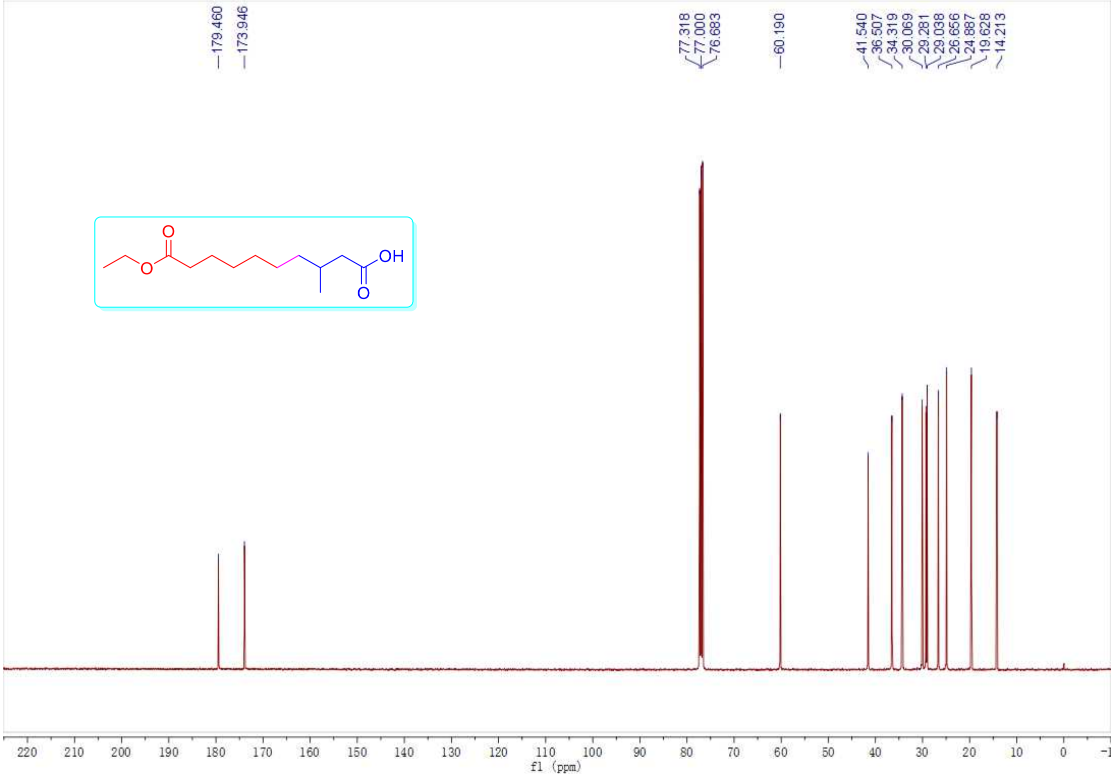

Supplementary Figure 78. ^{1}H NMR Spectrum of 10hc (400 MHz, $CDCl_3$)

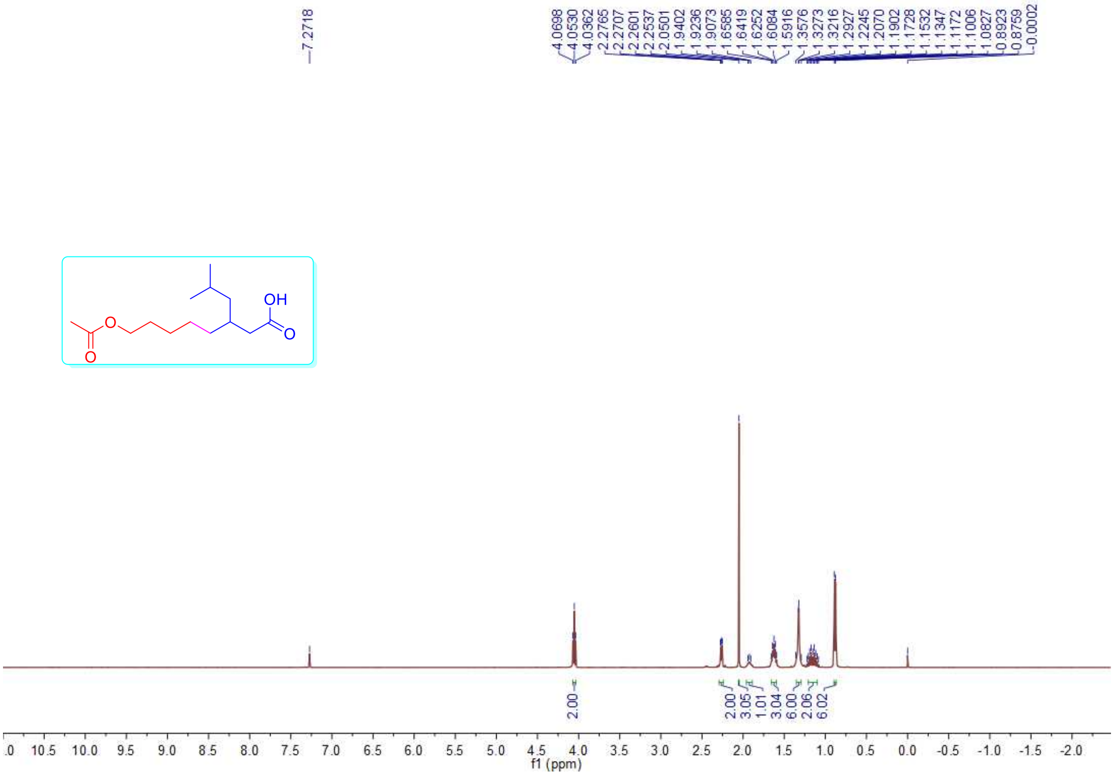

Supplementary Figure 79. ^{13}C NMR Spectrum of 10hc (101 MHz, $CDCl_3$)

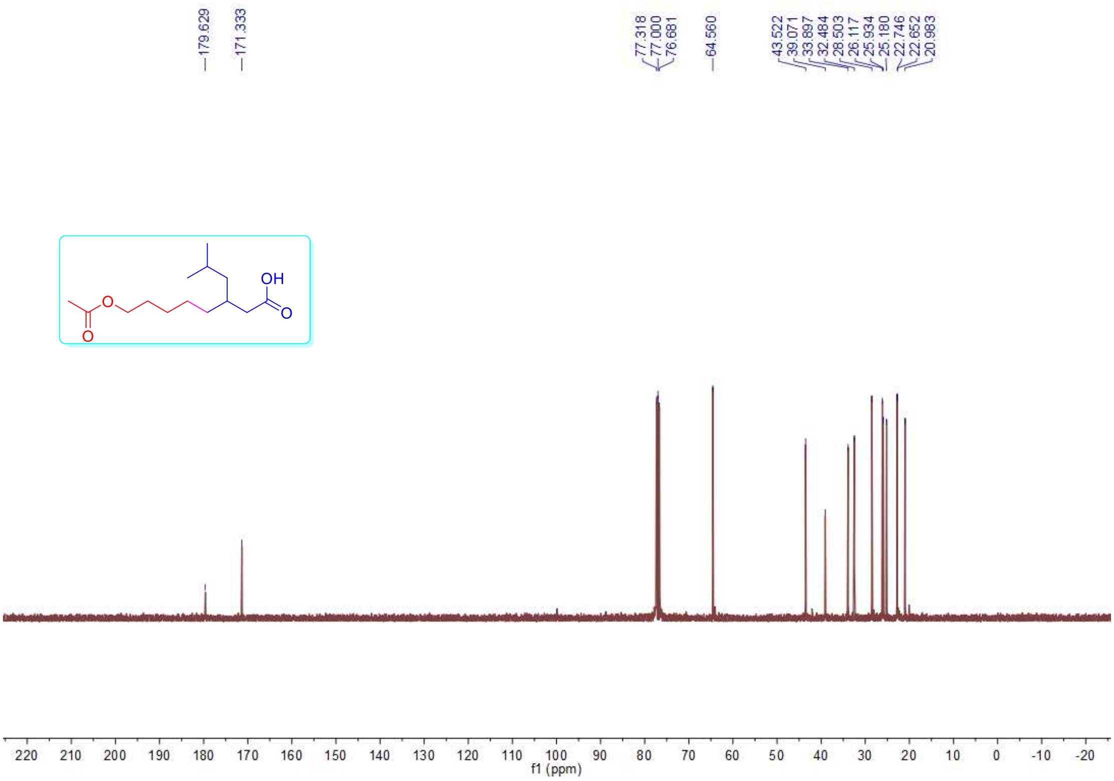

Supplementary Figure 80. ^{1}H NMR Spectrum of 10he (400 MHz, $CDCl_3$)

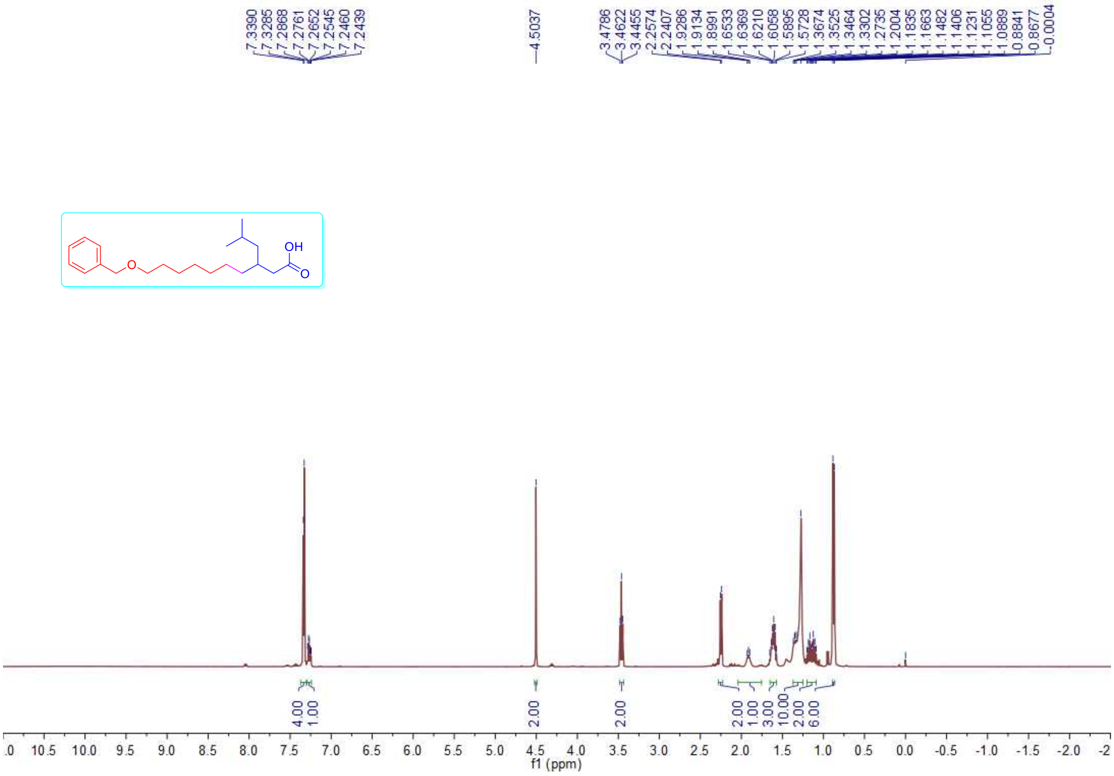

Supplementary Figure 81. ^{13}C NMR Spectrum of 10he (101 MHz, $CDCl_3$)

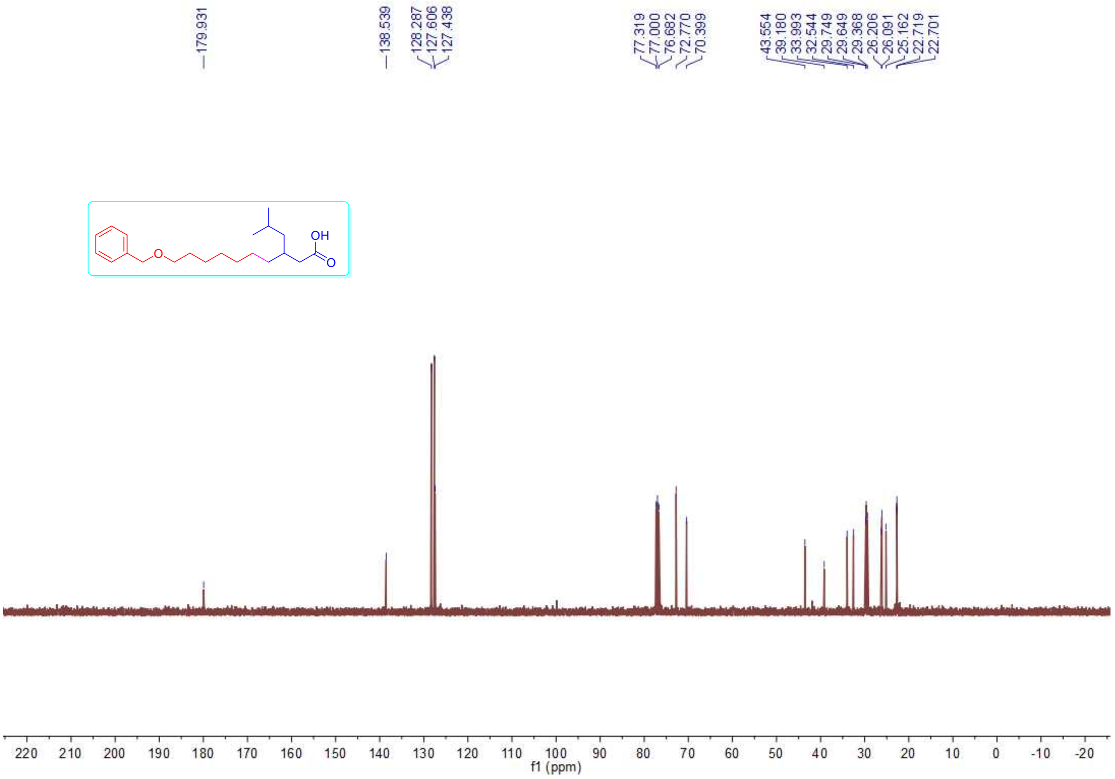

Supplementary Figure 82. ^{1}H NMR Spectrum of H (400 MHz, Methylene Chloride-d_2)

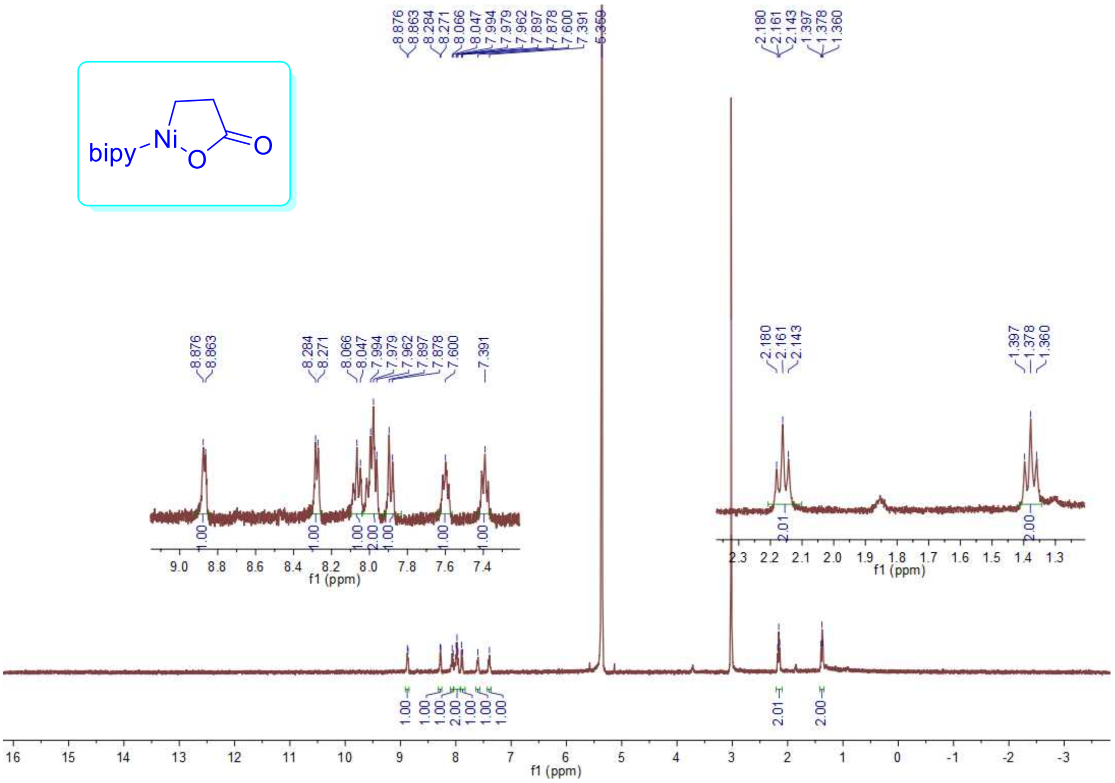

Supplementary Figure 83. ^{1}**H NMR Spectrum of G (400 MHz, Methylene Chloride-d$_2$)**

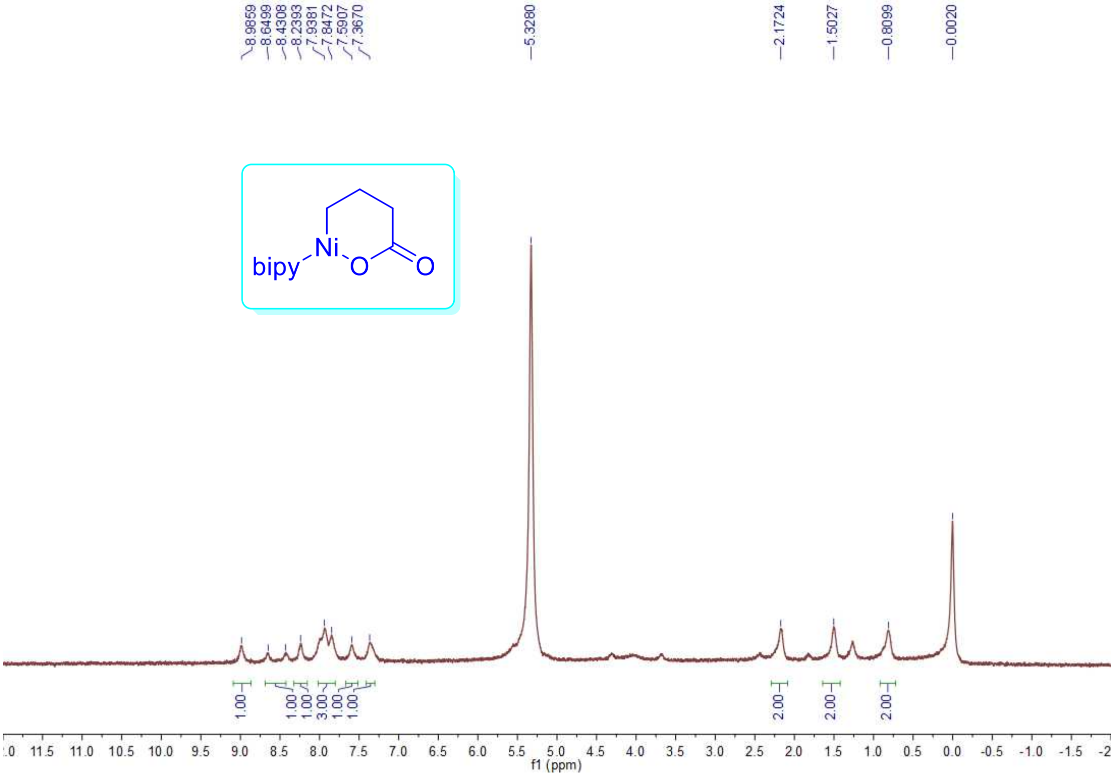

Supplementary Figure 84. ^{1}**H NMR Spectrum of 4bu' (400 MHz, $CDCl_3$)**

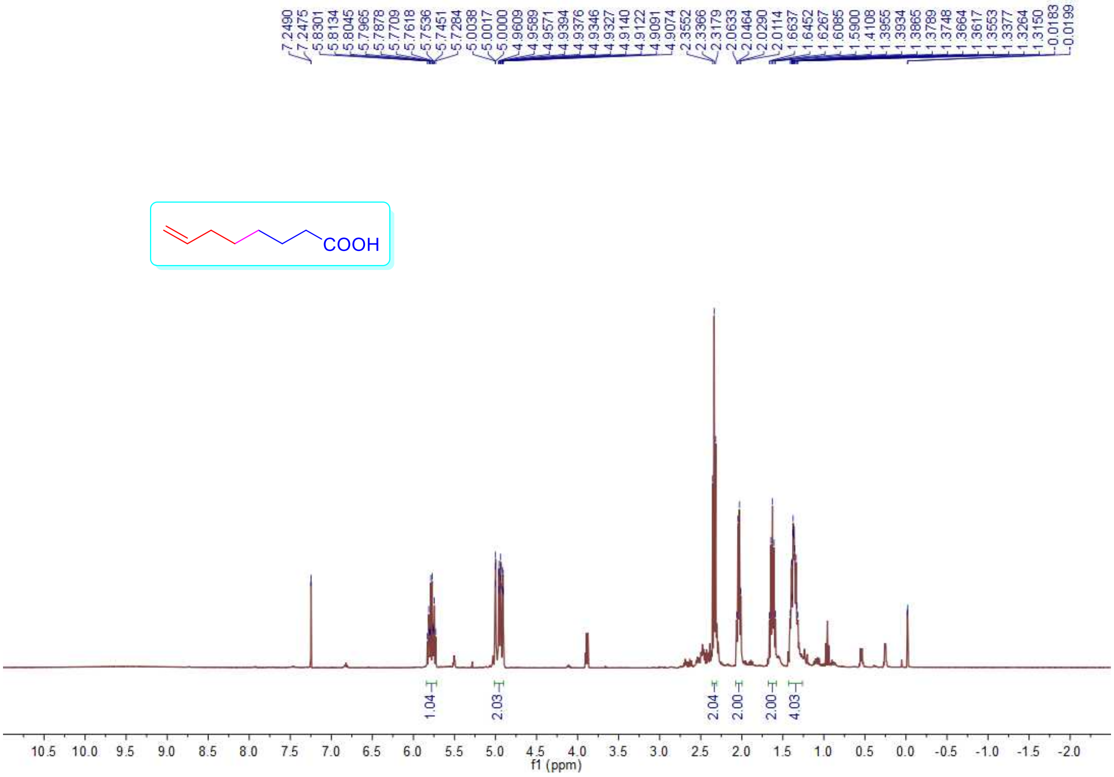

Supplementary Figure 85. ^{13}**C NMR Spectrum of 4bu' (101 MHz, $CDCl_3$)**

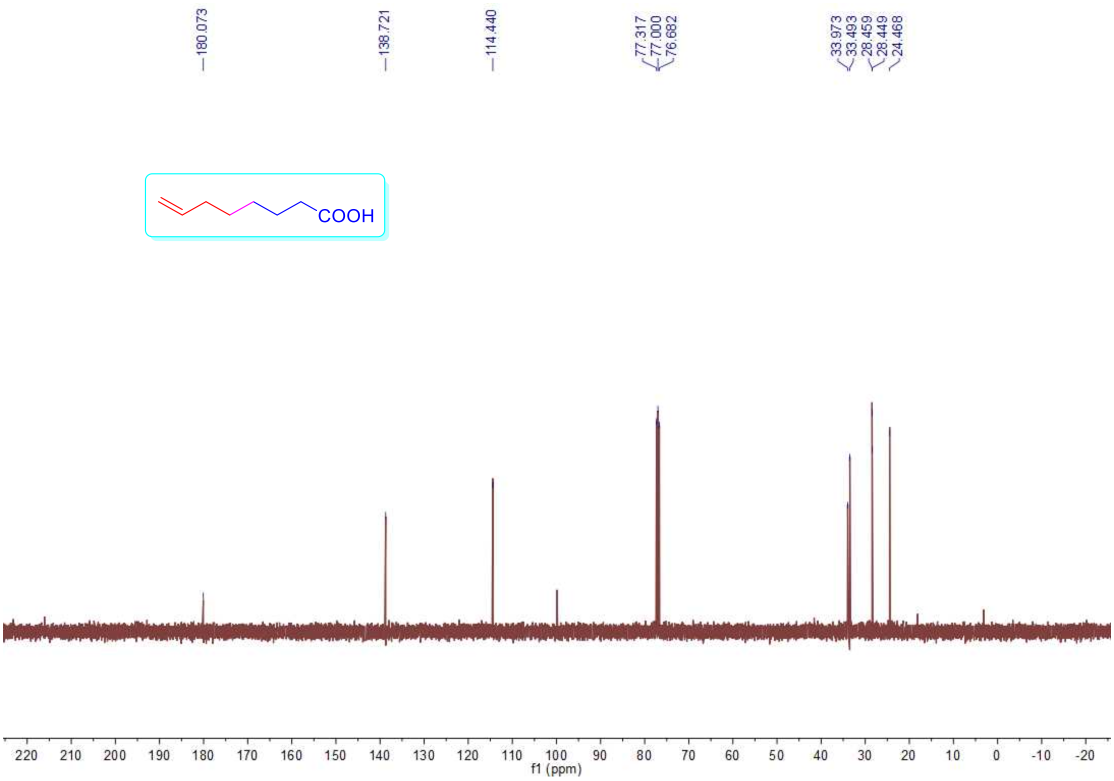

Supplementary Figure 86. ^{1}**H NMR Spectrum of 4bv (400 MHz, $CDCl_3$)**

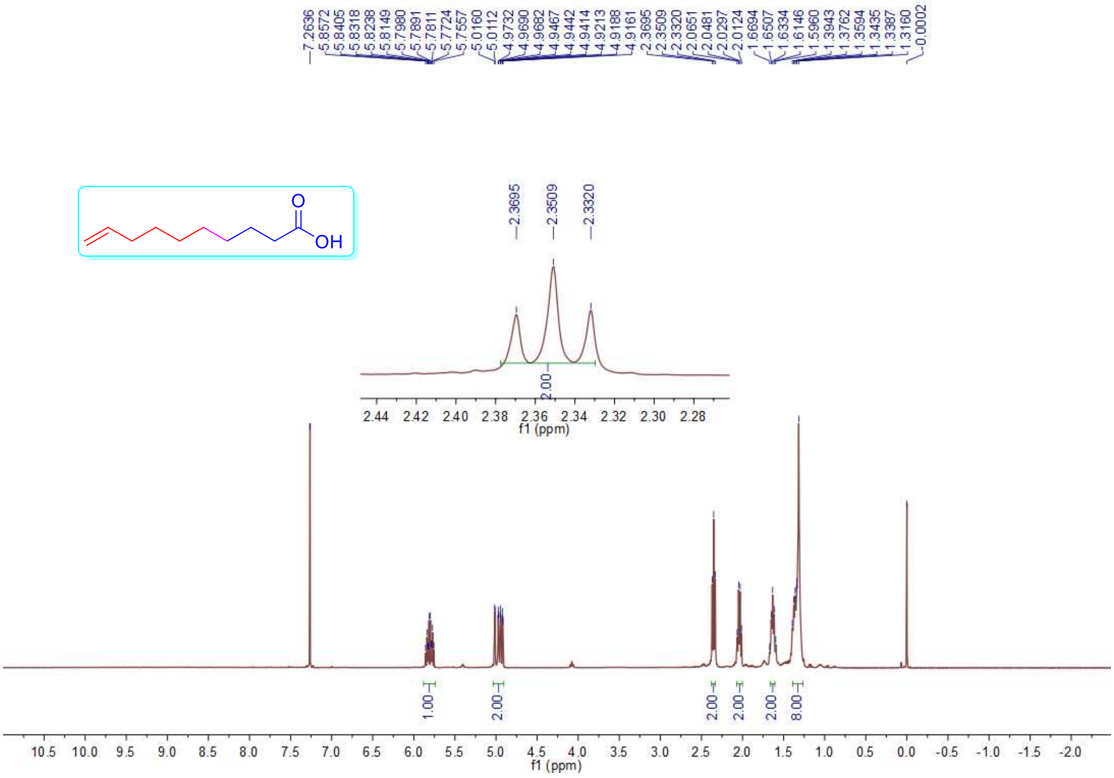

Supplementary Figure 87. ^{13}C NMR Spectrum of 4bv (101 MHz, $CDCl_3$)

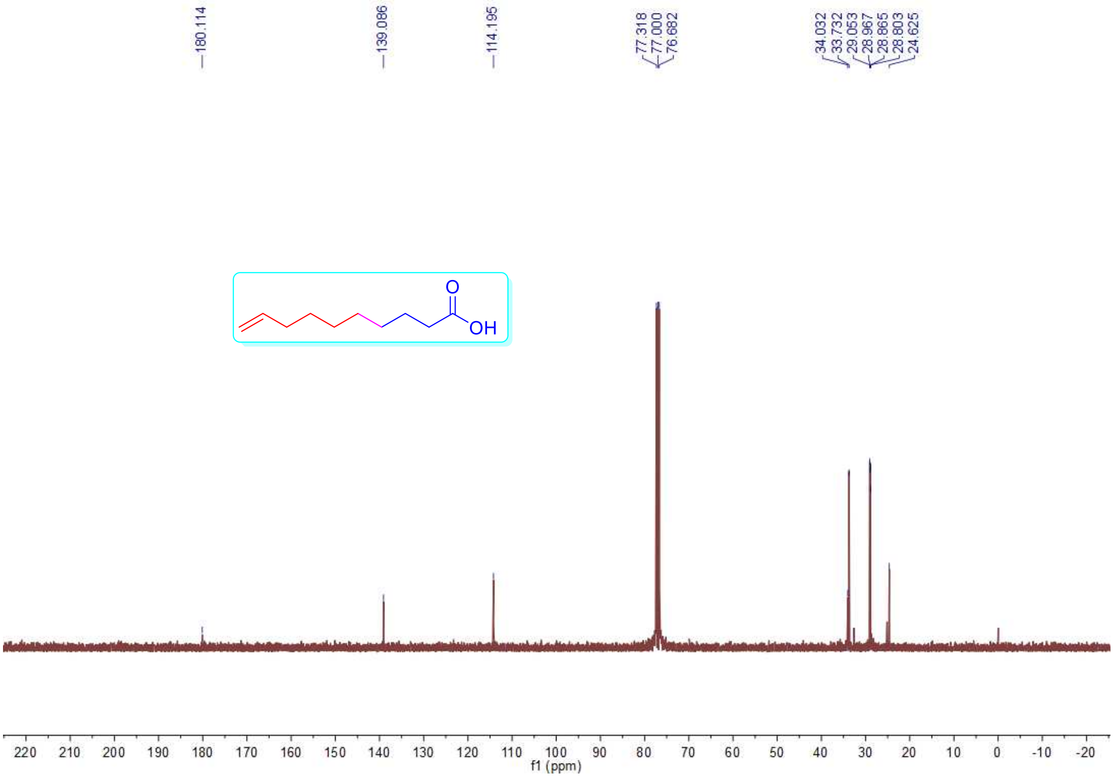

Supplementary Figure 88. ^{1}H NMR Spectrum of 4bv + 4bv'(400 MHz, $CDCl_3$)

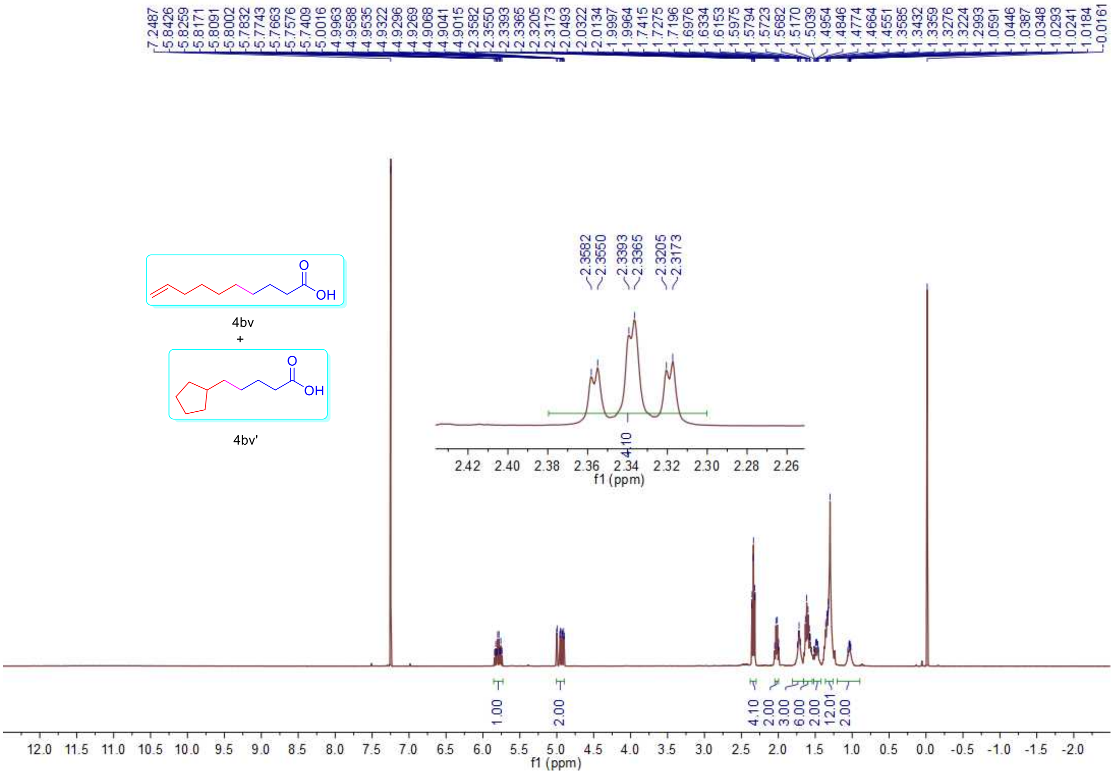

Supplementary Figure 89. ^{13}C NMR Spectrum of 4bv + 4bv' (101 MHz, $CDCl_3$)

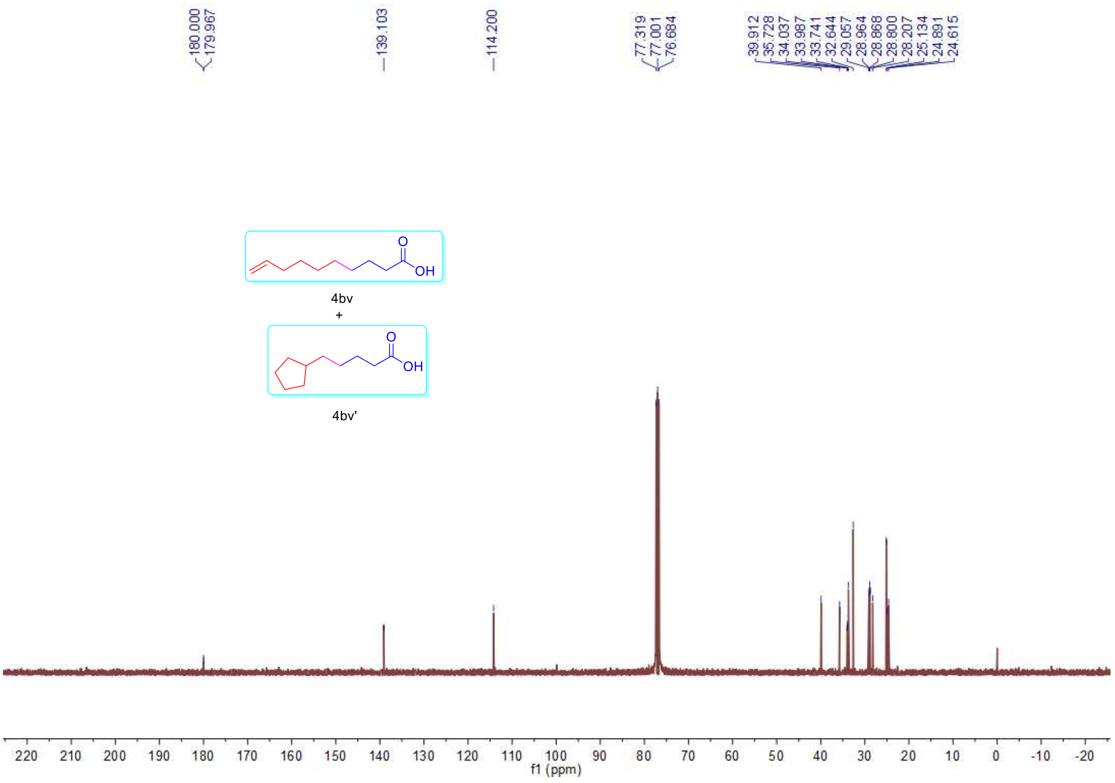

Supplementary Methods

All reactions were carried out under an atmosphere of dry argon. Anhydrous DMA was purchased from J&K and used without further purification. Zn powder was purchased from Sigma-Aldrich (median < 10 micron) and used without treated. Unless otherwise stated, reagents were commercially available and used as purchased. Chemicals were obtained from Sigma-Aldrich, Acros, Innochem, Energy Chemical, TCI China or Alfa Aesar. The progress of reactions was monitored by thin-layer chromatography using TLC plates and visualized by bromocresol green solution. Flash chromatography was performed with Qingdao Haiyang flash silica gel (200–300 mesh). The NMR spectra were obtained using a Brüker 400 MHz Fourier-transform NMR spectrometer. Chemical shifts were reported in units of parts per million (ppm) downfield from tetramethylsilane (TMS), and all coupling constants were reported in hertz. The infrared spectra were obtained with KBr plates using a IS10 FT-IR Spectrometer (ThermoFisher Corporation). High resolution mass spectrometry (HRMS) data were obtained on a Waters LC-TOF mass spectrometer (Xevo G2-XS QTof) using electrospray ionization (ESI) in positive or negative mode. Melting points were measured using a WRS-1C Melt-Temp apparatus and were uncorrected.

General procedure for the synthesis of aliphatic acids

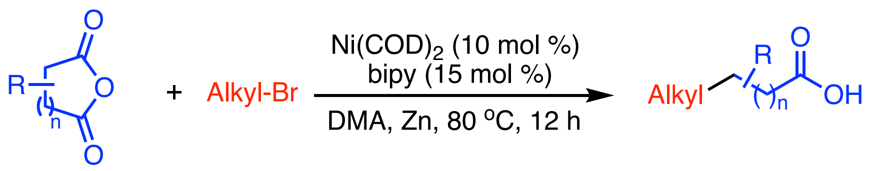

General Procedure **:** To an oven-dried microwave vial (10 mL) equipped with a stir bar (10 × 5 mm) was added $Ni(COD)_2$ (8.3 mg, 0.03 mmol) and bipy (7.0 mg, 0.045 mmol) under an argon atmosphere inside a glove box at 25 °C. Next, 0.225 mL of dry DMA was added via syringe to give a purple solution. After the catalyst/ligand solution was stirred for 1 h at 25 °C inside the glove box, Zn powder (39.2 mg, 0.6 mmol, 2.0 equiv) was added to the reaction vial followed by the monocyclic anhydride (0.45 mmol, 1.5 equiv) and alkyl bromide (0.3 mmol, 1.0 equiv). The microwave vial was sealed with a cap containing a rubber septum and removed from the glove box. The reaction mixture was stirred (~1000 rpm) at 80 °C for 12 h. The resulting gray solution was cooled to RT, quenched by addition of 5 drops water via syringe through the septum and then the vial opened to air. The reaction mixture was passed through a short flash column chromatography in silica gel (200–300 mesh) and rinsed with 5 mL of ethyl acetate to afford a yellow solution. The solvent and volatile materials were removed by rotary evaporator. The crude residue was purified by flash column chromatography in silica gel to yield the corresponding product.

General procedure for the synthesis of H and G

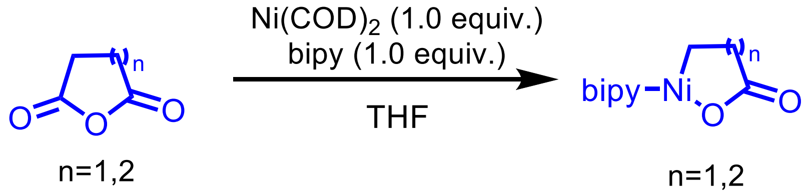

To an oven-dried microwave vial (10 mL) equipped with a stir bar (10 × 5 mm) was added $Ni(COD)_2$

(1.0 equiv.), bipy (1.0 equiv.) and anhydride (1.0 equiv.) under an argon atmosphere inside a glove box at 25 °C. Next, dry THF (1.0 equiv.) was added via syringe to give a purple solution. The reaction mixture was stirred for 6 h to obtain a red precipitate. The complex was filtered, washed with hexanes repeatedly and yield of a red product.

Supplementary Table 1. Optimization of the solvents

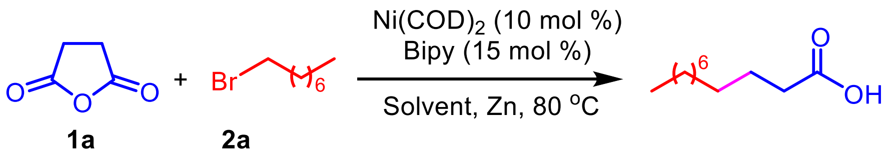

Entry	1a:2a	Zn/equiv	Conc./M	T/°C	Solvent	Yield[a] (%)
1	1.5:1	2	0.4	80	THF	34
2	1.5:1	2	0.4	80	2-Me-THF	trace
3	1.5:1	2	0.4	80	CPME	0
4	1.5:1	2	0.4	80	TBME	0
5	1.5:1	2	0.4	80	DMA	63
6	1.5:1	2	0.4	80	DMF	42
7	1.5:1	2	0.4	80	DMSO	12
8	1.5:1	2	0.4	80	CH_3CN	23
9	1.5:1	2	0.4	80	toluene	0
10	1.5:1	2	0.4	80	hexane	0
11	1.5:1	2	0.4	80	1,4-dioxane	0

[a]GC yields.

Supplementary Table 2. Optimization of the nickel sources

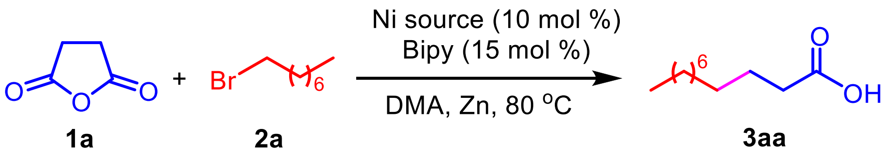

Entry	1a:2a	Zn/equiv	Conc./M	T/°C	Ni salts	Yield[a] (%)
1	1.5:1	2	0.4	80	NiF_2	trace
2	1.5:1	2	0.4	80	$NiCl_2$	50
3	1.5:1	2	0.4	80	$NiBr_2$	23
4	1.5:1	2	0.4	80	NiI_2	52
5	1.5:1	2	0.4	80	$NiCl_2(PPh_3)_2$	28
6	1.5:1	2	0.4	80	$NiBr_2 \cdot 3H_2O$	27
7	1.5:1	2	0.4	80	$NiBr_2 \cdot DME$	46
8	1.5:1	2	0.4	80	$NiCl_2 \cdot DME$	41
9	1.5:1	2	0.4	80	$Ni(COD)_2$	62

[a]GC yields.

Supplementary Table 3. Identification of the ligand

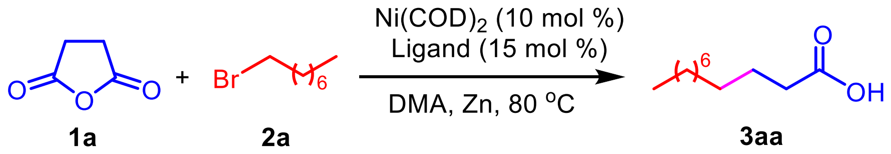

Entry	Ligand	1a:2a	Yield[a] (%)
1	L_1	1.5:1	63
2	L_2	1.5:1	0
3	L_3	1.5:1	0
4	L_4	1.5:1	32
5	L_5	1.5:1	26
6	L_6	1.5:1	25
7	L_7	1.5:1	0
8	L_8	1.5:1	21
9	L_9	1.5:1	0
10	L_{10}	1.5:1	32
11	L_{11}	1.5:1	0
12	L_{12}	1.5:1	0
13	L_{13}	1.5:1	0
14	L_{14}	1.5:1	36

[a]GC yields.

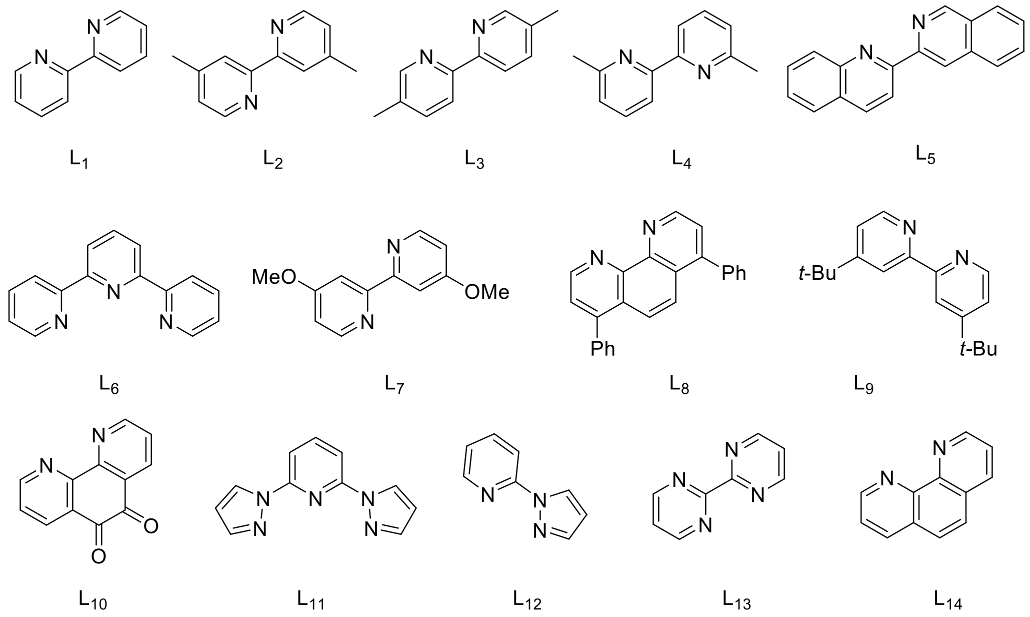

Supplementary Table 4. Optimization of the concentrations

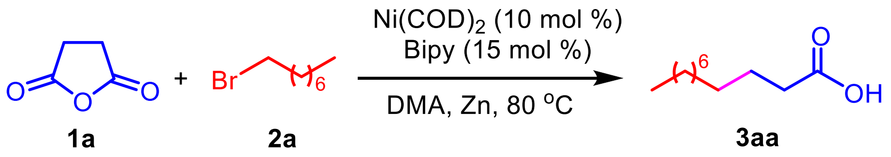

Entry	1a:2a	Zn/equiv	Conc./M	T/°C	t/h	Yield[a] (%)
1	1.5:1	2	0.4	80	12	62
2	1.5:1	2	0.5	80	12	61
3	1.5:1	2	0.7	80	12	62
4	1.5:1	2	0.8	80	12	63
5	1.5:1	2	1.0	80	12	70
6	1.5:1	2	1.3	80	12	87
7	1.5:1	2	2.0	80	12	86

[a]GC yields.

Supplementary Table 5. Control experiments

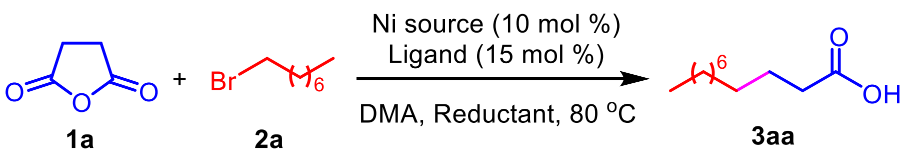

Entry	Ni source	Ligand	Reductant	Yield[a] (%)
1	$Ni(COD)_2$	bipy	Zn	86
2	none	bipy	Zn	0
3	$Ni(COD)_2$	none	Zn	trace
4	$Ni(COD)_2$	bipy	none	<5
5	$Ni(COD)_2$	bipy	Mn	68
6	$Ni(COD)_2$	bipy	Mg	25

[a]Isolated yields.

Optimization of the alkyl halides

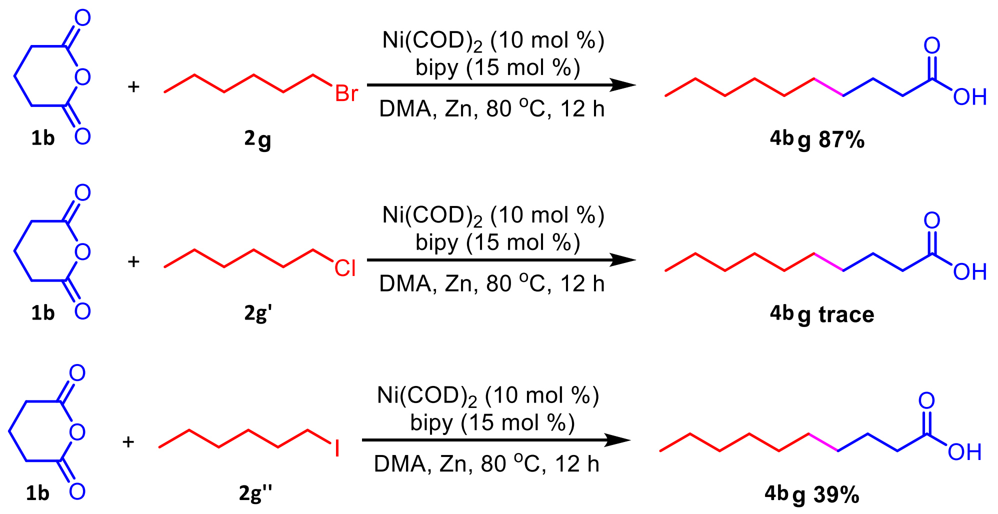

Scale-up Transformation to 10.0 mmol for the Synthesis of Aliphatic Acid 4bj

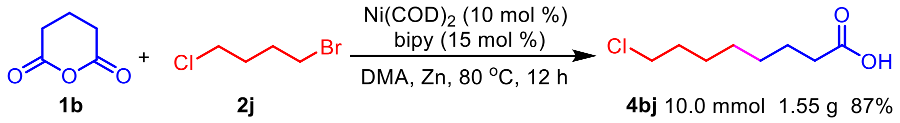

To an oven-dried microwave vial (10 mL) equipped with a stir bar (10 × 5 mm) was added Ni(COD)$_2$ (275 mg, 1.0 mmol) and bipy (234 mg, 1.5 mmol) under an argon atmosphere inside a glove box at 25 °C. Next, 7.5 mL of dry DMA was added via syringe to give a purple solution. After the catalyst/ligand solution was stirred for 1 h at 25 °C inside the glove box, Zn powder (1.3 g, 20 mmol, 2.0 equiv) was added to the reaction vial followed by the glutaric anhydride (1.71 g, 15.0 mmol, 1.5 equiv) and 1-bromo-4-chlorobutane (**2j**) (1.15 mL, 10.0 mmol, 1.0 equiv). The microwave vial was sealed with a cap possessing a rubber septum and removed from the glove box. The reaction mixture was stirred (~1000 rpm) at 80 °C for 12 h. The resulting gray solution was then cooled to RT, quenched by addition of 10 drops water via syringe and then the vial opened to air. The reaction mixture was passed through a short pad of silica gel and rinsed with 10 mL of ethyl acetate to afford a yellow solution. The solvent and volatile materials were removed by rotary evaporator. The crude residue was purified by flash column chromatography on silica gel (eluted with Petroleum ether:EtOAc = 5:1) to give **4bj** (1.55 g, 87%) as a colorless oil.

Determine β-hydride elimination does not occur after CO elimination

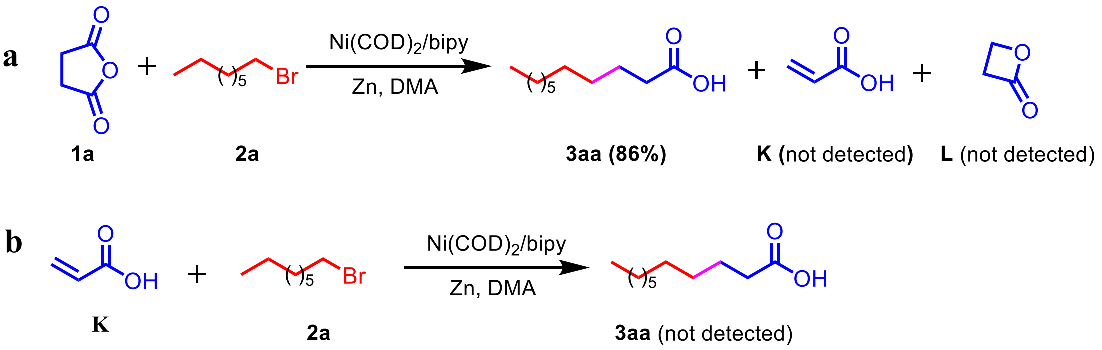

In order to determine β-hydride elimination does not occur after CO elimination in the reaction of cyclic anhydrides to form Ni homoenolate intermediates, succinic anhydride (**1a**) and 1-bromooctane (**2a**) were employed under the standard conditions. When GC and GCMS were used to monitor the reaction mixture, an 86% yield of **3aa** was obtained, we did not find the acrylic acid **K** or acrylic lactone **L**. In addition, we used acrylic acid **K** instead of succinic anhydride **1a** as starting material under the standard conditions. We did not detect product **3aa** in this reaction mixture. We hypothesize that β-hydride elimination is diminished in the presence of carbon monoxide, which can occupy a coordination site on the nickel and block β-H elimination. Taken together, these results suggest that β-hydride elimination after CO deinsertion did not occur.

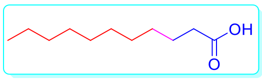

Undecanoic acid (3aa). The reaction was performed following the General Procedure with $Ni(COD)_2$ (8.3 mg, 0.03 mmol), bipy (7.0 mg, 0.045 mmol), **1a** (45.0 mg, 0.45 mmol), Zn powder (39.2 mg, 0.6 mmol), and 1-bromooctane (**2a**) (51.8 µL, 0.3 mmol). The crude product was purified by flash chromatography on silica gel (eluted with Petroleum ether:EtOAc = 5:1) to give the product (47.0 mg, 84% yield) as a colorless oil. R_f = 0.81 (Petroleum ether:EtOAc = 2:1). The spectroscopic data for this product match the literature data[1].

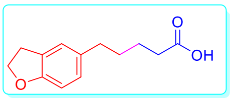

5-(2,3-dihydrobenzofuran-5-yl)pentanoic acid (3ab). The reaction was performed following the General Procedure with $Ni(COD)_2$ (8.3 mg, 0.03 mmol), bipy (7.0 mg, 0.045 mmol), **1a** (45.0 mg, 0.45 mmol), Zn powder (39.2 mg, 0.6 mmol), and 6-(2-bromoethyl)-2,3-dihydrobenzofuran (**2b**) (68.1 mg, 0.3 mmol). The crude product was purified by flash chromatography on silica gel (eluted with Petroleum ether:EtOAc = 5:1) to give the product (54.2 mg, 82% yield) as a white solid. Mp: 80.6 – 82.3 ºC. R_f = 0.66 (Petroleum ether:EtOAc = 2:1). ^{1}H NMR (400 MHz, $CDCl_3$) δ: 7.00 (s, 1H), 6.89 (d, J = 8.1 Hz, 1H), 6.69 (d, J = 8.1 Hz, 1H), 4.53 (t, J = 8.7 Hz, 2H), 3.17 (t, J = 8.7 Hz, 2H), 2.55 (t, J = 7.1 Hz, 2H), 2.36 (t, J = 7.0 Hz, 2H), 1.69 – 1.61 (m, 4H) ppm. ^{13}C{^{1}H} NMR (101 MHz, $CDCl_3$) δ: 179.9 , 158.2 , 133.9 , 127.7 , 126.9 , 124.8 , 108.9 , 71.1 , 34.9 , 33.9 , 31.2 , 29.8 , 24.2 ppm. IR(neat): 3438, 3052, 2948, 2934, 2856, 1701, 1653, 1492, 1409, 1320, 1247, 1202, 981, 816, 737 cm^{-1}. HRMS: calcd for $C_{13}H_{17}O_3$ $[M+H]^+$ 221.1178, found 221.1177.

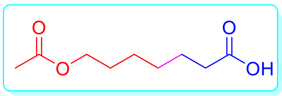

7-acetoxyheptanoic acid (3ac). The reaction was performed following the General Procedure with $Ni(COD)_2$ (8.3 mg, 0.03 mmol), bipy (7.0 mg, 0.045 mmol), **1a** (45.0 mg, 0.45 mmol), Zn powder (39.2 mg, 0.6 mmol),

and 4-bromobutyl acetate (**2c**) (43.4 μL, 0.3 mmol). The crude product was purified by flash chromatography on silica gel (eluted with Petroleum ether:EtOAc = 5:1) to give the product (39.5 mg, 70% yield) as a colorless oil. R_f = 0.63 (Petroleum ether:EtOAc = 2:1). ^{1}H NMR (400 MHz, $CDCl_3$) δ: 4.06 (t, J = 6.7 Hz, 2H), 2.36 (t, J = 7.4 Hz, 2H), 2.05 (s, 3H), 1.67 – 1.60 (m, 4H), 1.40 – 1.36 (m, 4H) ppm. ^{13}C{^{1}H} NMR (101 MHz, $CDCl_3$) δ: 179.6 , 171.3 , 64.4 , 33.9 , 28.6 , 28.3 , 25.6 , 24.5 , 20.9 ppm. IR(neat): 3459, 2940, 2863, 1737, 1721, 1392, 1248, 1037, 889, 764, 608, 484, 450 cm^{-1}. HRMS: calcd for $C_9H_{17}O_4$ [M+H]$^+$ 189.1127, found 189.1129.

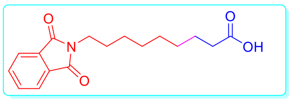

9-(1,3-dioxoisoindolin-2-yl)nonanoic acid (3ad). The reaction was performed following the General Procedure with Ni(COD)$_2$ (8.3 mg, 0.03 mmol), bipy (7.0 mg, 0.045 mmol), **1a** (45.0 mg, 0.45 mmol), Zn powder (39.2 mg, 0.6 mmol), and *N*-(6-bromohexyl)phthalimide (**2d**) (93.1 mg, 0.3 mmol). The crude product was purified by flash chromatography on silica gel (eluted with Petroleum ether:EtOAc = 5:1) to give the product (82.8 mg, 91% yield) as a white solid. Mp: 77.8 – 78.8 ºC. R_f = 0.48 (Petroleum ether:EtOAc = 2:1). ^{1}H NMR (400 MHz, $CDCl_3$) δ: 7.85 – 7.83 (m, 2H), 7.72 – 7.70 (m, 2H), 3.67 (t, J = 7.3 Hz, 2H), 2.34 (t, J = 7.5 Hz, 2H), 1.69 – 1.60 (m, 4H), 1.36 – 1.33 (m, 8H) ppm. ^{13}C{^{1}H} NMR (101 MHz, $CDCl_3$) δ: 179.8 , 168.4 , 133.8 , 132.1 , 123.1 , 37.9 , 33.9 , 28.96 , 28.94 , 28.5 , 26.7 , 24.5 ppm. One resonance was not observed due to overlapping resonances. IR(neat): 3462, 3099, 2932, 2856, 1772, 1716, 1615, 1465, 1396, 1233, 1055, 930, 720, 711, 531 cm^{-1}. HRMS: calcd for $C_{17}H_{22}NO_4$ [M+H]$^+$ 304.1549, found 304.1545.

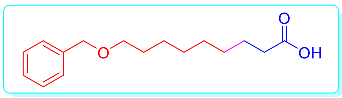

9-(benzyloxy)nonanoic acid (3ae). The reaction was performed following the General Procedure with Ni(COD)$_2$ (8.3 mg, 0.03 mmol), bipy (7.0 mg, 0.045 mmol), **1a** (45.0 mg, 0.45 mmol), Zn powder (39.2 mg, 0.6 mmol), and 6-benzyloxyhexyl bromide (**2e**) (81.4 mg, 0.3 mmol). The crude product was purified by flash chromatography on silica gel (eluted with Petroleum ether:EtOAc = 5:1) to give the product (53.9 mg, 68% yield) as a colorless oil. R_f = 0.57 (Petroleum ether:EtOAc = 2:1). ^{1}H NMR (400 MHz, $CDCl_3$) δ: 7.34 – 7.33 (m, 4H), 7.30 – 7.26 (m, 1H), 4.50 (s, 2H), 3.46 (t, J = 6.6 Hz, 2H), 2.34 (t, J = 7.5 Hz, 2H), 1.64 – 1.57 (m, 4H), 1.35 – 1.31 (m, 8H) ppm. ^{13}C{^{1}H} NMR (101 MHz, $CDCl_3$) δ: 179.9 , 138.6 , 128.3 , 127.6 , 127.5 , 72.8 , 70.4 , 34.0 , 29.7 , 29.2 , 29.1 , 29.0 , 26.1 , 24.6 ppm. IR(neat): 3668, 3114, 2935, 2857, 1704, 1683, 1558, 1453, 1362, 1205, 1098, 849, 738, 698, 523, 461, 419 cm^{-1}. HRMS: calcd for $C_{16}H_{25}O_3$ [M+H]$^+$ 265.1804, found 265.1804.

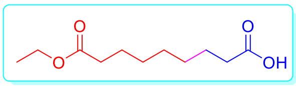

9-ethoxy-9-oxononanoic acid (3af). The reaction was performed following the General Procedure with Ni(COD)$_2$ (8.3 mg, 0.03 mmol), bipy (7.0 mg, 0.045 mmol), **1a** (45.0 mg, 0.45 mmol), Zn powder (39.2 mg, 0.6 mmol), and ethyl 6-bromohexanoate (**2f**) (53.4 μL, 0.3 mmol). The crude product was purified by flash chromatography on silica gel (eluted with Petroleum ether:EtOAc = 5:1) to give the product (46.7 mg, 72% yield) as a colorless oil. R_f = 0.61 (Petroleum ether:EtOAc = 2:1). ^{1}H NMR (400 MHz, $CDCl_3$) δ: 4.11 (q, J = 7.1 Hz, 2H), 2.33 (t, J = 7.5 Hz, 2H), 2.27 (t, J = 7.5 Hz, 2H), 1.63 – 1.58 (m, 4H), 1.31 – 1.30 (m, 6H), 1.24 (t, J = 7.1 Hz, 3H) ppm. ^{13}C{^{1}H} NMR (101 MHz, $CDCl_3$) δ: 179.9 , 173.9 , 60.2 , 34.3 , 34.0 , 28.9 , 28.82 , 28.80 , 24.8 , 24.5 , 14.2 ppm. IR(neat): 3627, 1658, 1651, 1373, 1257, 1185, 1096, 748, 625, 514, 482, 452, 421 cm^{-1}. HRMS: calcd for $C_{11}H_{21}O_4$ [M+H]$^+$ 217.1440, found 217.1440.

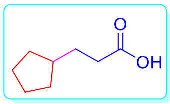

3-cyclopentylpropanoic acid (3an). The reaction was performed following the General Procedure with $Ni(COD)_2$ (8.3 mg, 0.03 mmol), bipy (7.0 mg, 0.045 mmol), **1a** (45.0 mg, 0.45 mmol), Zn powder (39.2 mg, 0.6 mmol), and bromocyclopentane (**2n**) (32.2 μL, 0.3 mmol). The crude product was purified by flash chromatography on silica gel (eluted with Petroleum ether:EtOAc = 5:1) to give the product (24.3 mg, 57% yield) as a colorless oil. R_f = 0.79 (Petroleum ether:EtOAc = 2:1). ^{1}H NMR (400 MHz, $CDCl_3$) δ: 2.38 (t, J = 7.0 Hz, 2H), 1.83 – 1.78 (m, 3H), 1.68 – 1.52 (m, 5H), 1.30 – 1.26 (m, 1H), 1.10 – 1.09 (m, 2H) ppm. $^{13}C\{^1H\}$ NMR (101 MHz, $CDCl_3$) δ: 180.3 , 39.6 , 32.4 , 30.9 , 25.1 ppm. One resonance was not observed due to overlapping resonances. IR(neat): 2431, 2950, 2868, 1709, 1650, 1453, 1413, 1283, 1214, 1090, 936, 816, 632, 505, 430 cm^{-1}. HRMS: calcd for $C_8H_{15}O_2$ $[M+H]^+$ 143.1072, found 143.1072.

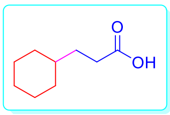

3-cyclohexylpropanoic acid (3ao). The reaction was performed following the General Procedure with $Ni(COD)_2$ (8.3 mg, 0.03 mmol), bipy (7.0 mg, 0.045 mmol), **1a** (45.0 mg, 0.45 mmol), Zn powder (39.2 mg, 0.6 mmol), and bromocyclohexane (**2o**) (36.9 μL, 0.3 mmol). The crude product was purified by flash chromatography on silica gel (eluted with Petroleum ether:EtOAc = 5:1) to give the product (31.9 mg, 68% yield) as a colorless oil. R_f = 0.81 (Petroleum ether:EtOAc = 2:1). The spectroscopic data for this product match the literature data[2].

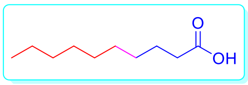

decanoic acid (4bg). The reaction was performed following the General Procedure with $Ni(COD)_2$ (8.3 mg, 0.03 mmol), bipy (7.0 mg, 0.045 mmol), **1b** (51.3 mg, 0.45 mmol), Zn powder (39.2 mg, 0.6 mmol), and 1-bromohexane (**2g**) (42.1 μL, 0.3 mmol). The crude product was purified by flash chromatography on silica gel (eluted with Petroleum ether:EtOAc = 5:1) to give the product (45.0 mg, 87% yield) as a colorless oil. R_f = 0.78 (Petroleum ether:EtOAc = 2:1). The spectroscopic data for this product match the literature data[3].

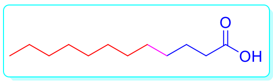

dodecanoic acid (4ba). The reaction was performed following the General Procedure with $Ni(COD)_2$ (8.3 mg, 0.03 mmol), bipy (7.0 mg, 0.045 mmol), **1b** (51.3 mg, 0.45 mmol), Zn powder (39.2 mg, 0.6 mmol), and 1-bromooctane (**2a**) (51.8 μL, 0.3 mmol). The crude product was purified by flash chromatography on silica gel (eluted with Petroleum ether:EtOAc = 5:1) to give the product (55.3 mg, 92% yield) as a colorless oil. R_f = 0.78 (Petroleum ether:EtOAc = 2:1). The spectroscopic data for this product match the literature data[4].

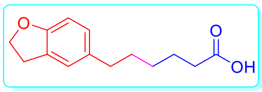

6-(2,3-dihydrobenzofuran-5-yl)hexanoic acid (4bb). The reaction was performed following the General Procedure with $Ni(COD)_2$ (8.3 mg, 0.03 mmol), bipy (7.0 mg, 0.045 mmol), **1b** (51.3 mg, 0.45 mmol), Zn powder (39.2 mg, 0.6 mmol), and 6-(2-bromoethyl)-2,3-dihydrobenzofuran (**2b**) (68.1 mg, 0.3 mmol). The crude product was purified by flash chromatography on silica gel (eluted with Petroleum ether:EtOAc = 5:1) to give the product (61.2 mg, 87% yield) as a white solid. Mp: 87.8 – 89.6 ºC. R_f = 0.63 (Petroleum ether:EtOAc = 2:1). ^{1}H NMR (400 MHz, $CDCl_3$) δ: 7.00 (s, 1H), 6.89 (d, J = 8.1 Hz, 1H), 6.69 (d, J = 8.1 Hz, 1H), 4.54 (t, J = 8.6 Hz, 2H), 3.17 (t, J = 8.6 Hz, 2H), 2.54 (t, J = 7.6 Hz, 2H), 2.35 (t, J = 7.5 Hz, 2H), 1.70 – 1.63 (m, 2H), 1.62 – 1.55 (m, 2H), 1.42 – 1.33 (m, 2H) ppm. ^{13}C NMR (101 MHz, Chloroform-*d*) δ 180.0 , 158.1 , 134.4 , 127.7 , 126.8 , 124.8 , 108.8 , 71.1 , 35.0 , 33.9 , 31.5 , 29.8 , 28.5 , 24.5 ppm. IR(neat): 3440, 2947, 2926, 2883, 2854, 1694, 1639, 1494, 1408, 1291, 1187, 941, 749, 549, 422 cm^{-1}. HRMS: calcd for $C_{14}H_{19}O_3$ $[M+H]^+$ 235.1334, found 235.1333.

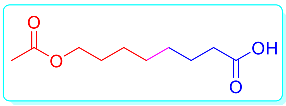

8-acetoxyoctanoic acid (4bc). The reaction was performed following the General Procedure with Ni(COD)$_2$ (8.3 mg, 0.03 mmol), bipy (7.0 mg, 0.045 mmol), **1b** (51.3 mg, 0.45 mmol), Zn powder (39.2 mg, 0.6 mmol), and 4-bromobutyl acetate (**2c**) (43.4 μL, 0.3 mmol). The crude product was purified by flash chromatography on silica gel (eluted with Petroleum ether:EtOAc = 5:1) to give the product (44.9 mg, 74% yield) as a colorless oil. R_f = 0.57 (Petroleum ether:EtOAc = 2:1). ^{1}H NMR (400 MHz, $CDCl_3$) δ: 4.05 (t, *J* = 6.7 Hz, 2H), 2.35 (t, *J* = 7.5 Hz, 2H), 2.05 (s, 3H), 1.66 – 1.59 (m, 4H), 1.38 – 1.34 (m, 6H) ppm. ^{13}C NMR (101 MHz, Chloroform-*d*) δ 179.8 , 171.3 , 64.5 , 33.9 , 28.9 , 28.8 , 28.5 , 25.7 , 24.5 , 20.9 ppm. IR(neat): 3459, 2936, 2859, 1738, 1713, 1651, 1463, 1392, 1367, 1242, 1040, 898, 727, 608, 490 cm^{-1}. HRMS: calcd for $C_{10}H_{19}O_4$ $[M+H]^+$ 203.1283, found 203.1284.

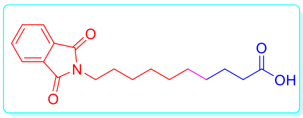

10-(1,3-dioxoisoindolin-2-yl)decanoic acid (4bd). The reaction was performed following the General Procedure with Ni(COD)$_2$ (8.3 mg, 0.03 mmol), bipy (7.0 mg, 0.045 mmol), **1b** (51.3 mg, 0.45 mmol), Zn powder (39.2 mg, 0.6 mmol), and *N*-(6-bromohexyl)phthalimide (**2d**) (93.1 mg, 0.3 mmol). The crude product was purified by flash chromatography on silica gel (eluted with Petroleum ether:EtOAc = 5:1) to give the product (85.7 mg, 90% yield) as a white solid. Mp: 97.3 – 98.9 ºC. R_f = 0.40 (Petroleum ether:EtOAc = 2:1). ^{1}H NMR (400 MHz, $CDCl_3$) δ: 7.85 – 7.83 (m, 2H), 7.72 – 7.70 (m, 2H), 3.68 (t, *J* = 7.3 Hz, 2H), 2.34 (t, *J* = 7.5 Hz, 2H), 1.69 – 1.60 (m, 4H), 1.33 – 1.29 (m, 10H) ppm. ^{13}C NMR (101 MHz, Chloroform-*d*) δ 179.8 , 168.5 , 133.8 , 132.1 , 123.1 , 37.9 , 33.9 , 29.2 , 29.1 , 29.0 , 28.9 , 28.5 , 26.7 , 24.6 ppm. IR(neat): 3467, 2930, 2849, 1772, 1707, 1701, 1616, 1465, 1396, 1252, 1187, 1054, 720, 711, 530 cm^{-1}. HRMS: calcd for $C_{18}H_{24}NO_4$ $[M+H]^+$ 318.1705, found 318.1701.

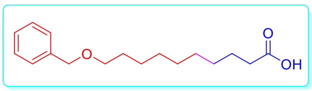

10-(benzyloxy)decanoic acid (4be). The reaction was performed following the General Procedure with Ni(COD)$_2$ (8.3 mg, 0.03 mmol), bipy (7.0 mg, 0.045 mmol), **1b** (51.3 mg, 0.45 mmol), Zn powder (39.2 mg, 0.6 mmol), and 6-benzyloxyhexyl bromide (**2e**) (81.4 mg, 0.3 mmol). The crude product was purified by flash chromatography on silica gel (eluted with Petroleum ether:EtOAc = 5:1) to give the product (56.8 mg, 68% yield) as a white solid. Mp: 30.1 – 31.0 ^{o}C. R_f = 0.63 (Petroleum ether:EtOAc = 2:1). ^{1}H NMR (400 MHz, $CDCl_3$) δ: 7.34 – 7.33 (m, 4H), 7.30 – 7.25 (m, 1H), 4.50 (s, 2H), 3.46 (t, *J* = 6.6 Hz, 2H), 2.34 (t, *J* = 7.5 Hz, 2H), 1.64 – 1.57 (m, 4H), 1.34 – 1.26 (m, 10H) ppm. ^{13}C{^{1}H} NMR (101 MHz, $CDCl_3$) δ: 180.0 , 138.6 , 128.3 , 127.6 , 127.4 , 72.8 , 70.4 , 34.0 , 29.7 , 29.34 , 29.31 , 29.1 , 29.0 , 26.1 , 24.6 ppm. IR(neat): 3651, 3139, 1681, 1496, 1455, 1410, 1361, 1097, 802, 737, 698, 472, 441, 421 cm^{-1}. HRMS: calcd for $C_{17}H_{27}O_3$ $[M+H]^+$ 279.1960, found 279.1961.

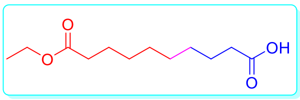

10-ethoxy-10-oxodecanoic acid (4bf). The reaction was performed following the General Procedure with Ni(COD)$_2$ (8.3 mg, 0.03 mmol), bipy (7.0 mg, 0.045 mmol), **1b** (51.3 mg, 0.45 mmol), Zn powder (39.2 mg, 0.6 mmol), and ethyl 6-bromohexanoate (**2f**) (53.4 μL, 0.3 mmol). The crude product was purified by flash chromatography on silica gel (eluted with Petroleum ether:EtOAc = 5:1) to give the product (49.7 mg, 72% yield) as a colorless oil. R_f = 0.62 (Petroleum ether:EtOAc = 2:1). ^{1}H NMR (400 MHz, $CDCl_3$) δ: 4.11 (q, *J* = 7.1 Hz, 2H), 2.33 (t, *J* = 7.5 Hz, 2H), 2.27 (t, *J* = 7.5 Hz, 2H), 1.63 – 1.58 (m, 4H), 1.40 – 1.38 (m, 8H), 1.24 (t, *J* = 7.1 Hz, 3H) ppm. ^{13}C{^{1}H} NMR (101 MHz, $CDCl_3$) δ: 179.9 , 173.9 , 60.2 , 34.3 , 34.0 , 29.0 , 28.9 , 24.9 , 24.6 , 14.2 ppm. Two resonances were not observed due

to overlapping resonances. IR(neat): 3688, 2856, 1732, 1714, 1470, 1302, 1176, 1024, 932, 861, 753, 677, 523, 425 cm^{-1}. HRMS: calcd for $C_{12}H_{23}O_4$ $[M+H]^+$ 231.1596, found 231.1596.

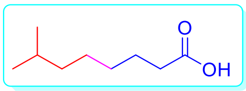

7-methyloctanoic acid (4bh). The reaction was performed following the General Procedure with $Ni(COD)_2$ (8.3 mg, 0.03 mmol), bipy (7.0 mg, 0.045 mmol), **1b** (51.3 mg, 0.45 mmol), Zn powder (39.2 mg, 0.6 mmol), and 1-bromo-3-methylbutane (**2h**) (35.9 μL, 0.3 mmol). The crude product was purified by flash chromatography on silica gel (eluted with Petroleum ether:EtOAc = 5:1) to give the product (29.9 mg, 63% yield) as a colorless oil. R_f = 0.88 (Petroleum ether:EtOAc = 2:1). ^1H NMR (400 MHz, $CDCl_3$) δ: 2.35 (t, J = 7.5 Hz, 2H), 1.68 – 1.60 (m, 2H), 1.55 – 1.48 (m, 1H), 1.33 – 1.29 (m, 4H), 1.19 – 1.15 (m, 2H), 0.86 (d, J = 6.6 Hz, 6H) ppm. $^{13}C\{^1H\}$ NMR (101 MHz, $CDCl_3$) δ:164.5, 38.7 , 33.9 , 29.3 , 27.9 , 26.9 , 24.7 , 22.6 ppm. IR(neat): 3430, 2955, 2930, 2869, 1712, 1467, 1412, 1276, 1232, 939, 764, 749, 596, 437 cm^{-1}. HRMS: calcd for $C_9H_{19}O_2$ $[M+H]^+$ 159.1385, found 159.1385.

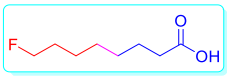

8-fluorooctanoic acid (4bi). The reaction was performed following the General Procedure with $Ni(COD)_2$ (8.3 mg, 0.03 mmol), bipy (7.0 mg, 0.045 mmol), **1b** (51.3 mg, 0.45 mmol), Zn powder (39.2 mg, 0.6 mmol), and 1-bromo-4-fluorobutane (**2i**) (32.2 μL, 0.3 mmol). The crude product was purified by flash chromatography on silica gel (eluted with Petroleum ether:EtOAc = 5:1) to give the product (39.4 mg, 81% yield) as a colorless oil. R_f = 0.68 (Petroleum ether:EtOAc = 2:1). ^1H NMR (400 MHz, $CDCl_3$) δ: 4.50 (t, J = 6.1 Hz, 1H), 4.38 (t, J = 6.1 Hz, 1H), 2.36 (t, J = 7.5 Hz, 2H), 1.74 – 1.70 (m, 1H), 1.69 – 1.61 (m, 3H), 1.45 – 1.34 (m, 6H) ppm. ^{13}C NMR (101 MHz, Chloroform-*d*) δ 177.5, 84.1 (d, $J^1_{C\text{-}F}$ = 164.0 Hz), 33.9 , 30.3 (d, $J^2_{C\text{-}F}$ = 19.4 Hz), 28.9 , 28.8 , 24.9 (d, $J^3_{C\text{-}F}$ = 5.5 Hz), 24.5 ppm. ^{19}F NMR (376 MHz, Chloroform-*d*): δ= −218.0 (s, 1F). IR(neat): 3440, 2936, 1861, 1705, 1646, 1469, 1292, 1099, 1043, 984, 917, 739, 595, 501, 449 cm^{-1}. HRMS: calcd for $C_8H_{16}FO_2$ $[M+H]^+$ 163.1134, found 163.1133.

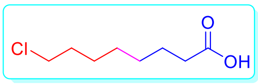

8-chlorooctanoic acid (4bj). The reaction was performed following the General Procedure with $Ni(COD)_2$ (8.3 mg, 0.03 mmol), bipy (7.0 mg, 0.045 mmol), **1b** (51.3 mg, 0.45 mmol), Zn powder (39.2 mg, 0.6 mmol), and 1-bromo-4-chlorobutane (**2j**) (34.6 μL, 0.3 mmol). The crude product was purified by flash chromatography on silica gel (eluted with Petroleum ether:EtOAc = 5:1) to give the product (45.0 mg, 84% yield) as a colorless oil. R_f = 0.68 (Petroleum ether:EtOAc = 2:1). ^1H NMR (400 MHz, $CDCl_3$) δ: 3.53 (t, J = 6.8 Hz, 2H), 2.36 (t, J = 7.5 Hz, 2H), 1.80 – 1.73 (m, 2H), 1.68 – 1.61 (m, 2H), 1.48 – 1.41 (m, 2H), 1.36 – 1.33 (m, 4H) ppm. ^{13}C NMR (101 MHz, Chloroform-*d*) δ 180.2 , 45.0 , 33.9 , 32.5 , 28.8 , 28.5 , 26.6 , 24.5 ppm. IR(neat): 3462, 2936, 2859, 1713, 1455, 1412, 1283, 1247, 1093, 939, 727, 651, 480 cm^{-1}. HRMS: calcd for $C_8H_{16}ClO_2$ $[M+H]^+$ 179.0839, found 179.0843.

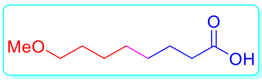

8-methoxyoctanoic acid (4bk). The reaction was performed following the General Procedure with $Ni(COD)_2$ (8.3 mg, 0.03 mmol), bipy (7.0 mg, 0.045 mmol), **1b** (51.3 mg, 0.45 mmol), Zn powder (39.2 mg, 0.6 mmol), and 1-bromo-4-methoxybutane (**2k**) (38.4 μL, 0.3 mmol). The crude product was purified by flash chromatography on silica gel (eluted with Petroleum ether:EtOAc = 5:1) to give the product (45.0 mg, 86% yield) as a colorless oil. R_f = 0.68 (Petroleum ether:EtOAc = 2:1). ^1H NMR (400 MHz, $CDCl_3$) δ: δ 3.37 (t, J = 6.6 Hz, 2H), 3.33 (s, 3H), 2.34 (t, J = 7.4 Hz, 2H), 1.65 – 1.60 (m, 2H), 1.58 – 1.53 (m, 2H), 1.37 – 1.26 (m, 6H) ppm. ^{13}C NMR (101 MHz, Chloroform-*d*) δ 179.6 , 72.8 , 58.5 , 33.9 , 29.5 ,

29.0 , 28.9 , 25.9 , 24.6 ppm. IR(neat): 3426, 2934, 2859, 1737, 1713, 1462, 1412, 1199, 1119, 942, 846, 727, 617, 496 cm^{-1}. HRMS: calcd for $C_9H_{19}O_3$ $[M+H]^+$ 175.1334, found 175.1330.

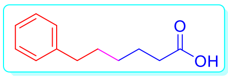

6-phenylhexanoic acid (4bl). The reaction was performed following the General Procedure with $Ni(COD)_2$ (8.3 mg, 0.03 mmol), bipy (7.0 mg, 0.045 mmol), **1b** (51.3 mg, 0.45 mmol), Zn powder (39.2 mg, 0.6 mmol), and (2-bromoethyl)benzene (**2l**) (41.0 µL, 0.3 mmol). The crude product was purified by flash chromatography on silica gel (eluted with Petroleum ether:EtOAc = 5:1) to give the product (51.9 mg, 90% yield) as a colorless oil. R_f = 0.73 (Petroleum ether:EtOAc = 2:1). ^{1}H NMR (400 MHz, $CDCl_3$) δ: 7.29 – 7.24 (m, 2H), 7.19 – 7.16 (m, 3H), 2.61 (t, J = 7.6 Hz, 2H), 2.35 (t, J = 7.5 Hz, 2H), 1.72 – 1.60 (m, 4H), 1.43 – 1.35 (m, 2H) ppm. ^{13}C NMR (101 MHz, Chloroform-d) δ 180.1 , 142.4 , 128.4 , 128.3 , 125.7 , 35.7 , 33.9 , 31.1 , 28.6 , 24.5 ppm. IR(neat): 3412, 3085, 3027, 2934, 2858, 1709, 1496, 1453, 1288, 1225, 938, 747, 699,493 cm^{-1}. HRMS: calcd for $C_{12}H_{17}O_2$ $[M+H]^+$ 193.1229, found 193.1229.

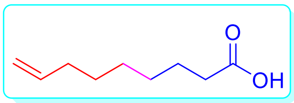

non-8-enoic acid (4bm) . The reaction was performed following the General Procedure with $Ni(COD)_2$ (8.3 mg, 0.03 mmol), bipy (7.0 mg, 0.045 mmol), **1b** (51.3 mg, 0.45 mmol), Zn powder (39.2 mg, 0.6 mmol), and 5-bromo-1-pentene (**2m**) (35.5 µL, 0.3 mmol). The crude product was purified by flash chromatography on silica gel (eluted with Petroleum ether:EtOAc = 5:1) to give the product (31.4 mg, 67% yield) as a colorless oil. R_f = 0.70 (Petroleum ether:EtOAc = 2:1). ^{1}H NMR (400 MHz, $CDCl_3$) δ: 5.85 – 5.75 (m, 1H), 5.02 – 4.92 (m, 2H), 2.35 (t, J = 7.5 Hz, 2H), 2.07 – 2.02 (m, 2H), 1.67 – 1.60 (m, 2H), 1.41 – 1.29 (m, 6H) ppm. ^{13}C NMR (101 MHz, Chloroform-d) δ: 180.3 , 138.9 , 114.3 , 34.0 , 33.7 , 28.8 , 28.65 , 28.63 , 24.6 ppm. IR(neat): 3042, 2919, 2874, 1709, 1688, 1639, 1605, 1479, 1447, 1301, 1156, 1025, 980, 831, 755, 626 cm^{-1}. HRMS: calcd for $C_9H_{17}O_2$ $[M+H]^+$ 157.1229, found 157.1226.

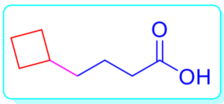

4-cyclobutylbutanoic acid (4bp). The reaction was performed following the General Procedure with $Ni(COD)_2$ (8.3 mg, 0.03 mmol), bipy (7.0 mg, 0.045 mmol), **1b** (51.3 mg, 0.45 mmol), Zn powder (39.2 mg, 0.6 mmol), and cyclobutyl bromide (**2p**) (28.2 µL, 0.3 mmol). The crude product was purified by flash chromatography on silica gel (eluted with Petroleum ether:EtOAc = 5:1) to give the product (26.4 mg, 62% yield) as a colorless oil. R_f = 0.78 (Petroleum ether:EtOAc = 2:1). ^{1}H NMR (400 MHz, $CDCl_3$) δ: 2.33 (t, J = 7.4 Hz, 2H), 2.29 – 2.21 (m, 1H), 2.07 – 2.00 (m, 2H), 1.89 – 1.76 (m, 2H), 1.61 – 1.51 (m, 4H), 1.45 – 1.39 (m, 2H) ppm. ^{13}C NMR (101 MHz, Chloroform-d) δ 177.9 , 36.2 , 35.7 , 33.9 , 28.2 , 22.4 , 18.4 ppm. IR(neat): 3484, 2934, 2864, 1709, 1442, 1412, 1242, 1120, 933, 914, 748, 596, 488, 465 cm^{-1}. HRMS: calcd for $C_8H_{15}O_2$ $[M+H]^+$ 143.1072, found 143.1072.

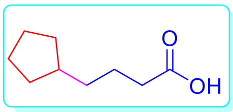

4-cyclopentylbutanoic acid (4bn). The reaction was performed following the General Procedure with $Ni(COD)_2$ (8.3 mg, 0.03 mmol), bipy (7.0 mg, 0.045 mmol), **1b** (51.3 mg, 0.45 mmol), Zn powder (39.2 mg, 0.6 mmol), and bromocyclopentane (**2n**) (32.2 µL, 0.3 mmol). The crude product was purified by flash chromatography on silica gel (eluted with Petroleum ether:EtOAc = 5:1) to give the product (38.9 mg, 83% yield) as a colorless oil. R_f = 0.76 (Petroleum ether:EtOAc = 2:1). ^{1}H NMR (400 MHz, $CDCl_3$) δ: 2.35 (t, J = 7.5 Hz, 2H), 1.76 – 1.72 (m, 3H), 1.68 – 1.58 (m, 4H), 1.52 – 1.50 (m, 2H), 1.37 – 1.32 (m, 2H), 1.10 – 1.05 (m, 2H) ppm. ^{13}C NMR (101 MHz, Chloroform-d) δ 180.1 , 39.8 , 35.5 , 34.3 , 32.6 , 25.1 , 23.9 ppm. IR(neat): 3426, 2949, 2868, 1709, 1453, 1413, 1293, 1211, 1124, 935,

763, 656, 600, 495 cm^{-1}. HRMS: calcd for $C_9H_{17}O_2$ $[M+H]^+$ 157.1224, found 157.1229.

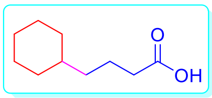

4-cyclohexylbutanoic acid (4bo). The reaction was performed following the General Procedure with $Ni(COD)_2$ (8.3 mg, 0.03 mmol), bipy (7.0 mg, 0.045 mmol), **1b** (51.3 mg, 0.45 mmol), Zn powder (39.2 mg, 0.6 mmol), and bromocyclohexane (**2o**) (36.9 µL, 0.3 mmol). The crude product was purified by flash chromatography on silica gel (eluted with Petroleum ether:EtOAc = 5:1) to give the product (38.8 mg, 76% yield) as a colorless oil. R_f = 0.70 (Petroleum ether:EtOAc = 2:1). The spectroscopic data for this product match the literature data[5].

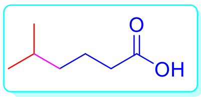

5-methylhexanoic acid (4bq). The reaction was performed following the General Procedure with $Ni(COD)_2$ (8.3 mg, 0.03 mmol), bipy (7.0 mg, 0.045 mmol), **1b** (51.3 mg, 0.45 mmol), Zn powder (39.2 mg, 0.6 mmol), and 2-bromopropane (**2q**) (28.2 µL, 0.3 mmol). The crude product was purified by flash chromatography on silica gel (eluted with Petroleum ether:EtOAc = 5:1) to give the product (22.3 mg, 57% yield) as a colorless oil. R_f = 0.77 (Petroleum ether:EtOAc = 2:1). ^{1}H NMR (400 MHz, $CDCl_3$) δ: 2.34 (t, J = 7.6 Hz, 2H), 1.65 – 1.56 (m, 2H), 1.25 – 1.21 (m, 2H), 1.10 – 1.05 (m, 1H), 0.89 (d, J = 6.6 Hz, 6H) ppm. ^{13}C NMR (101 MHz, Chloroform-*d*) δ 179.9 , 38.2 , 34.4 , 27.7 , 22.6 , 22.4 ppm. IR(neat): 3427, 2956, 2933, 2872, 1710, 1486, 1412, 1262, 1109, 946, 752, 655, 632, 465 cm^{-1}. HRMS: calcd for $C_7H_{15}O_2$ $[M+H]^+$ 131.1072, found 131.1073.

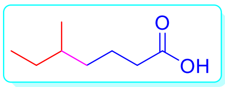

5-methylheptanoic acid (4br). The reaction was performed following the General Procedure with $Ni(COD)_2$ (8.3 mg, 0.03 mmol), bipy (7.0 mg, 0.045 mmol), **1b** (51.3 mg, 0.45 mmol), Zn powder (39.2 mg, 0.6 mmol), and 2-bromobutane (**2r**) (41.1 mg, 0.3 mmol). The crude product was purified by flash chromatography on silica gel (eluted with Petroleum ether:EtOAc = 5:1) to give the product (24.2 mg, 56% yield) as a colorless oil. R_f = 0.74 (Petroleum ether:EtOAc = 2:1). ^{1}H NMR (400 MHz, Chloroform-*d*) δ: 2.34 (t, J = 7.8 Hz, 2H), 1.72 – 1.56 (m, 2H), 1.38 – 1.29 (m, 3H), 1.21 – 1.10 (m, 2H), 0.88 – 0.84 (m, 6H) ppm. ^{13}C NMR (101 MHz, Chloroform-*d*) δ 180.2 , 35.9 , 34.4 , 34.1 , 29.3 , 22.3 , 19.0 , 11.3 ppm. IR(neat): 3426, 2959, 2927, 2856, 1711, 1463, 1412, 1286, 1115, 938, 750, 597, 496, 477 cm^{-1}. HRMS: calcd for $C_8H_{17}O_2$ $[M+H]^+$ 145.1229, found 145.1231.

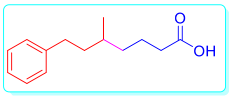

5-methyl-7-phenylheptanoic acid (4bs). The reaction was performed following the General Procedure with $Ni(COD)_2$ (8.3 mg, 0.03 mmol), bipy (7.0 mg, 0.045 mmol), **1b** (51.3 mg, 0.45 mmol), Zn powder (39.2 mg, 0.6 mmol), and (3-bromobutyl)benzene (**2s**) (64.0 mg, 0.3 mmol). The crude product was purified by flash chromatography on silica gel (eluted with Petroleum ether:EtOAc = 5:1) to give the product (46.9 mg, 71% yield) as a colorless oil. R_f = 0.71 (Petroleum ether:EtOAc = 2:1). ^{1}H NMR (400 MHz, Chloroform-*d*) δ 7.28 – 7.24 (m, 2H), 7.18-7.16 (m, 3H), 2.68 – 2.60 (m, 1H), 2.59 – 2.53 (m, 1H), 2.33 (t, J = 7.2 Hz, 2H), 1.68 – 1.60 (m, 3H), 1.46 – 1.36 (m, 3H), 1.25 – 1.18 (m, 1H), 0.94 (d, J = 5.6 Hz, 3H) ppm. ^{13}C NMR (101 MHz, Chloroform-*d*) δ 142.9 , 128.28 , 128.26, 125.6 , 38.7 , 36.2 , 33.4 , 32.2 , 22.2 , 19.4 ppm. Two resonances were not observed due to overlapping resonances. IR(neat): 3427, 3026, 2953, 2929, 2870, 1708, 1496, 1454, 1275, 1097, 939, 746, 699, 514 cm^{-1}. HRMS: calcd for $C_{14}H_{21}O_2$ $[M+H]^+$ 221.1542, found 221.1539.

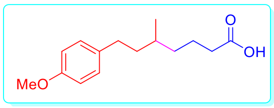

7-(4-methoxyphenyl)-5-methylheptanoic acid (4bt). The reaction was performed following the General Procedure with Ni(COD)$_2$ (8.3 mg, 0.03 mmol), bipy (7.0 mg, 0.045 mmol), **1b** (51.3 mg, 0.45 mmol), Zn powder (39.2 mg, 0.6 mmol), and 1-(3-bromobutyl)-4-methoxybenzene (**2t**) (73.0 mg, 0.3 mmol). The crude product was purified by flash chromatography on silica gel (eluted with Petroleum ether:EtOAc = 5:1) to give the product (50.3 mg, 67% yield) as a colorless oil. R_f = 0.67 (Petroleum ether:EtOAc = 2:1). ^{1}H NMR (400 MHz, Chloroform-*d*) δ:7.09 (d, *J* = 8.7 Hz, 2H), 6.82 (d, *J* = 8.7 Hz, 2H), 3.78 (s, 3H), 2.63 – 2.45 (m, 2H), 2.35 – 2.32 (m, 2H), 1.72 – 1.55 (m, 3H), 1.48 – 1.34 (m, 3H), 1.25 – 1.16 (m, 1H), 0.93 (d, *J* = 6.3 Hz, 3H) ppm. ^{13}C NMR (101 MHz, Chloroform-*d*) δ 157.6 , 134.9 , 129.2 , 113.7 , 55.2 , 38.9 , 36.2 , 32.4 , 32.1 , 22.2 , 19.4 ppm. Two resonances were not observed due to overlapping resonances. IR(neat): 3426, 2952, 2930, 2869, 1708, 1612, 1512, 1463, 1245, 1177, 1037, 822, 655, 561, 523 cm^{-1}. HRMS: calcd for $C_{15}H_{23}O_3$ [M+H]$^+$ 251.1647, found 251.1648.

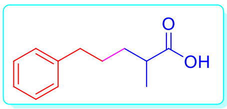

2-methyl-5-phenylpentanoic acid (5cl) The reaction was performed following the General Procedure with Ni(COD)$_2$ (8.3 mg, 0.03 mmol), bipy (7.0 mg, 0.045 mmol), **1c** (51.3 mg, 0.45 mmol), Zn powder (39.2 mg, 0.6 mmol), and (2-bromoethyl)benzene (**2l**) (41.0 µL, 0.3 mmol). The crude product was purified by flash chromatography on silica gel (eluted with Petroleum ether:EtOAc = 5:1) to give the product (39.2 mg, 68% yield) as a colorless oil. R_f = 0.72 (Petroleum ether:EtOAc = 2:1). ^{1}H NMR (400 MHz, Chloroform-*d*) δ: 7.29 – 7.25 (m, 2H), 7.20 – 7.16 (m, 3H), 2.62 (t, *J* = 7.5 Hz, 2H), 2.53 – 2.45 (m, 1H), 1.76 – 1.62 (m, 3H), 1.52 – 1.46 (m, 1H), 1.18 (d, *J* = 7.0 Hz, 3H) ppm. ^{13}C NMR (101 MHz, Chloroform-*d*) δ:182.8 , 142.1 , 128.4 , 128.3 , 125.8 , 39.2 , 35.7 , 33.1 , 28.9 , 16.8 ppm. IR(neat): 3439, 3029, 2979, 2942, 2861, 1693, 1647, 1496, 1416, 1238, 1081, 926, 747, 698, 541 cm^{-1}. HRMS: calcd for $C_{12}H_{16}O_2$ [M+H]$^+$ 193.1229, found 193.1230.

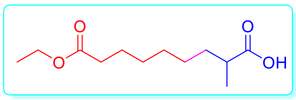

9-ethoxy-2-methyl-9-oxononanoic acid (5cf) The reaction was performed following the General Procedure with Ni(COD)$_2$ (8.3 mg, 0.03 mmol), bipy (7.0 mg, 0.045 mmol), **1c** (51.3 mg, 0.45 mmol), Zn powder (39.2 mg, 0.6 mmol), and ethyl 6-bromohexanoate (**2f**) (53.4 µL, 0.3 mmol). The crude product was purified by flash chromatography on silica gel (eluted with Petroleum ether:EtOAc = 5:1) to give the product (52.5 mg, 76% yield) as a colorless oil. R_f = 0.65 (Petroleum ether:EtOAc = 2:1). ^{1}H NMR (400 MHz, Chloroform-*d*) δ: 4.13 (q, *J* = 7.1 Hz, 2H), 2.48 – 2.43 (m, 1H), 2.29 (t, *J* = 7.5 Hz, 2H), 1.71 – 1.59 (m, 3H), 1.45 – 1.38 (m, 7H), 1.26 (t, *J* = 7.2 Hz, 3H), 1.18 (d, *J* = 7.0 Hz, 3H) ppm. ^{13}C NMR (101 MHz, Chloroform-*d*) δ: 182.6 , 173.9 , 60.2 , 39.3 , 34.3 , 33.4 , 29.1 , 28.9 , 26.9 , 24.8 , 16.8 , 14.2 ppm. IR(neat): 3454, 2978, 2859, 1736, 1706, 1639, 1465, 1374, 1184, 1096, 786, 726, 621, 545 cm^{-1}. HRMS: calcd for $C_{12}H_{22}O_4$ [M+H]$^+$ 231.1596 , found 231.1595.

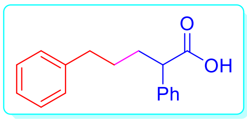

2,5-diphenylpentanoic acid (6dl) The reaction was performed following the General Procedure with Ni(COD)$_2$ (8.3 mg, 0.03 mmol), bipy (7.0 mg, 0.045 mmol), **1d** (79.2 mg, 0.45 mmol), Zn powder (39.2 mg, 0.6 mmol), and (2-bromoethyl)benzene (**2l**) (41.0 µL, 0.3 mmol). The crude product was purified by flash chromatography on silica gel (eluted with Petroleum ether:EtOAc = 5:1) to give the product (62.6 mg, 82% yield) as a colorless oil. R_f = 0.69 (Petroleum ether:EtOAc = 2:1). ^{1}H NMR (400 MHz, Chloroform-*d*) δ: 7.31 – 7.23 (m, 7H), 7.18 – 7.17 (m, 1H), 7.13 – 7.11 (m, 2H), 3.54 (t, *J* =

7.5 Hz, 1H), 2.67 – 2.54 (m, 2H), 2.09 – 2.05 (m, 1H), 1.88 – 1.79 (m, 1H), 1.66 – 1.52 (m, 2H) ppm. ^{13}C NMR (101 MHz, Chloroform-*d*) δ: 179.6, 141.8 , 138.3 , 128.6 , 128.3 , 128.3 , 128.0 , 127.4 , 125.8 , 51.4 , 35.5 , 32.6 , 29.2 ppm. IR(neat): 3426, 2952, 2930, 2869, 1636, 1496, 1453, 1407, 1367, 1260, 1155, 1091, 1029, 698, 556, 441, 421 cm^{-1}. HRMS: calcd for $C_{17}H_{18}O_2$ $[M+H]^+$ 255.1385, found 255.1385.

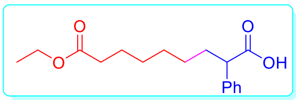

9-ethoxy-9-oxo-2-phenylnonanoic acid (6df) The reaction was performed following the General Procedure with Ni(COD)$_2$ (8.3 mg, 0.03 mmol), bipy (7.0 mg, 0.045 mmol), **1d** (79.2 mg, 0.45 mmol), Zn powder (39.2 mg, 0.6 mmol), and ethyl 6-bromohexanoate (**2f**) (53.4 µL, 0.3 mmol). The crude product was purified by flash chromatography on silica gel (eluted with Petroleum ether:EtOAc = 5:1) to give the product (70.2 mg, 80% yield) as a colorless oil. R$_f$ = 0.62 (Petroleum ether:EtOAc = 2:1). ^{1}H NMR (400 MHz, Chloroform-*d*) δ: 7.34 – 7.31 (m, 4H), 7.22 – 7.19 (m, 1H), 4.11 (q, *J* = 7.1 Hz, 2H), 3.53 (t, *J* = 7.7 Hz, 1H), 2.26 (t, *J* = 7.5 Hz, 2H), 2.07 – 2.01 (m, 1H), 1.78 – 1.76 (m, 1H), 1.61 – 1.56 (m, 2H), 1.32 – 1.20 (m, 9H) ppm. ^{13}C NMR (101 MHz, Chloroform-*d*) δ: 179.9 , 173.9 , 138.5 , 128.6 , 127.9 , 127.4 , 60.2 , 51.5 , 34.2 , 32.9 , 28.9 , 28.8 , 27.2 , 24.8 , 14.2 ppm. IR(neat): 3454, 3031, 2983, 2937, 2859, 1701, 1643, 1496, 1455, 1373, 1180, 1031, 750, 699, 649 cm^{-1}. HRMS: calcd for $C_{17}H_{24}O_4$ $[M+H]^+$ 293.1753, found 293.1755.

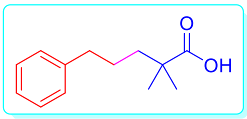

2,2-dimethyl-5-phenylpentanoic acid (7el) The reaction was performed following the General Procedure with Ni(COD)$_2$ (8.3 mg, 0.03 mmol), bipy (7.0 mg, 0.045 mmol), **1e** (57.6 mg, 0.45 mmol), Zn powder (39.2 mg, 0.6 mmol), and (2-bromoethyl)benzene (**2l**) (41.0 µL, 0.3 mmol). The crude product was purified by flash chromatography on silica gel (eluted with Petroleum ether:EtOAc = 5:1) to give the product (38.4 mg, 62% yield) as a colorless oil. R$_f$ = 0.72 (Petroleum ether:EtOAc = 2:1). ^{1}H NMR (400 MHz, Chloroform-*d*) δ: 7.30 – 7.26 (m, 2H), 7.22 – 7.16 (m, 3H), 2.60 (t, *J* = 6.7 Hz, 2H), 1.65 – 1.57 (m, 4H), 1.18 (s, 6H) ppm. ^{13}C NMR (101 MHz, Chloroform-*d*) δ: 184.5 , 142.2 , 128.33 , 128.28 , 125.7 , 42.0 , 40.1 , 36.2 , 26.7 , 24.9 ppm. IR(neat): 3447, 3028, 2977, 2944, 1698, 1639, 1475, 1409, 1369, 1277, 1199, 1031, 939, 748, 698, 567 cm^{-1}. HRMS: calcd for $C_{13}H_{18}O_2$ $[M+H]^+$ 207.1387, found 207.1383.

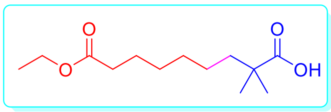

9-ethoxy-2,2-dimethyl-9-oxononanoic acid (7ef) The reaction was performed following the General Procedure with Ni(COD)$_2$ (8.3 mg, 0.03 mmol), bipy (7.0 mg, 0.045 mmol), **1e** (57.6 mg, 0.45 mmol), Zn powder (39.2 mg, 0.6 mmol), and ethyl 6-bromohexanoate (**2f**) (53.4 µL, 0.3 mmol). The crude product was purified by flash chromatography on silica gel (eluted with Petroleum ether:EtOAc = 5:1) to give the product (60.8 mg, 83% yield) as a colorless oil. R$_f$ = 0.66 (Petroleum ether:EtOAc = 2:1). ^{1}H NMR (400 MHz, Chloroform-*d*) δ: 4.13 (q, *J* = 7.1 Hz, 2H), 2.29 (t, *J* = 7.5 Hz, 2H), 1.63 – 1.59 (m, 2H), 1.54 – 1.49 (m, 2H), 1.35 – 1.22 (m, 9H), 1.18 (s, 6H) ppm. ^{13}C NMR (101 MHz, Chloroform-*d*) δ: 184.4 , 173.9 , 60.2 , 42.0 , 40.4 , 34.3 , 29.8 , 28.9 , 24.91 , 24.89 , 24.6 , 14.2 ppm. IR(neat): 3451, 3028, 2978, 2977, 1694, 1639, 1496, 1454, 1367, 1241, 1156, 1030, 941, 749, 700, 432 cm^{-1}. HRMS: calcd for $C_{13}H_{24}O_4$ $[M+H]^+$ 245.1753, found 245.1754.

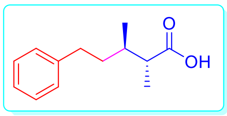

2,3-dimethyl-5-phenylpentanoic acid (8fl) The reaction was performed following the General Procedure with Ni(COD)$_2$ (8.3 mg, 0.03 mmol), bipy (7.0 mg, 0.045 mmol), **1f** (57.6 mg, 0.45 mmol), Zn powder (39.2 mg, 0.6 mmol), and (2-bromoethyl)benzene (**2l**) (41.0 µL, 0.3 mmol). The crude

product was purified by flash chromatography on silica gel (eluted with Petroleum ether:EtOAc = 5:1) to give the product (37.1 mg, 60% yield) as a colorless oil. R_f = 0.73 (Petroleum ether:EtOAc = 2:1). ^{1}H NMR (400 MHz, Chloroform-*d*) δ: 7.29 – 7.23 (m, 2H), 7.20 – 7.15 (m, 3H), 2.69 – 2.66 (m, 1H), 2.58 – 2.55 (m, 1H), 2.51 – 2.46 (m, 1H), 2.00 – 1.95 (m, 1H), 1.75 – 1.64 (m, 1H), 1.53 – 1.49 (m, 1H), 1.10 (d, *J* = 6.9 Hz, 3H), 0.96 (d, *J* = 6.6 Hz, 3H) ppm. ^{13}C NMR (101 MHz, Chloroform-*d*) δ: 182.6 , 142.3 , 128.34 , 128.30 , 125.7 , 43.8 , 36.6 , 34.8 , 33.6 , 15.5 , 11.7 ppm. IR(neat): 3428, 3027, 2969, 2936, 2861, 2636, 1702, 1643, 1605, 1496, 1384, 1234, 1078, 1030, 939, 748, 698 cm^{-1}. HRMS: calcd for $C_{13}H_{18}O_2$ $[M+H]^+$ 207.1385 , found 207.1388.

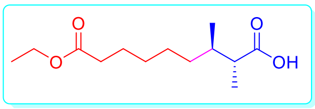

9-ethoxy-2,3-dimethyl-9-oxononanoic acid (8ff) The reaction was performed following the General Procedure with Ni(COD)$_2$ (8.3 mg, 0.03 mmol), bipy (7.0 mg, 0.045 mmol), **1f** (57.6 mg, 0.45 mmol), Zn powder (39.2 mg, 0.6 mmol), and ethyl 6-bromohexanoate (**2f**) (53.4 μL, 0.3 mmol). The crude product was purified by flash chromatography on silica gel (eluted with Petroleum ether:EtOAc = 5:1) to give the product (56.4 mg, 77% yield) as a colorless oil. R_f = 0.70 (Petroleum ether:EtOAc = 2:1). ^{1}H NMR (400 MHz, Chloroform-*d*) δ: 4.13 (q, *J* = 7.1 Hz, 2H), 2.42 – 2.39 (m, 1H), 2.31 – 2.27 (m, 2H), 1.88 – 1.87 (m, 1H), 1.63 – 1.62 (m, 2H), 1.36 – 1.21 (m, 9H), 1.08 (d, *J* = 6.3 Hz, 3H), 0.88 (d, *J* = 6.1 Hz, 3H) ppm. ^{13}C NMR (101 MHz, Chloroform-*d*) δ: 188.1 , 173.9 , 60.2 , 43.8 , 34.9 , 34.4 , 34.3 , 29.2 , 26.8 , 24.9 , 15.5 , 14.2 , 11.7 ppm. IR(neat): 3455, 1732, 1697, 1644, 1475, 1372, 1261, 1032, 938, 853, 726, 595 cm^{-1}. HRMS: calcd for $C_{13}H_{24}O_4$ $[M+H]^+$ 245.1753, found 245.1758.

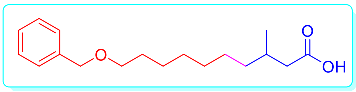

10-(benzyloxy)-3-methyldecanoic acid (9ge). The reaction was performed following the General Procedure with Ni(COD)$_2$ (8.3 mg, 0.03 mmol), bipy (7.0 mg, 0.045 mmol), **1g** (57.7 mg, 0.45 mmol), Zn powder (39.2 mg, 0.6 mmol), and 6-benzyloxyhexyl bromide (**2e**) (81.4 mg, 0.3 mmol). The crude product was purified by flash chromatography on silica gel (eluted with Petroleum ether:EtOAc = 5:1) to give the product (63.2 mg, 72% yield) as a colorless oil. R_f = 0.53 (Petroleum ether:EtOAc = 2:1). ^{1}H NMR (400 MHz, $CDCl_3$) δ: 7.34 – 7.33 (m, 4H), 7.30 – 7.25 (m, 1H), 4.51 (s, 2H), 3.46 (t, *J* = 6.6 Hz, 2H), 2.37 – 2.32 (m, 1H), 2.16 – 2.10 (m, 1H), 1.96 – 1.94 (m, 1H), 1.64 – 1.57 (m, 2H), 1.41 – 1.28 (m, 9H), 1.21 – 1.18 (m, 1H), 0.96 (d, *J* = 6.6 Hz, 3H) ppm. ^{13}C{^{1}H} NMR (101 MHz, $CDCl_3$) δ: 179.5 , 138.6, 128.3 , 127.6 , 127.5 , 72.8 , 70.4 , 41.6 , 36.6 , 30.1 , 29.7 , 29.6 , 29.4 , 26.8 , 26.1 , 19.7 ppm. IR(neat): 3522, 2929, 2855, 2793, 1708, 1603, 1544, 1496, 1408, 1363, 1298, 1102, 936, 906, 878, 734, 698, 524, 462 cm^{-1}. HRMS: calcd for $C_{18}H_{29}O_3$ $[M+H]^+$ 293.2117, found 293.2115.

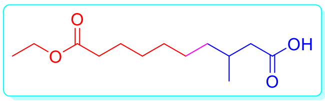

10-ethoxy-3-methyl-10-oxodecanoic acid (9gf). The reaction was performed following the General Procedure with Ni(COD)$_2$ (8.3 mg, 0.03 mmol), bipy (7.0 mg, 0.045 mmol), **1g** (57.7 mg, 0.45 mmol), Zn powder (39.2 mg, 0.6 mmol), and ethyl 6-bromohexanoate (**2f**) (53.4 μL, 0.3 mmol). The crude product was purified by flash chromatography on silica gel (eluted with Petroleum ether:EtOAc = 5:1) to give the product (59.4 mg, 81% yield) as a colorless oil. R_f = 0.51 (Petroleum ether:EtOAc = 2:1). ^{1}H NMR (400 MHz, $CDCl_3$) δ: 4.11 (q, *J* = 7.1 Hz, 2H), 2.36 – 2.26 (m, 3H), 2.15 – 2.10 (m, 1H), 1.94 – 1.93 (m, 1H), 1.62 – 1.57 (m, 2H), 1.30 – 1.22 (m, 11H), 0.95 (d, *J* = 6.6 Hz, 3H) ppm. ^{13}C{^{1}H} NMR (101 MHz, $CDCl_3$) δ: 179.5 , 173.9 , 60.2 , 41.5 , 36.5 , 34.3 , 30.1 , 29.3 , 29.0 , 26.7 , 24.9 , 19.6 , 14.2 ppm. IR(neat): 3467, 2933, 2858, 1736, 1708, 1465, 1374, 1249, 1185, 1034, 975, 901, 860, 725, 612, 524, 496, 419 cm^{-1}. HRMS: calcd for $C_{13}H_{25}O_4$ $[M+H]^+$ 245.1753, found 245.1754.

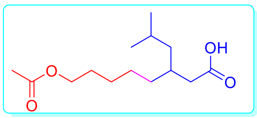

8-acetoxy-3-isobutyloctanoic acid (10hc). The reaction was performed following the General Procedure with Ni(COD)$_2$ (8.3 mg, 0.03 mmol), bipy (7.0 mg, 0.045 mmol), **1h** (76.6 mg, 0.45 mmol), Zn powder (39.2 mg, 0.6 mmol), and 4-bromobutyl acetate (**2c**) (43.4 μL, 0.3 mmol). The crude product was purified by flash chromatography on silica gel (eluted with Petroleum ether:EtOAc = 5:1) to give the product (64.3 mg, 83% yield) as a colorless oil. R_f = 0.59 (Petroleum ether:EtOAc = 2:1). ^{1}H NMR (400 MHz, $CDCl_3$) δ: 4.05 (t, J = 6.7 Hz, 2H), 2.28 – 2.25 (m, 2H), 2.05 (s, 3H), 1.94 – 1.91 (m, 1H), 1.65 – 1.59 (m, 3H), 1.35 – 1.29 (m, 6H), 1.21 – 1.10 (m, 2H), 0.88 (d, J = 6.6 Hz, 6H) ppm. ^{13}C NMR (101 MHz, Chloroform-d) δ: 179.6 , 171.3 , 64.6 , 43.5 , 39.1 , 33.9 , 32.5 , 28.5 , 26.1 , 25.9 , 25.2 , 22.8 , 22.7 , 20.9 ppm. IR(neat): 3452, 2955, 2868, 1739, 1712, 1467, 1367, 1239, 1042, 674, 599, 481, 460, 430 cm^{-1}. HRMS: calcd for $C_{14}H_{27}O_4$ $[M+H]^+$ 259.1909, found 259.1905.

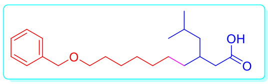

10-(benzyloxy)-3-isobutyldecanoic acid (10he). The reaction was performed following the General Procedure with Ni(COD)$_2$ (8.3 mg, 0.03 mmol), bipy (7.0 mg, 0.045 mmol), **1h** (76.6 mg, 0.45 mmol), Zn powder (39.2 mg, 0.6 mmol), and 6-benzyloxyhexyl bromide (**2e**) (81.4 mg, 0.3 mmol). The crude product was purified by flash chromatography on silica gel (eluted with Petroleum ether:EtOAc = 5:1) to give the product (80.3 mg, 80% yield) as a colorless oil. R_f = 0.57 (Petroleum ether:EtOAc = 2:1). ^{1}H NMR (400 MHz, $CDCl_3$) δ: 7.34 – 7.33 (m, 4H), 7.29 – 7.24 (m, 1H), 4.50 (s, 2H), 3.46 (t, J = 6.6 Hz, 2H), 2.26 – 2.24 (m, 2H), 1.93 – 1.90 (m, 1H), 1.65 – 1.59 (m, 3H), 1.37 – 1.25 (m, 10H), 1.20 – 1.09 (m, 2H), 0.88 (d, J = 6.6 Hz, 6H) ppm. ^{13}C NMR (101 MHz, Chloroform-d) δ: 179.9 , 138.5 , 128.3 , 127.6 , 127.4 , 72.8 , 70.4 , 43.6 , 39.2 , 33.9 , 32.5 , 29.8 , 29.7 , 29.4 , 26.2 , 26.1 , 25.2 , 22.72 , 22.70 ppm. IR(neat): 3428, 3088, 3064, 3031, 2929, 2856, 1705, 1652, 1454, 1366, 1275, 1102, 734, 697, 612 cm^{-1}. HRMS: calcd for $C_{21}H_{35}O_3$ $[M+H]^+$ 335.2586, found 335.2585.

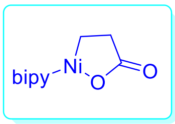

H: To an oven-dried microwave vial (10 mL) equipped with a stir bar (10 × 5 mm) was added Ni(COD)$_2$ (5.0 mmol), bipy (5.0 mmol) and **1a** (5.0 mmol) under an argon atmosphere inside a glove box at 25 °C. Next, dry THF (5.0 mL) was added via syringe to give a purple solution. The reaction mixture was stirred for 6 h to obtain a red precipitate. The complex was filtered, washed with hexane repeatedly and yield 0.76g (53%) of a red product **H.** The spectroscopic data for this product match the literature data[6].

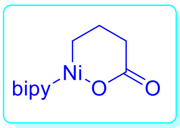

G: To an oven-dried microwave vial (10 mL) equipped with a stir bar (10 × 5 mm) was added Ni(COD)$_2$ (5.0 mmol), bipy (5.0 mmol) and **1b** (5.0 mmol) under an argon atmosphere inside a glove box at 25 °C. Next, dry THF (5.0 mL) was added via syringe to give a purple solution. The reaction mixture was stirred for 6 h to obtain a red precipitate. The complex was filtered, washed with hexane repeatedly and yield 1.23g (82%) of a red product **G.** The spectroscopic data for this product match the literature data[6].

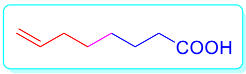

oct-7-enoic acid (4bu'). The reaction was performed following the General Procedure with Ni(COD)$_2$ (8.3 mg, 0.03 mmol), bipy (7.0 mg, 0.045 mmol), **1b** (51.3 mg, 0.45 mmol), Zn powder (39.2 mg, 0.6 mmol), and cyclopropylmethyl bromide (**2u**) (29.1 μL, 0.3 mmol). The crude product was purified by flash chromatography on silica gel (eluted with Petroleum ether:EtOAc = 5:1) to give the product (22.2 mg, 52% yield) as a colorless oil. R_f = 0.82 (Petroleum ether:EtOAc = 2:1). The spectroscopic data for this product match the literature data[7].

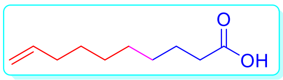

dec-9-enoic acid (4bv). The reaction was performed following the General Procedure with Ni(COD)$_2$ (33.0 mg, 0.12 mmol), bipy (28.0 mg, 0.18 mmol), **1b** (51.3 mg, 0.45 mmol), Zn powder (39.2 mg, 0.6 mmol), and 6-bromo-1-hexene (**2v**) (40.1 μL, 0.3 mmol). The crude product was purified by flash chromatography on silica gel (eluted with Petroleum ether:EtOAc = 5:1) to give the product (33.2 mg, 65% yield) as a colorless oil. R_f = 0.84 (Petroleum ether:EtOAc = 2:1). The spectroscopic data for this product match the literature data[8].

Supplementary References

1 Jung, H.-Y., Chang, S. & Hong, S. Strategic Approach to the Metamorphosis of γ-Lactones to NH γ-Lactams via Reductive Cleavage and C–H Amidation. *Org. Lett.* **21**, 7099-7103 (2019).

2 Ren, W., Chu, J., Sun, F. & Shi, Y. Pd-Catalyzed Highly Chemo- and Regioselective Hydrocarboxylation of Terminal Alkyl Olefins with Formic Acid. *Org. Lett.* **21**, 5967-5970 (2019).

3 Qiu, J. C., Pradhan, P. P., Blanck, N. B., Bobbitt, J. M. & Bailey, W. F. Selective Oxoammonium Salt Oxidations of Alcohols to Aldehydes and Aldehydes to Carboxylic Acids. *Org. Lett.* **14**, 350-353 (2012).

4 Jiang, X., Zhang, J. & Ma, S. Iron Catalysis for Room-Temperature Aerobic Oxidation of Alcohols to Carboxylic Acids. *J. Am. Chem. Soc.* **138**, 8344-8347 (2016).

5 Shao, P., Wang, S., Chen, C. & Xi, C. Cp_2TiCl_2-Catalyzed Regioselective Hydrocarboxylation of Alkenes with CO_2. *Org. Lett.* **18**, 2050-2053 (2016).

6 Sano, K., Yamamoto, T. & Yamamoto, A. Preparation of Ni- or Pt-Containing Cyclic Esters by Oxidative Addition of Cyclic Carboxylic Anhydrides and Their Properties. *Bull. Chem. Soc. Jpn.* **57**, 2741-2747 (1984).

7 Bonaparte, A. C. et al. Novel Aerobic Oxidation of Primary Sulfones to Carboxylic Acids. *Org. Lett.* **13**, 1447-1449 (2011).

8 Qiu, J. C., Pradhan, P. P., Blanck, N. B., Bobbitt, J. M. & Bailey, W. F. Selective Oxoammonium Salt Oxidations of Alcohols to Aldehydes and Aldehydes to Carboxylic Acids. *Org. Lett.* **14**, 350-353 (2012).
